# An NHC‐Coordinated Silicon/Sulfur Analogue of an Acyl Carbene

**DOI:** 10.1002/anie.7380931

**Published:** 2026-07-10

**Authors:** Takuto Toyooka, Shintaro Ishida, Takeaki Iwamoto

**Affiliations:** ^1^ Department of Chemistry Graduate School of Science Tohoku University Sendai Japan

**Keywords:** carbene homologues, silanes, subvalent compounds, sulfur heterocycles

## Abstract

Despite great advances in the chemistry of low‐valent silicon species, a silicon/sulfur analogue of an acyl carbene, namely a silathioacyl silylene, has remained elusive. Herein, we report that the reaction of dialkyldisilyne **1** with cyclic thiourea **2a** affords **3**, an *N*‐heterocyclic carbene (NHC) complex of a silathioacyl silylene. The solid‐state structure of **3** reveals a three‐membered ring with a markedly elongated Si─S bond and an Si–Si single bond, consistent with hitherto unknown intramolecular coordination of the silanethione sulfur to the silylene center, while computational studies indicate negligible disilathiirene character. Compound **3** exhibits silylene‐like reactivity toward xylyl isocyanide to give an NHC complex of an imino‐substituted silanethione.

## Graphical abstract

An NHC complex of an Si/S analogue of an acyl carbene is synthesized by the reaction of a disilyne with a cyclic thiourea. The observed Si_2_S three‐membered ring with unsymmetrical Si─S distances and a Si─Si single bond is consistent with intramolecular coordination of the silathioacyl sulfur to the silylene center. The complex exhibits silylene‐like reactivity toward xylyl isocyanide.

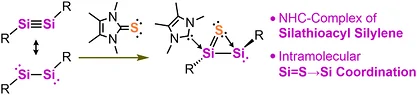

1

Acyl carbenes (**A**, Scheme 1a) represent a key class of reactive intermediates for the construction of various heterocycles [1, 2, 3, 4, 5, 6, 7, 8, 9, 10]. It readily undergoes a 1,2‐substituent shift (Wolff rearrangement) to provide the more stable ketene (**B**), which serves as a precursor to a variety of cyclic compounds as a reactive heteroallene. Moreover, **A** could generate oxirene **C**, an intriguing formally 4π‐antiaromatic three‐membered ring, and **C** is postulated to be an intermediate or a transition state en route to isomeric acyl carbene **A’**. Hence, the silicon/sulfur counterpart of an acyl carbene, namely silathioacyl silylene **D**, should also serve as a valuable platform for the synthesis of silicon‐ and sulfur‐containing heterocycles and low‐valent silicon sulfides, including disilathioketene **E** and disilathiirene **F**. Silathioacyl silylene **D** itself is an attractive synthetic target because it combines two different ambiphilic functionalities, a silylene center and a silanethione unit in close proximity. Nevertheless, access to **D** remains elusive despite substantial advances in stable silylenes and heavier ketone analogues [11, 12, 13, 14, 15, 16, 17, 18, 19, 20, 21, 22, 23, 24, 25, 26, 27, 28, 29, 30, 31, 32, 33]. Cui and co‐workers proposed silaacyl silylene **G** and its intramolecularly coordinated form **G’** as intermediates in the formation of the oxygen‐tethered bis(silylene) based on the computational study [24]. Previously, we reported that cyclic thioureas can serve as a source of both sulfur and *N*‐heterocyclic carbene (NHC), affording an NHC‐coordinated silanethione as the primary product in reactions with an isolable dialkylsilylene [25]. These findings prompted us to examine the sulfur‐transfer reactions of cyclic thioureas with a disilyne, which could behave as 1,2‐bis(silylene) [24, 34, 35, 36]. Herein, we report that the simultaneous sulfur transfer and NHC‐coordination of thioureas **2a** towards dialkyldisilyne **1** bearing 1,1‐bis(trimethylsilyl)‐3,3‐dimethylbutyl groups (hereafter abbreviated as Rs) to afford NHC complex of silathioacyl silylene **3** featuring an intramolecularly silanethione‐coordinated silylene (Scheme 1d) [34].

**SCHEME 1:**
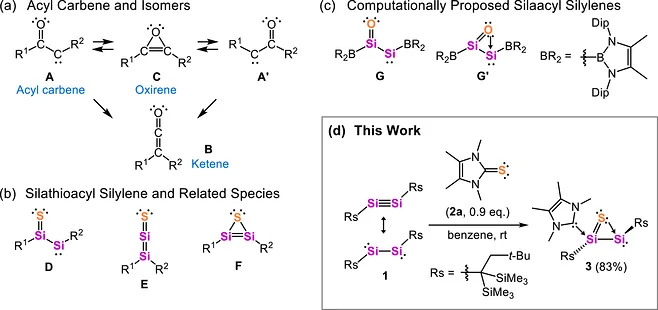
(a) Acyl carbene **A** and its isomers. Only singlet carbenes are drawn for clarity. (b) A silicon/sulfur analogue of acyl carbene (silathioacyl silylene) **D** and its isomers. (c) Silaacyl silylenes **G** and **G’** are calculated intermediates in a proposed reaction pathway. Dip = 2,6‐diisopropylphenyl. (d) This work: simultaneous sulfur transfer and NHC‐coordination of thioureas **2a** toward disilyne **1** to afford NHC‐coordinated silathioacyl silylene **3**.

Treatment of dialkyldisilyne **1** with 0.9 equiv. of 1,3,4,5‐tetramethylimidazoline‐2‐thione (**2a**) in benzene at room temperature afforded three‐membered ring compound **3** as an air‐ and moisture‐sensitive orange solid in 83% yield based on **2a** (Scheme 1d). No further reaction of **3** with **2a** was observed. Although **3** gradually decomposed in solution at room temperature and completely decomposed in benzene (10 mM) at 45°C for 2 days, the solid of **3** can be stored in a refrigerator in a glovebox for at least 6 months. The molecular structure of **3** was unambiguously determined by single‐crystal X‐ray diffraction (sc‐XRD) analysis (Figure 1). Notably, the two Si─S bonds on the three‐membered ring are markedly different. The Si2─S1 bond [2.3145(9) Å] is much longer than the standard Si─S single bond length (2.14 Å) [37], while the Si1─S1 bond [2.1289(8) Å] falls within the range between Si─S single bonds and those reported for NHC‐coordinated silanethiones (2.006–2.047 Å) [25, 38]. The Si1─Si2 bond length [2.3833(9) Å] is slightly longer than the standard Si─Si single bond length (2.36 Å) [39]. The C1 atom in the 1,3,4,5‐tetramethylimidazol‐2‐ylidene (IMe_4_) unit coordinates to the Si1 atom with a length of 1.950(3) Å. This length is longer than the sum of the single bond covalent radii of the silicon and carbon atoms (1.91 Å) [39] and close to the range reported for NHC‐coordinated silanethiones (1.963–1.976 Å) [25, 38]. The C1 atom on the IMe_4_ unit adopts a planar geometry [angle sum = 360.0(2)°], whereas the three‐coordinate Si2 atom is distinctly pyramidal [angle sum = 271.17(12)°].

**FIGURE 1:**
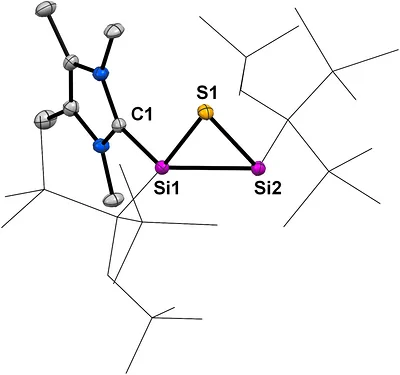
X‐ray structure of **3**. Thermal ellipsoids are drawn at the 50 % probability. Selected bond lengths [Å] and bond angles [°]: Si1−S1 2.1289(8), Si2−S1 2.3145(9), Si1−Si2 2.3833(9), Si1−C1 1.950(3), S1−Si1−Si2 53.87(2), Si1−S1−Si2 64.72(3), S1−Si2−Si1 61.41(3).

The NMR spectra of **3** indicate that this unsymmetrical structure is retained in solution. The ^1^H NMR spectrum of **3** in [D_6_]benzene exhibits four trimethylsilyl signals (0.13, 0.65, 0.73, and 0.76 ppm), which indicate two chemically inequivalent Rs groups and diastereotopic trimethylsilyl groups. The ^29^Si nuclei of the three‐membered ring resonate at −48.5 (Si1) and 4.4 (Si2) ppm in the ^29^Si{^1^H} NMR spectrum. In the ^13^C{^1^H} NMR spectrum, the resonance of C1 (159.8 ppm) is considerably upfield‐shifted compared to that of free NHC (IMe_4_, 212.7 ppm in benzene[D_6_]) [40]. This is consistent with the structurally observed coordination of the carbene unit. The electronic structure of **3** was further probed by UV–vis spectroscopy. The UV–vis spectrum of **3** in benzene exhibits its longest wavelength absorption band at 466 nm, which is assigned to the HOMO‐LUMO transition (vide infra) on the basis of TD‐DFT calculations (Figure S42 and Table S1).

Computational studies provided further insights into the electronic structure of **3**. The optimized structure (**3_opt_**) at the B3PW91‐D3/6‐31G(d) level of theory was in good agreement with the X‐ray structure. Mayer bond order (MBO) analysis of **3_opt_** revealed a marked difference between the two Si─S bonds: the MBO of Si1─S1 (1.01) is substantially larger than that of Si2─S1 (0.66) and is comparable to that of the Si─S bond in bis(trimethylsilyl)sulfide (0.98). These results support a bonding picture with an Si1–S1 single bond and a weaker Si2─S1 interaction. Natural resonance theory (NRT) analysis indicates that imidazolium‐disilene structure **I** is the dominant contributor (62.87%). Structure **II**, corresponding to a ylidic resonance form containing a free NHC and a silanethione (Si1═S1) unit, makes a smaller but significant contribution (15.88%), whereas the contribution of structure **III**, representing a free NHC and a disilathiirene, is negligible (0%). The highest occupied molecular orbital (HOMO) of **3_opt_** is primarily localized on the Si2 atom as a lone pair, and the LUMO is a delocalized π*‐orbital on the IMe_4_ ligand (Figure 2b). Taken together, these results indicate that **3** is best described as an NHC‐complex of a silathioacyl silylene with an intramolecular coordination of the silanethione sulfur to the silylene center. Comparison with the previously reported NHC complexes **4** and **5** (Figure 2c) [41, 42] highlights the unique bonding situation of **3**. Both **4** and **5** are derived from the corresponding cyclotrisilenes. In the case of compound **4**, the NHC molecule coordinates to the disilene moiety of a cyclotrisilene framework, whereas **5** is an NHC‐silylene complex via an Si─Si bond cleavage, which lacks an additional intramolecular donor‐acceptor interaction. Unlike **4** and **5**, silathiaacyl silylene **3** features simultaneous NHC→Si and intramolecular S→Si donor–acceptor interactions. This bonding situation appears to be enabled by the lone pairs of the sulfur atom.

**FIGURE 2:**
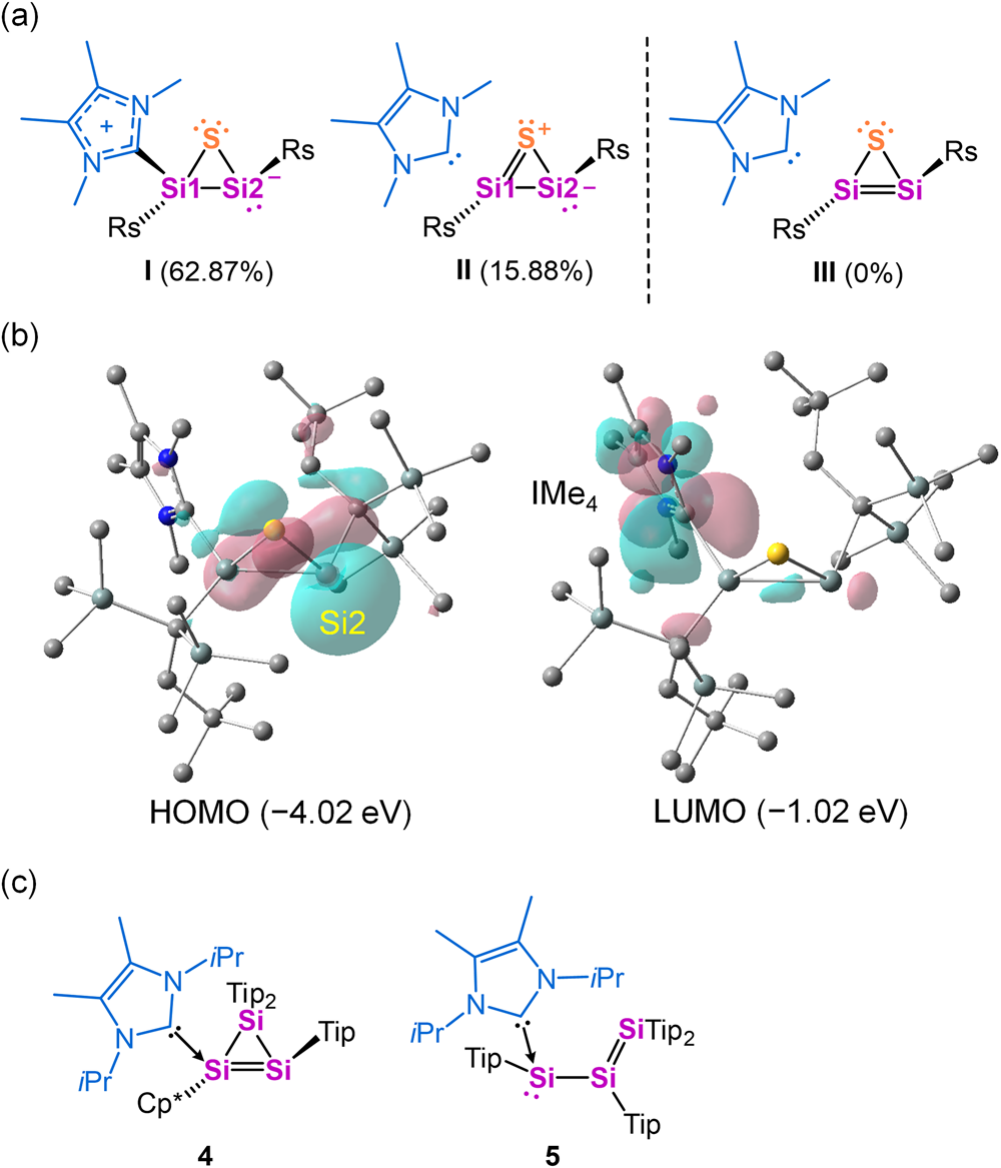
(a) Major canonical structures of **3_opt_** obtained by NRT analysis. Structure **I** represents a resonance hybrid of canonical structures whose positive charge is localized on the imidazolium ring (Figure S47). (b) Kohn–Sham HOMO and LUMO of **3_opt_** with energy levels calculated at the M06‐2X/6‐311+G(2df,p) level of theory (isosurface value: 0.04). (c) Related NHC‐complexes **4** and **5** [41, 42]. Tip = 2,4,6‐triisopropylphenyl, Cp* = *η* ^1^‐pentamethylcyclopentadienyl.

As energetic information on the R_2_Si_2_S and its isomers is still missing, calculations were performed on methyl‐substituted model compounds at the B3PW91‐D3/6‐31G(d) level of theory. Complex IMe_4_‐**3_Me_** is 198.7 kJ mol^−1^ lower in energy than silathioacyl silylene **3_Me_** and free IMe_4_ (Scheme 2a). A silathioacyl silylene without intramolecular coordination (Si═S→Si) could not be located as a minimum on the potential surface in our calculation. The structure of IMe_4_‐**3_Me_** essentially resembles that of **3_opt_**. These results indicate that both IMe_4_‐coordination and intramolecular silanethione‐to‐silylene coordination are strongly favored. We found planar disilathiirene to be a transition state (**TS1**) for the 1,2‐sulfur transfer process connecting enantiomeric silathioacyl silylenes (**3_Me_** and **3’_Me_**), with an activation barrier of 125.5 kJ mol^−1^, but we found a more facile reaction pathway between **3_Me_** and **3’_Me_**; a ring‐opening process to *C_2V_*‐1,3‐bis(silylene) **3_BS_** as an intermediate (Scheme 2b). **3_BS_** is only 11.1 kJ mol^−1^ above in energy from **3_Me_** with a small activation Gibbs energy (24.8 kJ mol^−1^). Thus, the 1,2‐sulfur shift in silathioacyl silylenes via disilathiirene is expected to be unfavorable. By contrast, 1,2‐methyl shift of **3_Me_** to furnish cyclic silylene **3_CS_** is a highly exergonic process (117.4 kJ mol^−1^) with a moderate activation Gibbs energy (47.4 kJ mol^−1^), indicating that cyclic silylenes can be formed readily from the corresponding silathioacyl silylenes (Scheme 2c). Although ring‐opening of **3_CS_** to disilathioketene **3_K_** is highly endergonic, **3_Me_** is only 6.6 kJ mol^−1^ lower in energy than **3_K_**. This energetic landscape in the silicon system differs from the pronounced thermodynamic preference generally observed for ketenes over the corresponding acyl carbenes [5]. These results are consistent with the isolation of **3** and with the absence of detectable disilathiirene‐type intermediates.

**SCHEME 2:**
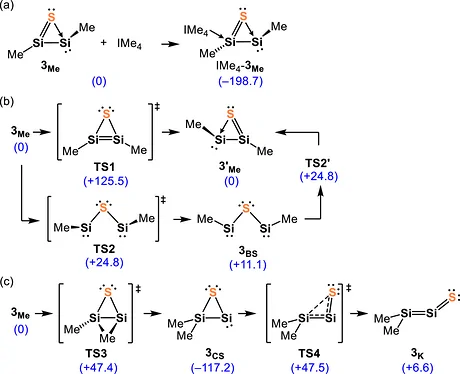
Gibbs energy diagrams of (a) complexation of **3_Me_** with IMe_4_ (IMe_4_ = 1,3,4,5‐tetramethylimidazol‐2‐ylidene), (b) 1,2‐sulfur migrations of **3_Me_** to **3’_Me_** via a disilathiirene as a transition state and a 1,2‐bis(silylene), and (c) **3_Me_** to disilathioketene **3_K_** via cyclic silylene **3_CS_**. Values in parentheses are relative energy in kJ mol–^1^.

Attempts to eliminate IMe_4_ from **3** by complexation with Lewis acids [BPh_3_, B(C_6_F_5_)_3_, and ZnCl_2_] to generate a base‐free silathioacyl silylene or its isomers have been unsuccessful at this stage. However, the silylene‐like reactivity of **3** was demonstrated by the reaction with xylyl isocyanide, which afforded a new NHC complex of imino‐silanethione **6** in 74% yield (Figure 3a), and the structure of **6** was confirmed by sc‐XRD. As reactions of silylenes with isocyanides to form silylene–isocyanide complexes or silaketenimines have been documented [43, 44], it is reasonable to propose that the coordination of xylyl isocyanide to the silylene center of **3** initially generates silaketenimine intermediate **7**. **7** could then undergo a 1,2‐silyl migration (silene–silylene rearrangement) [45] to give silylene **8**, followed by C–H insertion at the benzylic position to afford **6**. We investigated the 1,2‐silyl migration computationally using model compounds **7_Me_** and **8_Me_** at the B3PW91‐D3/6‐31G(d) level of theory; the isomerization from **7_Me_** to **8_Me_** is an exergonic process by 20.0 kJ mol^−1^ with a moderate activation Gibbs energy (+63.2 kJ mol^−1^), which supports the proposed mechanism.

**FIGURE 3:**
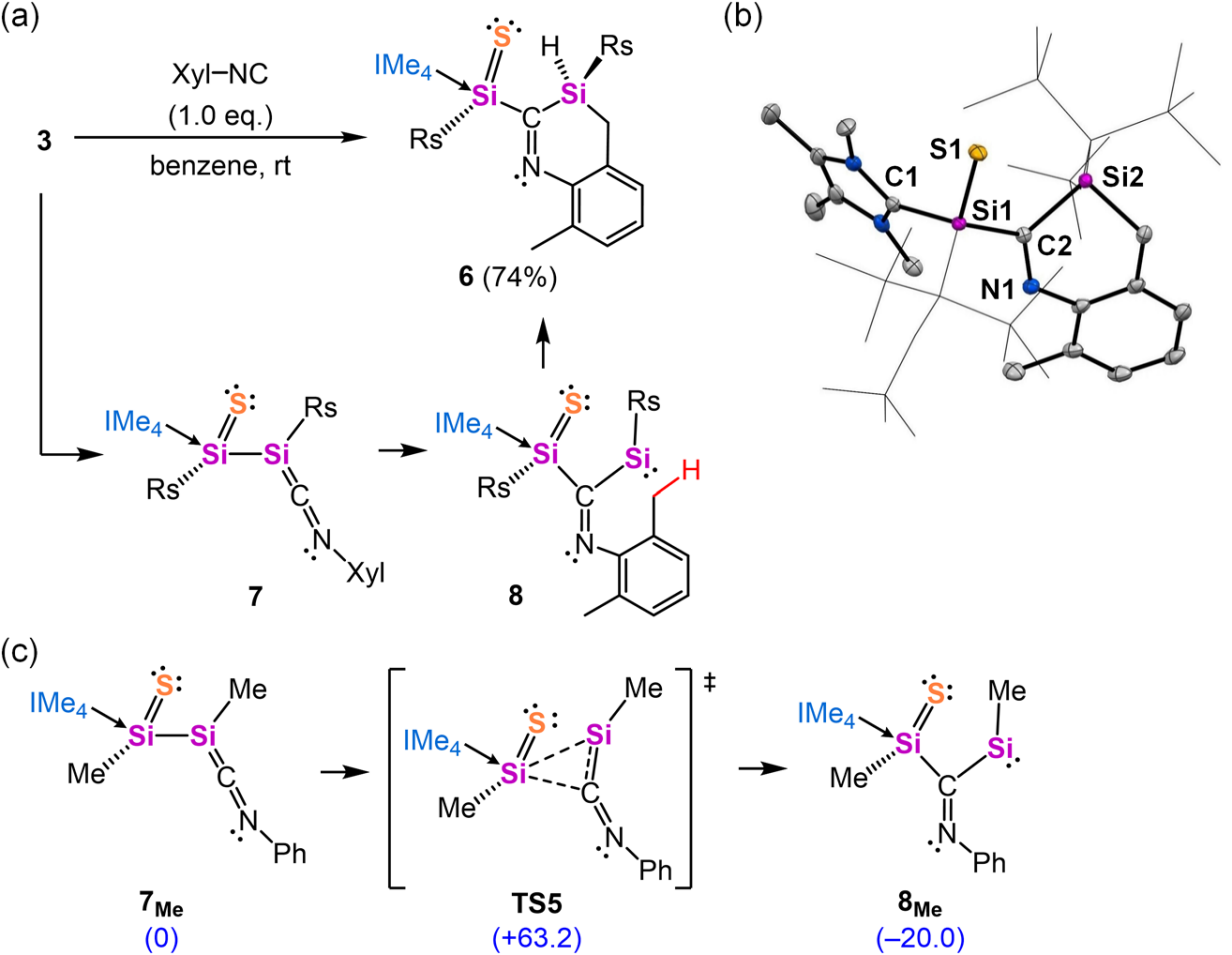
(a) Reaction of **3** with xylyl isocyanide to yield **6**, and its possible reaction mechanism. (b) X‐ray structure of **6**. Thermal ellipsoids are drawn at the 50 % probability. Selected bond lengths [Å]: Si1−S1 2.0289(6), Si1−C1 1.981(1), Si1−C2 1.962(1), Si2−C2 1.945(1), C2−N1 1.294(2). (c) 1,2‐Silyl migration process from **7_Me_** to **8_Me_** via **TS5**. Values in parentheses are relative energy in kJ mol–^1^.

The formation of the NHC complex of a silathioacyl silylene is highly sensitive to the structure of the cyclic thiourea. When **1** was treated with cyclic thiourea **2b** bearing a saturated backbone, silene **9b** was isolated in 19% yield (NMR yield: 60%, Figure 4). Similarly, treatment of **1** with cyclic thioamide **2c** afforded structurally related product **9c** in 51% isolated yield (NMR yield: 91%). Although no reaction intermediates were detected by NMR monitoring during the reactions, the structures of **9b** and **9c** suggest that a 1,2‐nitrogen migration from carbon to silicon is involved in the reaction sequence. Related 1,2‐substituent migrations from a bridging atom to a bridgehead silicon atom have been reported in the isomerization of tetrasilabicyclo[1.1.0]butane to the corresponding cyclotetrasilene [46, 47], and 1,3,4‐trisila‐2‐thia‐bicyclo[1.1.0]butanes are also known to undergo valence isomerization to furnish the corresponding cyclic disilenes [48, 49]. Based on these reports, a plausible mechanism for the formation of **9b** is outlined in Scheme 3. As NHC‐coordinated silanethione **10** is formed by the reaction of the corresponding dialkylsilylene with **2b** [25], NHC‐coordinated silathioacyl silylene **3b** would be initially formed. Then, the nucleophilic silicon in **3b** could attack the electrophilic carbon to form 1,3‐disila‐2‐thia‐bicyclo[1.1.0]butane **11**, followed by 1,2‐migration of the amino group to give **9b**. The sequence is supported by a computational study of methyl‐substituted model compounds (Figure S48). The formation of **9c** can be rationalized by a related sequence. In the case of **3**, formation of the corresponding 1,3‐disila‐2‐thia‐bicyclo[1.1.0]butane is presumably suppressed by the steric demand and charge delocalization over the NHC unit. Interestingly, treatment of **1** with 0.9 equiv of 1,3‐diisopropyl‐4,5‐dimethylimidazoline‐2‐thione (**2d**) in [D_6_]benzene at room temperature afforded four‐membered ring compound **12**, NHC‐coordinated silacycloprop‐1‐ylidene **13** [50, 51], and 1,1‐bis(trimethylsilyl)‐3,3‐dimethyl‐1‐butene **14** in 17%, 33%, and 26% NMR yields, respectively, based on **2d** (Scheme 4a). Although no intermediates were detected in this reaction and the detailed mechanism remains unclear, a plausible formation pathway is shown in Scheme 4b. The initially formed NHC‐coordinated silathioacyl silylene **3d** may undergo Si─Si bond cleavage, facilitated by the bulky NHC moiety, to generate NHC‐coordinated 1,3‐bis(silylene) **15**. Intramolecular C─H insertion at the uncoordinated silylene center could afford silacyclopropane **16** [34, 35]. Subsequent hydrogen transfer could produce silanethione‐coordinated silylene **17**. NHC transfer followed by dimerization of the resulting silanethione **18** would form **12**. The formation of **14** may be explained by the retro‐(1+2) cycloaddition of related silacyclopropane intermediates by NHC [35].

**FIGURE 4:**
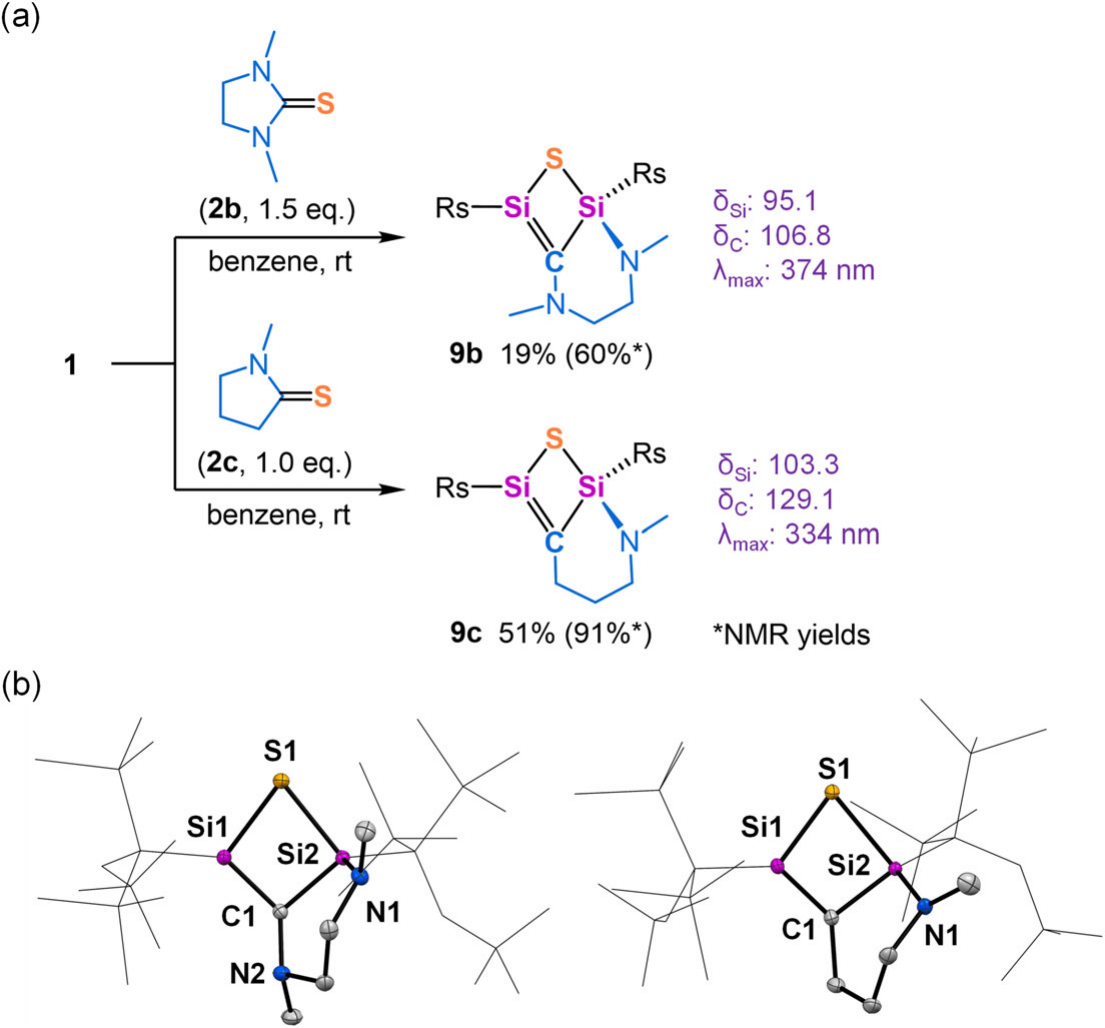
(a) Reactions of **1** with thiourea **2b** and thioamide **2c** to yield cyclic silene **9b** and **9c**. δ_Si_ and δ_C_ indicate ^29^Si and ^13^C NMR chemical shifts (in ppm) of the Si = C moiety in [D_6_]benzene. λ_max_ values are the longest wavelength absorption maxima measured in hexane at room temperature. (b) X‐ray structures of **9b** and **9c**. Thermal ellipsoids are drawn at the 50 % probability. Selected bond lengths [Å] and bond angles [°] **9b**: Si1−S1 2.1255(6), Si2−S1 2.1698(6), Si2−C1 1.862(1), Si1−S1−Si2 75.48(2), S1−Si1−C1 97.47(5), Si1−C1−Si2 94.52(7), S1−Si2−C1 91.48(5); **9c**: Si1−S1 2.1023(7), Si2−S1 2.2664(5), Si2−C1 1.838(1), Si1−S1−Si2 73.57(2), S1−Si1−C1 100.28(4), Si1−C1−Si2 94.84(6), S1−Si2−C1 91.06(4).

**SCHEME 3:**
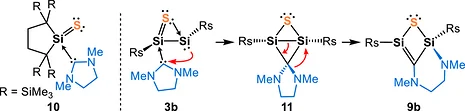
NHC‐coordinated silanethione **10** and a possible formation mechanism of **9b**.

**SCHEME 4:**
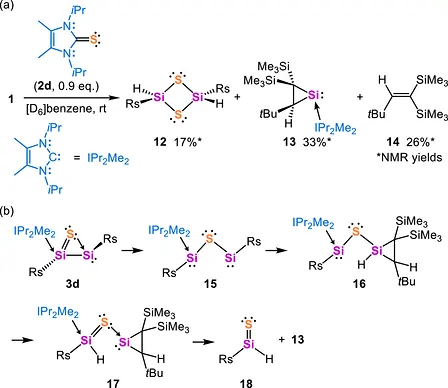
Reaction of **1** with thiourea **2d**.

In conclusion, the three‐membered ring compound **3** was synthesized via the sulfur‐transfer reaction from cyclic thiourea **2a** to disilyne **1**. The molecular structure, spectroscopic properties, and computational studies showed that **3** is best described as an NHC complex of a silathioacyl silylene with an intramolecular coordination of the silanethione sulfur to the silylene center, while a disilathiirene character is negligible. Reaction of **3** with xylyl isocyanide afforded imino‐silanethione **6**. The formation of cyclic silenes **9b** and **9c** from the reactions of **1** with **2b** and **2c** suggests that steric and electronic stabilization by the NHC unit is crucial for the isolation of the NHC complex of a silathioacyl silylene. These findings may pave the way for access to hitherto elusive heavier analogues of oxirene and thiirene, as well as their ring‐opened counterparts corresponding to acyl and thioacyl carbene analogues.

## Author Contributions

**Takuto Toyooka**: investigation, validation, visualization, writing – original draft, writing – review & editing. **Shintaro Ishida**: conceptualization, data curation, investigation, formal analysis, validation, funding acquisition, visualization, project administration, resources, writing – original draft, writing – review & editing. **Takeaki Iwamoto**: project administration, writing – review & editing, resources, and supervision.

## Conflicts of Interest

The authors declare no conflicts of interest.

## Supporting information




**Supporting File**: The authors have cited additional references within the Supporting Information [52, 53, 54, 55, 56, 57, 58, 59, 60, 61, 62, 63, 64, 65]. Crystallographic data for the structures reported in this article have been deposited at the Cambridge Crystallographic Data Centre [66].

## Data Availability

The data that supports the findings of this study are available in the supplementary material of this article
