# Supplementary material for: An NHC‐Coordinated Silicon/Sulfur Analogue of an Acyl Carbene
Source: Angew Chem Int Ed Engl. 2026 Jul 10;65(35):e7380931. doi: 10.1002/anie.7380931 (PMC13502560; doi:10.1002/anie.7380931)
Supplement: Supplementary file 1 [file ANIE-65-e7380931-s001.pdf]

Supporting Information

An NHC-Coordinated Silicon/Sulfur Analogue of an Acyl Carbene

Takuto Toyooka, Shintaro Ishida,* and Takeaki Iwamoto*

Table of Content

1. Experimental Details S2

2. NMR Spectra S6

3. X-ray Structural Analysis S24

4. UV-vis Absorption Spectra S27

5. Theoretical Studies S28

1. Experimental Details

General Procedures

All reactions treating air-sensitive compounds were carried out under inert atmosphere (N_2 or Argon) using a high vacuum line, standard Schlenk techniques, a glove box, and dry- and oxygen-free solvents. NMR spectra were recorded on a Bruker Avance 400 or 500 FT NMR spectrometers. The ^{1}H NMR chemical shifts in C_6D_6 were referenced to residual C_6D_5H signal (^{1}H δ 7.16). The ^{13}C{^{1}H} NMR and ^{29}Si{^{1}H} NMR chemical shifts in C_6D_6 were relative to Me_4Si (δ 0.00). Sampling of air-sensitive compounds was carried out using a VAC NEXUS 100027 type glove box. Mass spectra were recorded on a Bruker Daltonics SolariX 9.4T for APCI method, a JEOL JMS-T100GCV for FD method, or a JEOL JMS-Q1050 for EI method. Elemental analyses were performed on a J-SCIENCE LAB JM-11 for CHN elements at Research and Analytical Center for Giant Molecules (Graduate School of Science, Tohoku University). Melting points (mp.) were measured by an OptiMelt MPA100 automated melting point system.

Materials

Benzene, hexane, toluene, and THF were dried by using a VAC 103991 solvent purifier. Et_2O was distilled over lithium aluminum hydride and degassed prior to use. Benzene-*d*$_6$ (C_6D_6) was dried over molecular sieves MS 4Å. Dialkyldisilyne **1**[34], 1,3,4,5-tetramethylimidazole-2-thione **2a**[52], 1,3-dimethylimidazolidine-2-thione **2b**[53], 1-methylimidazolidine-2-thione **2c**[54], 1,3-diisopropyl-4,5-dimethylimidazole-2-thione **2d**[55] and xylyl isocyanide[56] were prepared according to the published procedures.

Reaction of Disilyne 1 with Cyclic Thiourea 2a

In a glass vial (5 mL) equipped with a magnetic stir bar, disilyne **1** (130 mg, 0.25 mmol) and 1,3,4,5-tetramethylimaidazole-2-thione **2a** (37 mg, 0.24 mmol) were charged. To this vial, benzene (4 mL) was added, and the mixture was stirred for 5 minutes. After the solvent was removed in vacuo, an orange solid was obtained. The solid was washed with hexane (2 mL ×2), and analytically pure **3** was obtained in 83% yield based on **2a** (133 mg, 0.20 mmol) as an orange solid. As the thermal decomposition of **1**[34] competes with its reaction with **2a**, a small amount of unreacted **2a** remained after consumption of **1** in the stoichiometric reaction between **1** and **2a**. Product **3** could be readily isolated from bi(silacyclopropane)s, whereas it could not be separated from unreacted **2a** by recrystallization under various conditions. Therefore, 0.9 equiv of **2a** was employed to ensure complete consumption of **2a**.

3: orange solid; mp. 86-89 °C (decomp.); ^{1}H NMR (500 MHz, 296 K, C_6D_6): δ 0.13 (brs, 9H, $SiMe_3$), 0.65 (s, 9H, $SiMe_3$), 0.67 (s, 9H, *t*-Bu), 0.73 (s, 9H, $SiMe_3$), 0.76 (s, 9H, $SiMe_3$), 0.87 (d, 1H, J = 15.0 Hz, CH_2), 1.25 (s, 3H, Me), 1.30 (s, 3H, Me), 1.33 (s, 9H, *t*-Bu), 1.79 (d, 1H, J = 14.9 Hz, CH_2), 2.00 (d, 1H, J = 14.9 Hz, CH_2), 2.08 (d, 1H, J = 14.9 Hz, CH_2), 3.65 (s, 3H, NMe), 4.00 (s, 3H, NMe); ^{13}C{^{1}H} NMR (126 MHz, 297 K, C_6D_6): δ 4.4 (br, $SiMe_3$), 5.0 ($SiMe_3$), 5.9 ($SiMe_3$), 8.0 ($SiMe_3$), 8.3 (Me), 8.6 (Me), 15.1 (C), 21.8 (C), 32.12 (Me), 32.14 (C), 33.8 (Me), 34.8 (C), 36.9 (NMe), 37.0 (NMe), 45.2 (CH_2), 50.1 (CH_2), 125.7 (C=C, NHC), 128.2 (C=C, NHC)*, 159.8 (carbene, NHC); ^{29}Si{^{1}H} NMR (99 MHz, 295 K, C_6D_6): δ −48.5 (Si←IMe_4), −1.5 ($SiMe_3$), −1.4 ($SiMe_3$), 1.5 ($SiMe_3$), 2.8 ($SiMe_3$), 4.4 (br, Si); HRMS-APCI (*m/z*): $[M]^+$ calcd for $C_{31}H_{70}N_2SSi_6$, 670.3870; found 670.3870; UV-vis (benzene) λ_{max}/nm (ε): 466 (1600), 318 (5000); Anal. calcd for $C_{31}H_{70}N_2SSi_6$: C, 55.43, H, 10.64, N, 4.46%, found: C, 55.45, H, 10.51, N, 4.17%. *This signal overlapped with the signals of C_6D_6 and was confirmed by ^{1}H-^{13}C HMBC spectrum.

Reaction of 3 with Xylyl Isocyanide

In a glass vial (5 mL) equipped with a magnetic stirring bar, **3** (72 mg, 0.11 mmol) and 2,6-dimethylphenylisocyanide (14 mg, 0.11 mmol) were charged. In the vial, benzene (1 mL) was added, and the mixture was stirred for 5 minutes at room temperature. After the solvent was removed in vacuo, the obtained solid was washed with hexane (5 mL). Then, analytically pure **6** was obtained in the 74% yield (63 mg, 0.078 mmol) as a yellow solid.

6: yellow solid; mp. 114-116 °C (decomp.); ^{1}H NMR (500 MHz, C_6D_6, 296 K): δ 0.25 (s, 9H, $SiMe_3$), 0.34 (s, 9H, $SiMe_3$), 0.63 (s, 9H, $SiMe_3$), 0.53-0.84 (brs, 9H, $SiMe_3$), 0.71 (s, 9H, *t*-Bu), 1.20 (s, 9H, *t*-Bu), 1.28 (s, 3H, Me, NHC), 1.30 (s, 3H, Me, NHC), 1.48 (d, J = 15.0 Hz, 1H, CH_2), 1.86 (d, J = 15.0 Hz, 1H, CH_2), 1.96 (d, J = 14.0 Hz, 1H, CH_2), 2.23 (d, J = 16.3 Hz, 1H, CH_2), 2.55 (s, 3H, Me), 2.80 (dd, J = 16.2, 2.9 Hz, 1H, CH_2), 3.89 (d, J = 14.0 Hz, 1H, CH_2), 4.05 (s, 3H, NMe), 4.56 (s, 3H, NMe), 6.09 (d, 1H, J = 3.2 Hz, J_{SiH} = 234.2 Hz, SiH), 6.95 (m, 2H, CH), 7.04 (m, 1H, CH); ^{13}C{^{1}H} NMR (126 MHz, C_6D_6, 297 K): δ 4.1 ($SiMe_3$), 4.9 ($SiMe_3$), 7.9 ($SiMe_3$), 8.4 (Me), 8.7 (Me), 13.7 (C), 18.6 (CH_2), 20.4 (Me), 22.6 (C), 32.0 (Me), 32.7 (C), 33.0 (Me), 34.0 (C), 37.8 (NMe), 37.9 (NMe), 43.4 (CH_2), 43.8 (CH_2), 125.4 (C), 125.5 (C=C, NHC), 127.4 (CH), 129.2 (CH + C=C, NHC), 130.3 (CH), 134.7 (C), 147.1 (*ipso*-C), 154.4 (carbene, NHC), 219.6 (C=N); ^{29}Si{^{1}H} NMR (99 MHz, C_6D_6, 296 K): δ −60.4 (SiS), −11.0 (SiH), −0.3 ($SiMe_3$), 0.1 ($SiMe_3$), 0.6 ($SiMe_3$), 2.9 ($SiMe_3$); HRMS-APCI (*m/z*): $[M+H]^+$ calcd for $C_{40}H_{80}N_3SSi_6$, 802.4683; found 802.4684; Anal. calcd for $C_{40}H_{79}N_3SSi_6$: C, 59.86, H, 9.92, N, 5.24%, found: C, 59.99, H, 9.87, N 5.35%.

Reaction of Disilyne 1 with Cyclic Thiourea 2b

In a glass vial (10 mL) equipped with a magnetic stirring bar, disilyne **1** (118 mg, 0.23 mmol) and 1,3-dimethylimiadzolidine-2-thione **2b** (46 mg, 0.35 mmol) were charged. To this vial, benzene (2 mL) was added at room temperature, and the mixture was stirred for 30 minutes. The solvent was removed in vacuo, and the residue was dissolved in hexane. The resulting insoluble materials were filtered off. Concentration of the filtrate afforded unreacted **2b** as a white precipitate. After removal of **2b** by filtration, the volatiles were removed under reduced pressure. The resulting crude product was recrystallized twice from toluene to afford **9b** in 19% yield (28 mg, 0.044 mmol) as a yellow solid.

9b: yellow solid; mp. 145 °C (decomp.); ^{1}H NMR (500 MHz, C_6D_6, 295 K): δ 0.41 (s, 9H, $SiMe_3$), 0.43 (s, 9H, $SiMe_3$), 0.53 (s, 9H, $SiMe_3$), 0.66 (s, 9H, $SiMe_3$), 1.18 (s, 9H, *t*-Bu), 1.22 (s, 9H, *t*-Bu), 1.92 (d, J = 14.9 Hz, 1H, CH_2), 2.07 (d, J = 14.8 Hz, 1H, CH_2), 2.39 (d, J = 14.8 Hz, 1H, CH_2), 2.59 (d, J = 14.8 Hz, 1H, CH_2)*, 2.60 (s, 3H, NMe), 2.57-2.63 (m, 1H, NCH_2), 2.70 (s, 3H, NMe), 2.95-2.97 (m, 2H, NCH_2), 3.09-3.15(m, 1H, NCH_2); ^{13}C{^{1}H} NMR (126 MHz, C_6D_6, 296 K): δ 3.0 ($SiMe_3$), 3.5 ($SiMe_3$), 5.9 ($SiMe_3$), 6.6 ($SiMe_3$), 17.8 (C), 24.4 (C), 32.0 (Me), 32.6 (Me), 33.6 (C), 34.1 (C), 41.4 (NMe), 44.8 (CH_2), 46.9 (NMe), 47.2 (CH_2), 55.6 (NCH_2), 58.8 (NCH_2), 129.1 (Si=C); ^{29}Si{^{1}H} NMR (99 MHz, C_6D_6, 296 K): δ −0.4 (SiN), −0.1 ($SiMe_3$), 1.6 ($SiMe_3$), 2.6 ($SiMe_3$), 3.2 ($SiMe_3$), 103.3 (Si=C); HRMS-FD (*m/z*): $[M]^+$ calcd for $C_{29}H_{68}N_2SSi_6$, 644.3719; found 644.3718; UV-vis (hexane) λ_{max}/nm (ε): 374 (2500); Anal. calcd for $C_{29}H_{68}N_2SSi_6$: C, 53.97, H, 10.62, N, 4.34%, found: C, 54.24, H, 10.56, N 4.43%. *This signal overlapped with the signal of NMe, which was confirmed by ^{1}H-^{13}C HSQC spectrum.

Reaction of Disilyne 1 with 2b in an NMR Tube

In a J. Young NMR tube, disilyne **1** (28 mg, 0.054 mmol) and 1,3-dimethylimiadzolidine-2-thione **2b** (11 mg, 0.083 mmol) were placed. To the tube, C_6D_6 (0.5 mL) were added at room temperature. After 40 minutes, the formation of cyclic silene **9b** (0.033 mol, 60% yield) was confirmed by ^{1}H NMR spectrum. The yields of the products were determined by ^{1}H NMR integrals using 1,3,5-tri-*tert*-butylbenzene as an internal standard.

Reaction of Disilyne 1 with Cyclic Thioamide 2c

In a glass vial (5 mL), disilyne **1** (110 mg, 0.21 mmol) was placed. To the vial, a benzene (4 mL) solution of 1-methylpyrrolidine-2-thione **2c** (24 mg, 0.21 mmol) were added at room temperature, and the mixture was stirred for 5 minutes. The solvent was

removed in vacuo and recrystallization from Et_2O for about 2 weeks afforded **9c** in 51% yield (67 mg, 0.11 mmol) as a pale-yellow solid.

9c: pale-yellow solid; mp. 72-78 °C (decomp.); ^{1}H NMR (500 MHz, C_6D_6, 296 K): δ 0.35 (s, 9H, $SiMe_3$), 0.36 (s, 9H, $SiMe_3$), 0.557 (s, 9H, $SiMe_3$), 0.563 (s, 9H, $SiMe_3$), 1.17 (s, 9H, *t*-Bu), 1.20 (s, 9H, *t*-Bu), 1.77-1.84 (m, 1H, CH_2), 1.92 (d, J = 14.9 Hz, 1H, CH_2), 2.03 (d, J = 14.9 Hz, 1H, CH_2), 1.98-2.09 (m, 1H, CH_2), 2.34 (d, J = 14.8 Hz, 1H, CH_2), 2.48 (d, J = 14.8 Hz, 1H, CH_2), 2.51-2.58 (m, 1H, CH_2), 2.71-2.79 (m, 2H, CH_2 and NCH_2), 2.77 (s, 3H, NMe), 2.99 (dt, J = 11.6, 1.8 Hz, 1H, NCH_2); $^{13}C\{^1H\}$ NMR (126 MHz, C_6D_6, 296 K): δ 2.8 ($SiMe_3$), 3.6 ($SiMe_3$), 5.7 ($SiMe_3$), 5.9 ($SiMe_3$), 19.8 (C), 23.2 (C), 28.8 (CH_2), 29.8 (CH_2), 32.2 (Me), 32.9 (Me), 33.7 (C), 34.0 (C), 42.2 (NMe), 44.8 (CH_2), 45.7 (CH_2), 54.4 (NCH_2), 106.8 (Si=C); $^{29}Si\{^1H\}$ NMR (99 MHz, C_6D_6, 296 K): δ −0.5 (SiN), 0.6 ($SiMe_3$), 1.9 ($SiMe_3$), 2.8 ($SiMe_3$), 3.0 ($SiMe_3$), 95.1 (Si=C); HRMS-APCI (*m/z*): $[M+H]^+$ calcd for $C_{29}H_{68}NSSi_6$, 630.3683; found 630.3683; UV-vis (hexane) λ_{max}/nm (ε): 334 (4200); Anal. calcd for $C_{29}H_{67}NSSi_6$: C, 55.25, H, 10.71, N, 2.22%, found: C, 54.98, H, 10.66, N 2.26%.

Reaction of Disilyne 1 with 2c in an NMR Tube

In a J. Young NMR tube, disilyne **1** (54 mg, 0.10 mmol) and 1-methylpyrroridine-2-thione **2b** (12 mg, 0.10 mmol) were placed. To the tube, C_6D_6 (0.6 mL) were added at room temperature. After 5 minutes, the formation of cyclic silene **9c** (0.091 mol, 91% yield) was confirmed by ^{1}H NMR spectrum. The yields of the products were determined by ^{1}H NMR integrals using 1,3,5-tri-*tert*-butylbenzene as an internal standard.

Reaction of 1 with Cyclic Thiourea 2d

Because this reaction competes with the thermal decomposition of **1** and residual **2d** complicates the isolation process, 0.8 equiv of **2d** relative to **1** was employed to ensure complete consumption of **2d**. In a 5 mL glass vial equipped with a magnetic stir bar, disilyne **1** (123 mg, 0.24 mmol) and 1,3-diisopropyl-4,5-dimethylimidazole-2-thione **2d** (41 mg, 0.19 mmol) were placed under an argon atmosphere. Benzene (2 mL) was added to the vial, and the mixture was stirred for 1 h at room temperature. After removal of the solvent under reduced pressure, the resulting residue was extracted with hexane and filtered to remove insoluble solids. The volatiles were removed from the filtrate under reduced pressure, and the resulting solid was recrystallized from a THF/Et_2O (1:2, v/v) mixture. The obtained crystals were washed twice with hexane to afford analytically pure **12** (16 mg, 0.028 mmol) as a colorless solid in 14% yield based on **2d**. After removal of the solvent from the combined mother liquors under reduced pressure, crude **13** was recrystallized twice from toluene to afford **13** (17 mg, 0.038 mmol) contaminated with 1.0 mol% of **12** as a colorless solid in 20% yield based on **2d**.

12: colorless solid; mp. 232-235 °C; ^{1}H NMR (400 MHz, 298 K, C_6D_6): δ 0.42 (s, 36H, $SiMe_3$), 1.12 (s, 18H, *t*-Bu), 2.17 (s, 4H, CH_2), 6.29 (s, $J_{Si\text{-}H}$ = 248 Hz, 2H, SiH); $^{13}C\{^1H\}$ NMR (126 MHz, 295 K, C_6D_6): δ 3.5 ($SiMe_3$), 15.0 (C), 32.1 (Me), 33.8 (C), 44.7 (CH_2); $^{29}Si\{^1H\}$ NMR (99 MHz, 295 K, C_6D_6): δ −3.2 (SiH), 5.1 ($SiMe_3$); HRMS-APCI (*m/z*): $[M+H]^+$ calcd for $C_{24}H_{61}S_2Si_6$, 581.2825; found 581.2825; Anal. calcd for $C_{24}H_{60}S_2Si_6$: C, 49.58, H, 10.40%, found: C, 49.36, H, 10.39%.

13: colorless solid; mp. 120 °C (decomp.); ^{1}H NMR (400 MHz, 299 K, C_6D_6): δ 0.21 (s, 9H, $SiMe_3$), 0.71 (s, 9H, $SiMe_3$), 1.08 (d, J = 7.1 Hz, 3H, Me), 1.13 (d, J = 7.0 Hz, 3H, Me), 1.21 (d, J = 7.2 Hz, 3H, Me), 1.30 (d, J = 6.9 Hz, 3H, Me), 1.43 (s, 3H, Me), 1.44 (s, 15H, *t*-Bu + Me), 2.03 (s, 1H, CH), 5.65 (sep, J = 7.0 Hz, 1H, CH), 6.14 (sep, J = 7.2 Hz, 1H, CH); $^{13}C\{^1H\}$ NMR (126 MHz, 296 K, C_6D_6): δ 4.0 ($SiMe_3$), 6.8 ($SiMe_3$), 9.3 (C), 9.8 (Me), 10.1 (Me), 20.7 (Me), 21.2 (Me), 21.3 (Me), 21.5 (Me), 33.3 (C), 34.5 (Me), 45.5 (CH), 51.1 (CH), 52.1 (CH), 125.2 (C=C), 125.3 (C=C), 173.3 (N–C–N); $^{29}Si\{^1H\}$ NMR (99 MHz, 295 K, C_6D_6): δ −140.1 (SiC_2), −6.5 ($SiMe_3$), 0.8 ($SiMe_3$); HRMS-APCI (*m/z*): $[M+H]^+$ calcd for $C_{34}H_{49}N_2Si_3$, 437.3198; found 437.3199.

Reaction of 1 with 2d in an NMR Tube

We used 0.9 equiv of **2d** relative to **1**. In a J. Young NMR tube, **1** (33 mg, 64 μmol) and **2d** (12 mg, 58 μmol) were placed. To the

tube, C_6D_6 (0.5 mL) and 1,3,5-trimethoxybenzene as an internal standard were added at room temperature. After 1 hour, the formation of **12** (10 µmol, 17%), **13** (19 µmol, 33%), 1,1-bis(trimethylsilyl)-3,3-dimethyl-1-butene **14** (15 µmol, 26%), and thermal isomerization products of disilyne **1** (3 µmol, 6%) were confirmed by ^{1}H NMR spectrum. Yields of products were based on **2d**. Unreacted **2d** was also detected. The yields of the products were determined by ^{1}H NMR integrals.

2. NMR Spectra

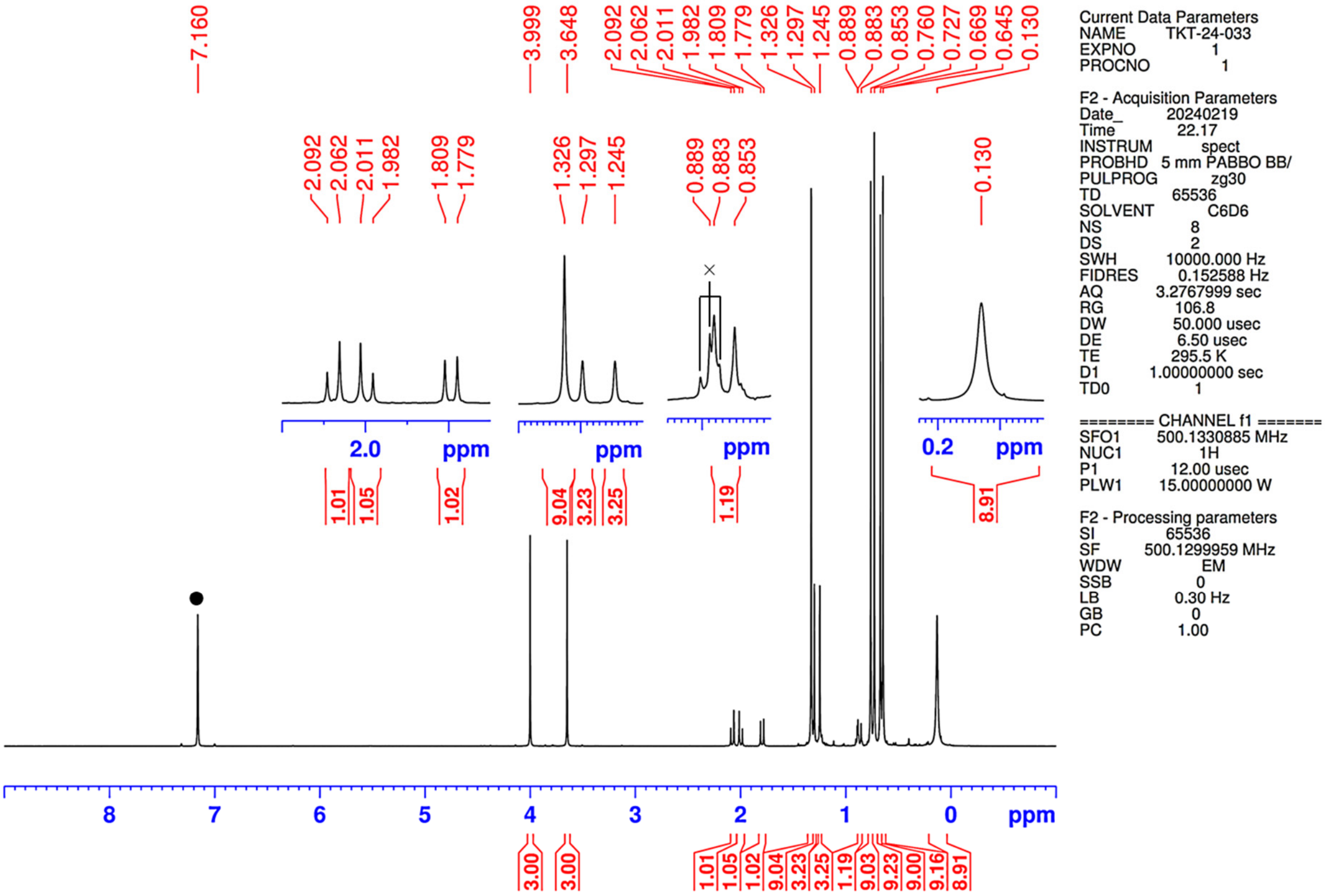

Figure S1. ^{1}H NMR spectrum of **3** in C_6D_6 at 296 K (● = C_6D_5H, × = hexane).

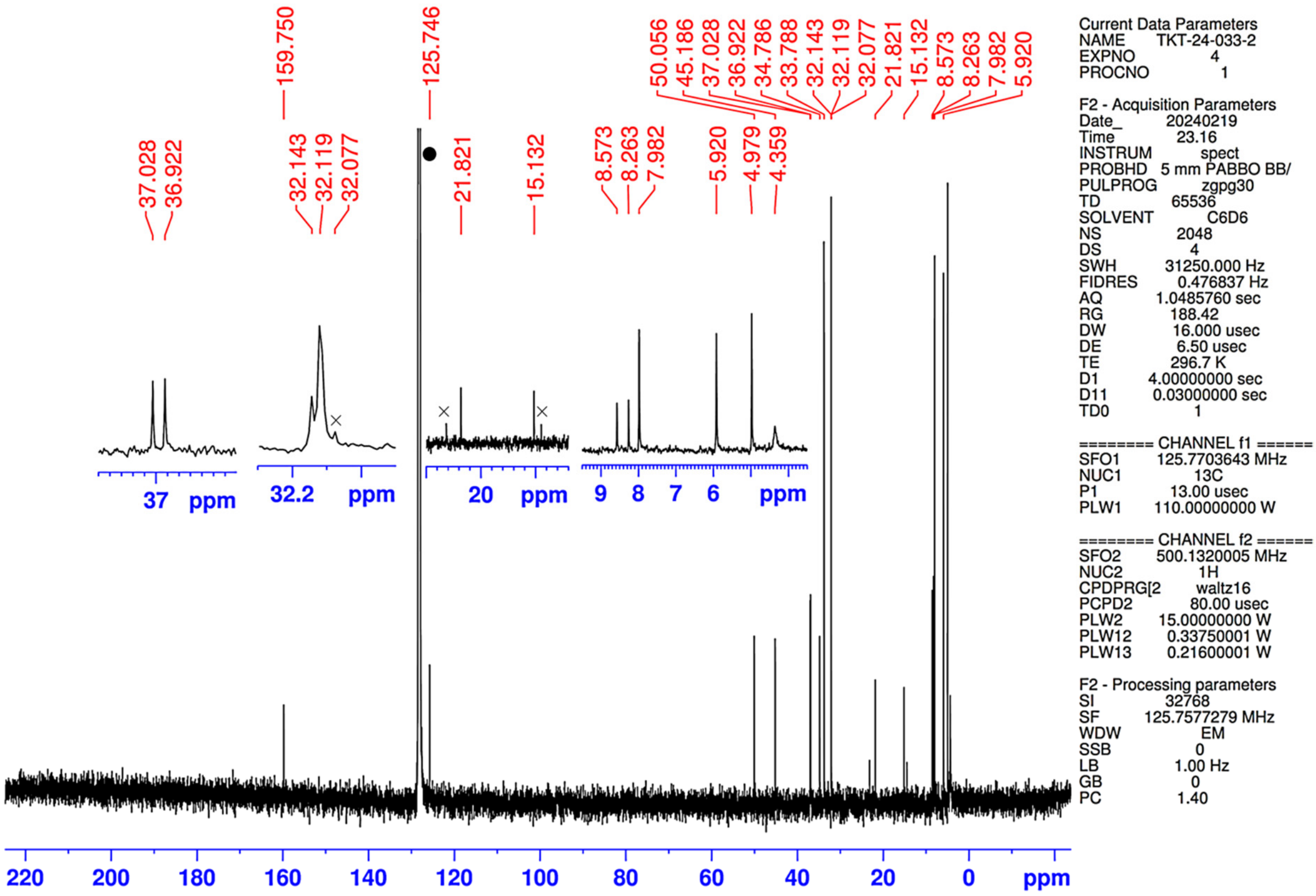

Figure S2. $^{13}C\{^{1}H\}$ CPD NMR spectrum of **3** in C_6D_6 at 297 K (● = C_6D_6, × = hexane).

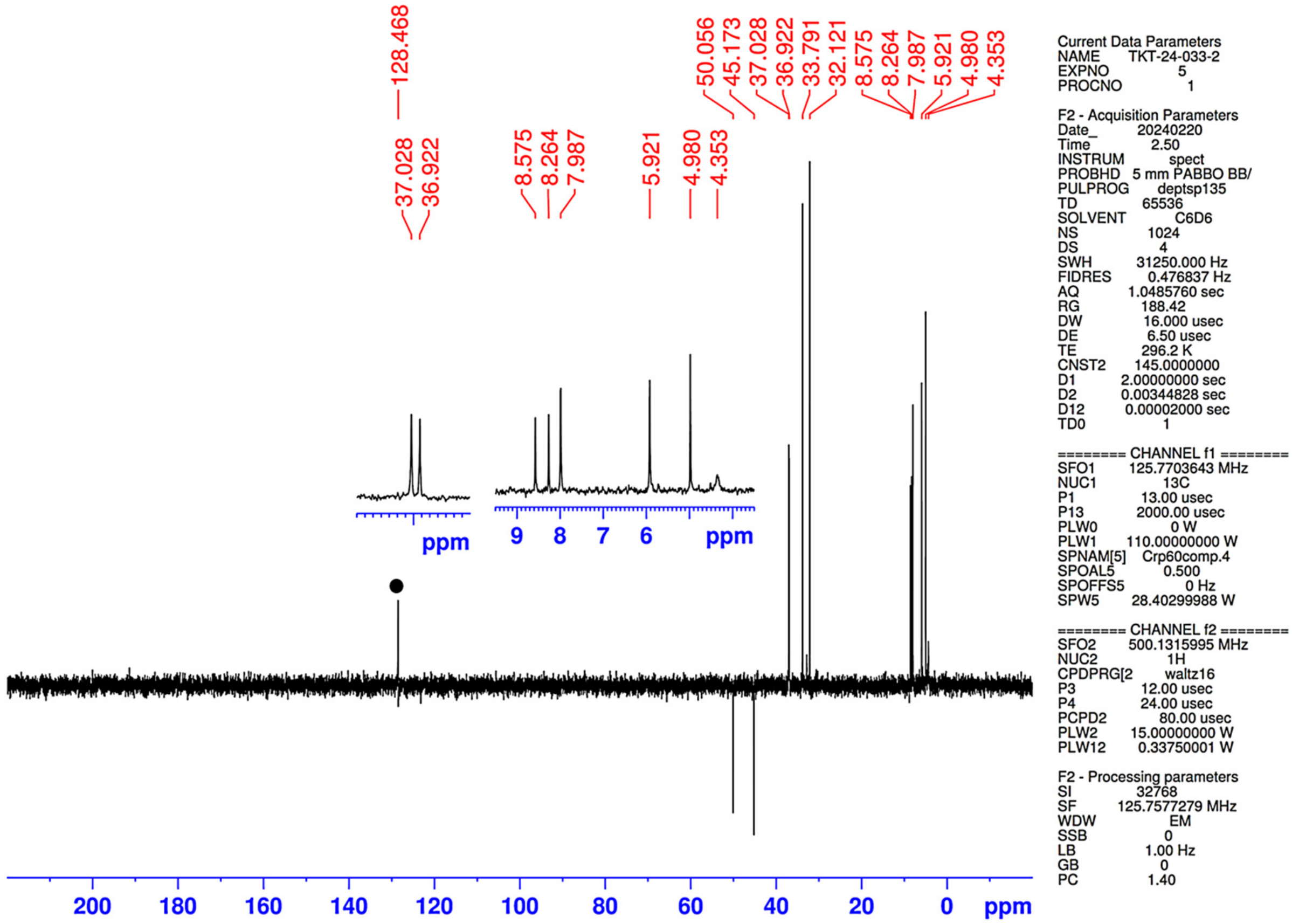

Figure S3. $^{13}C\{^1H\}$ DEPT 135 NMR spectrum of **3** in C_6D_6 at 296 K (● = C_6D_5H).

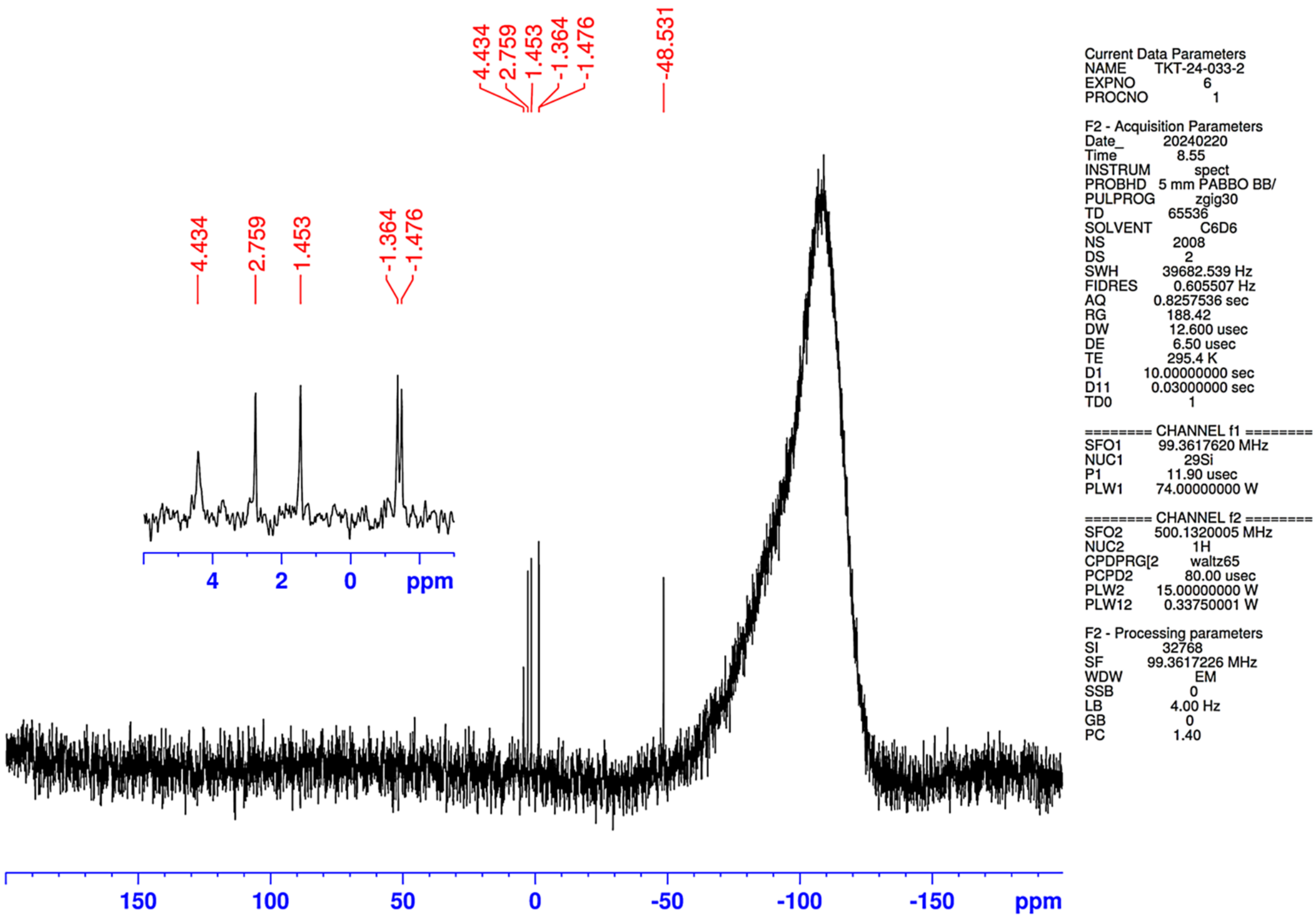

Figure S4. $^{29}Si\{^1H\}$ NMR spectrum of **3** in C_6D_6 at 295 K.

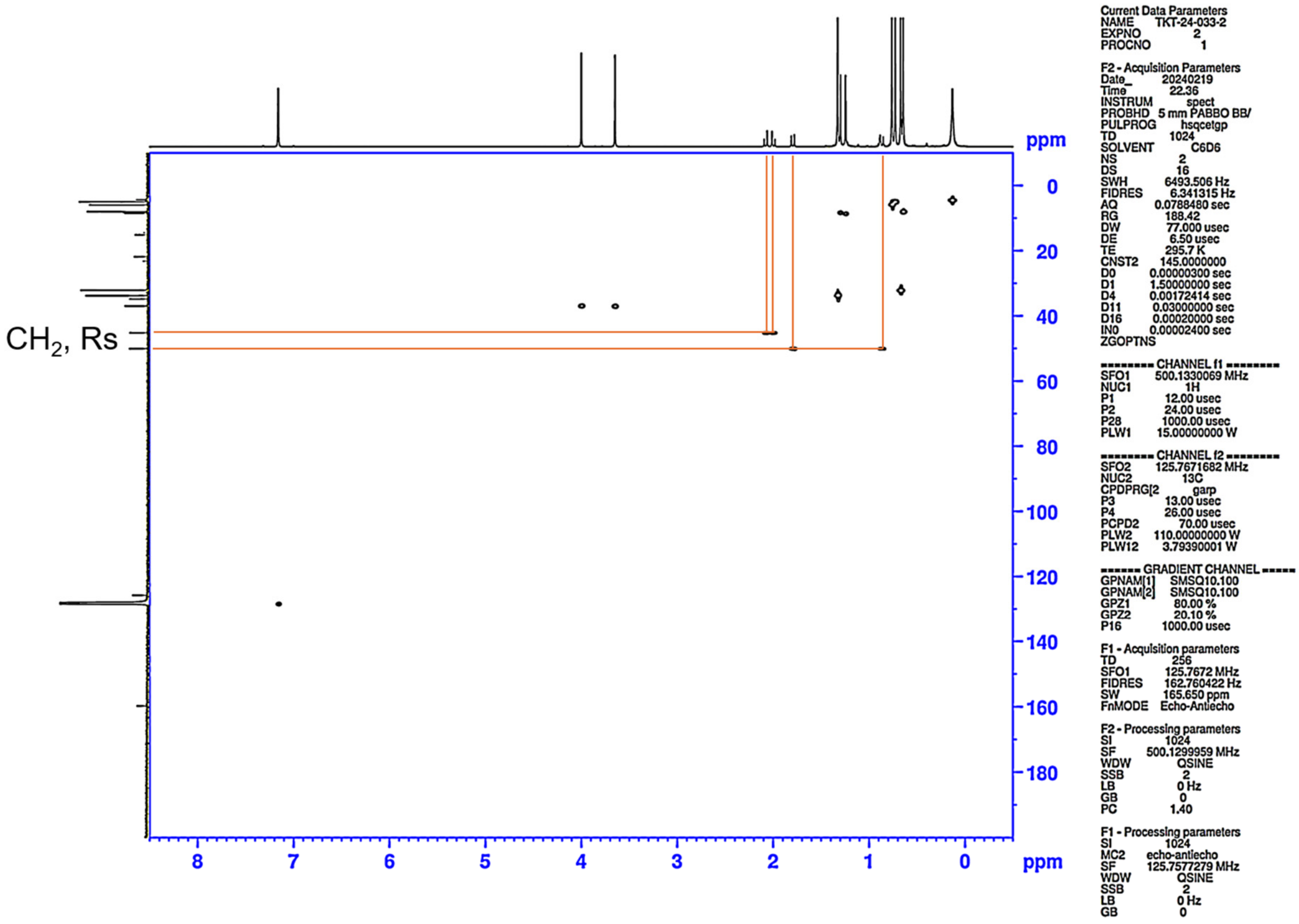

Figure S5. ^{1}H-^{13}C HSQC NMR spectrum of **3** in C_6D_6 at 296 K. Geminal protons were confirmed by cross-peaks.

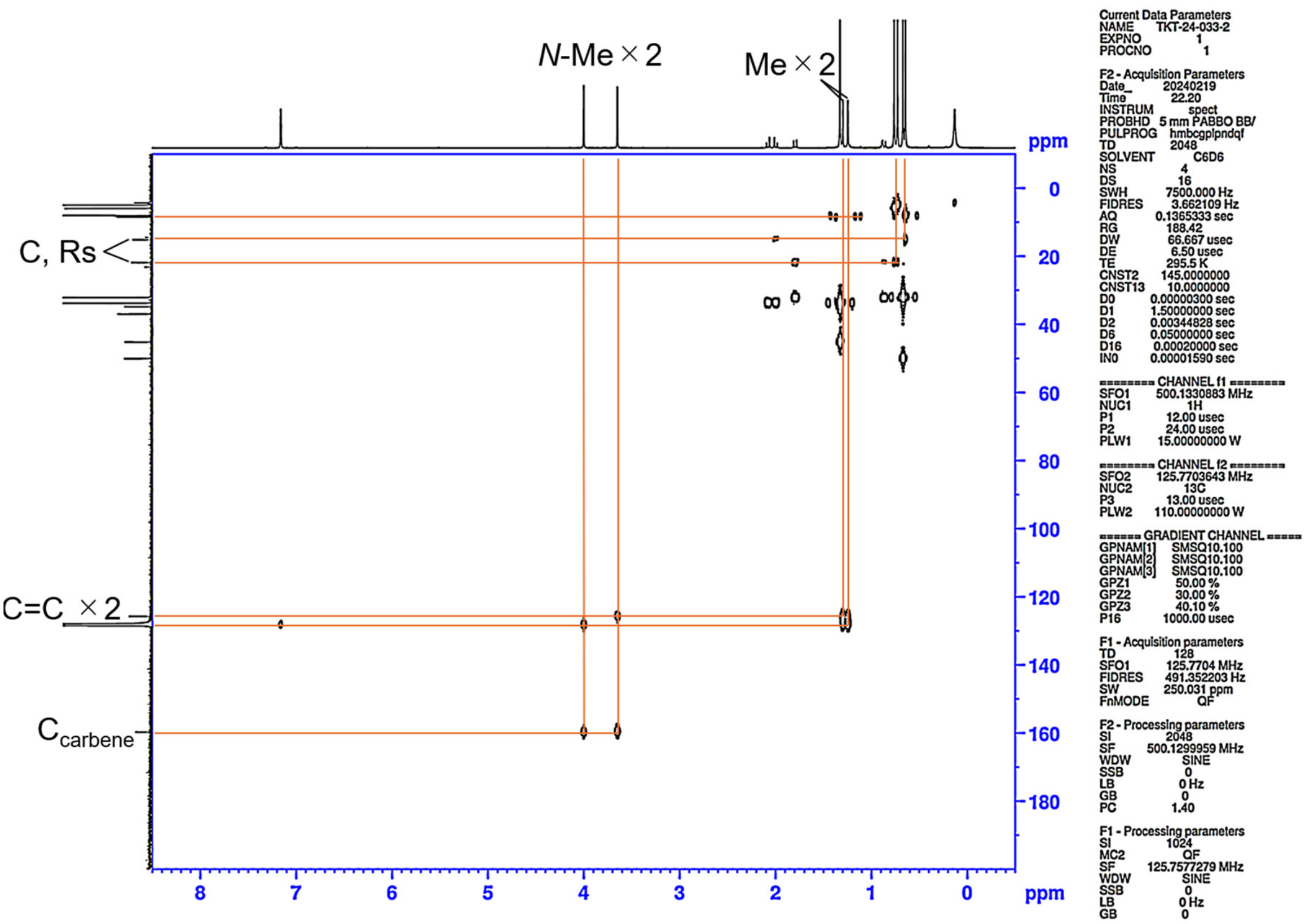

Figure S6. ^{1}H-^{13}C HMBC NMR spectrum of **3** in C_6D_6 at 296 K. Cross-peaks indicate an IMe_4 unit and Rs groups.

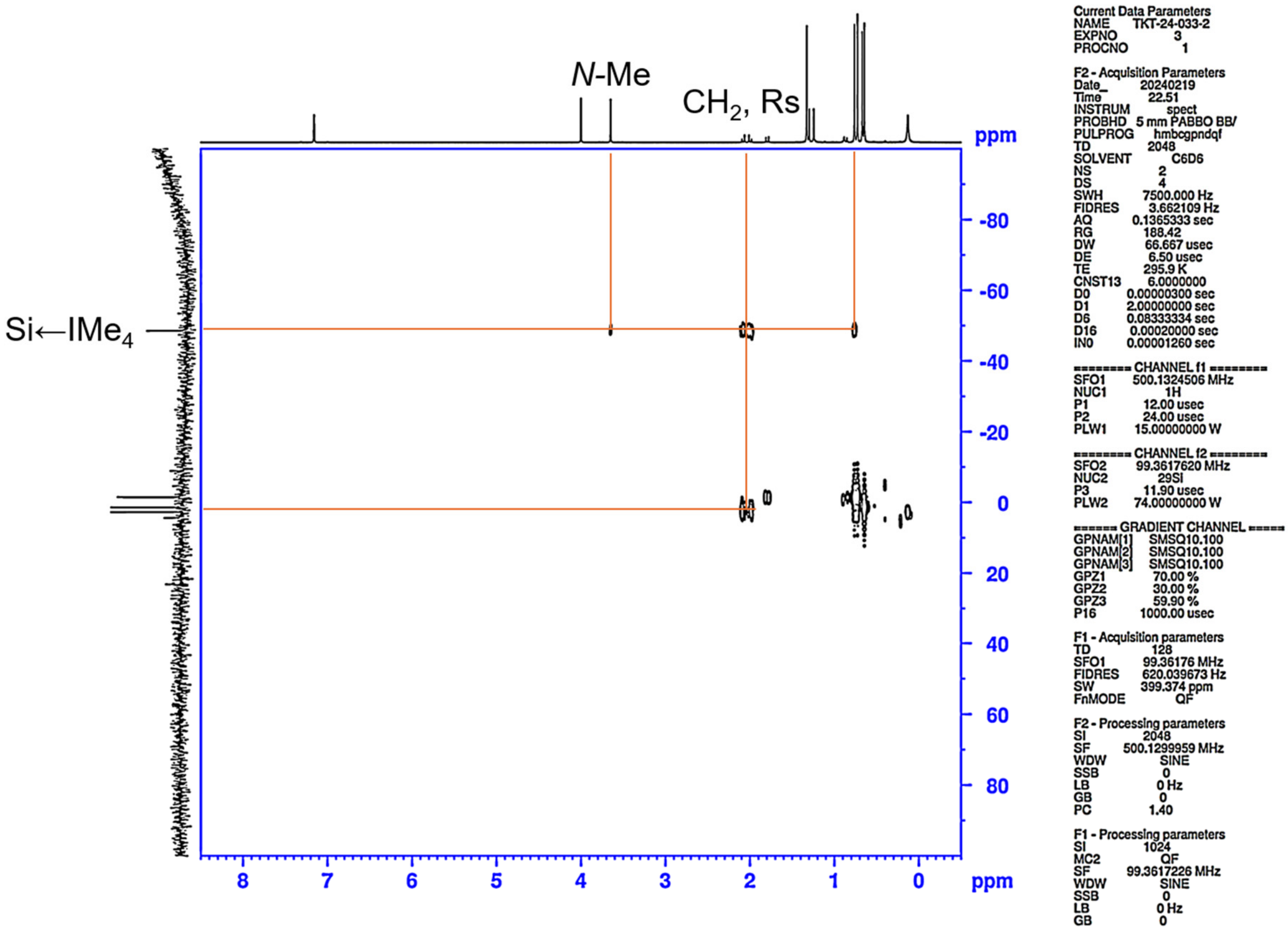

Figure S7. ^{1}H-^{29}Si HMBC NMR spectrum of **3** in C_6D_6 at 296 K. Cross-peaks indicate an IMe_4–Si–Rs linkage.

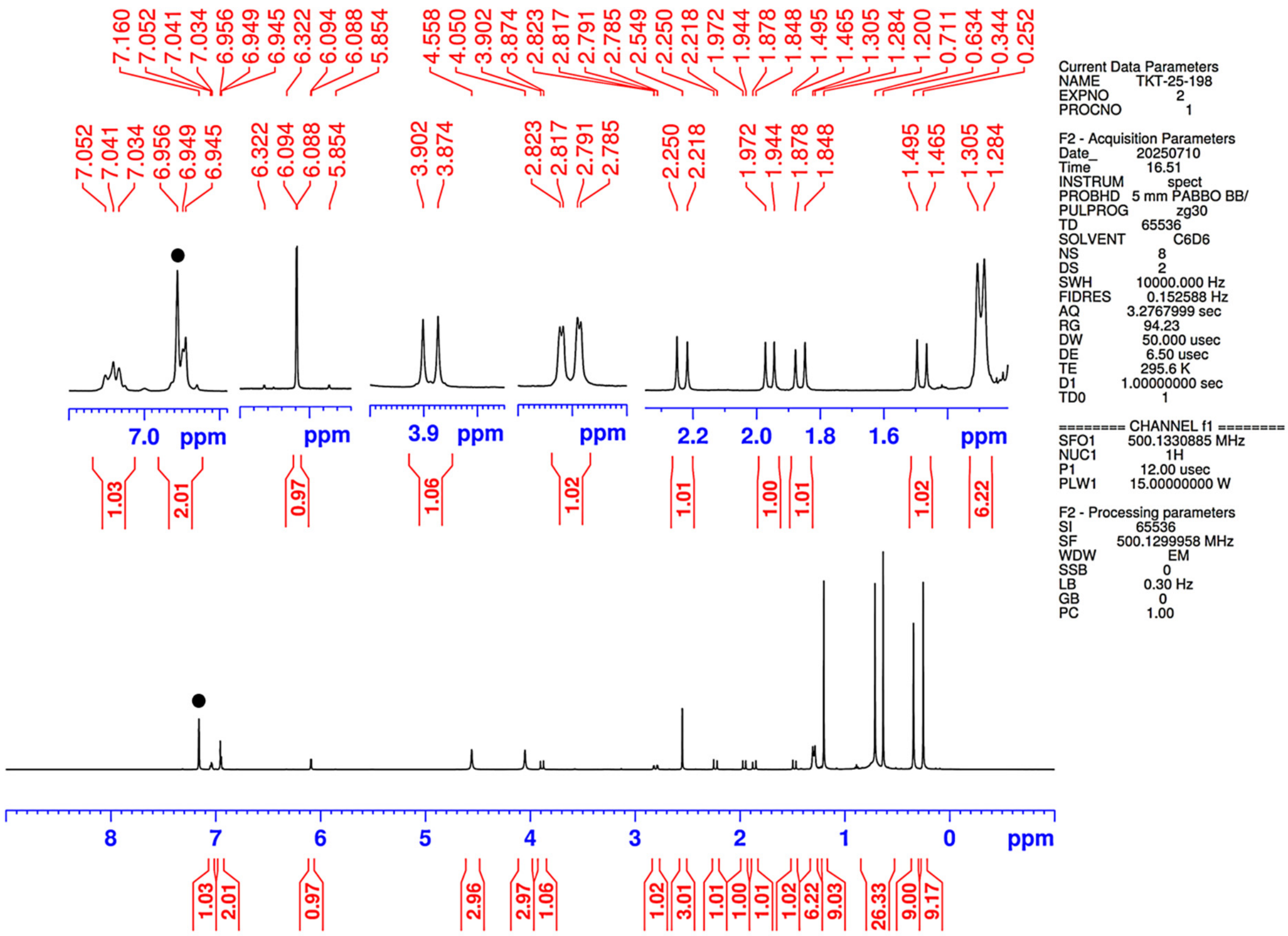

Figure S8. ^{1}H NMR spectrum of **6** in C_6D_6 at 296 K (● = C_6D_5H).

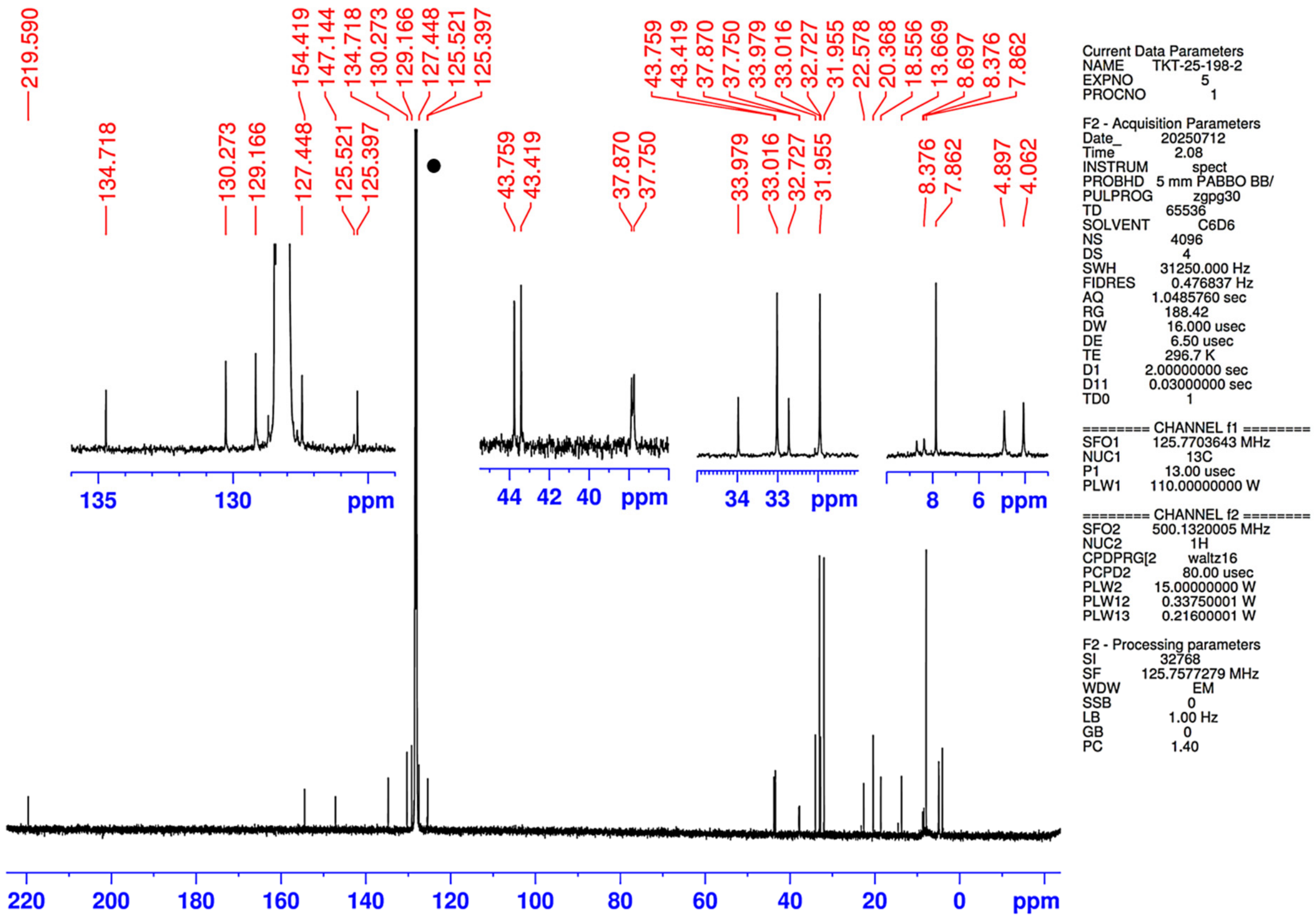

Figure S9. $^{13}C\{^{1}H\}$ CPD NMR spectrum of **6** in C_6D_6 at 297 K (● = C_6D_6).

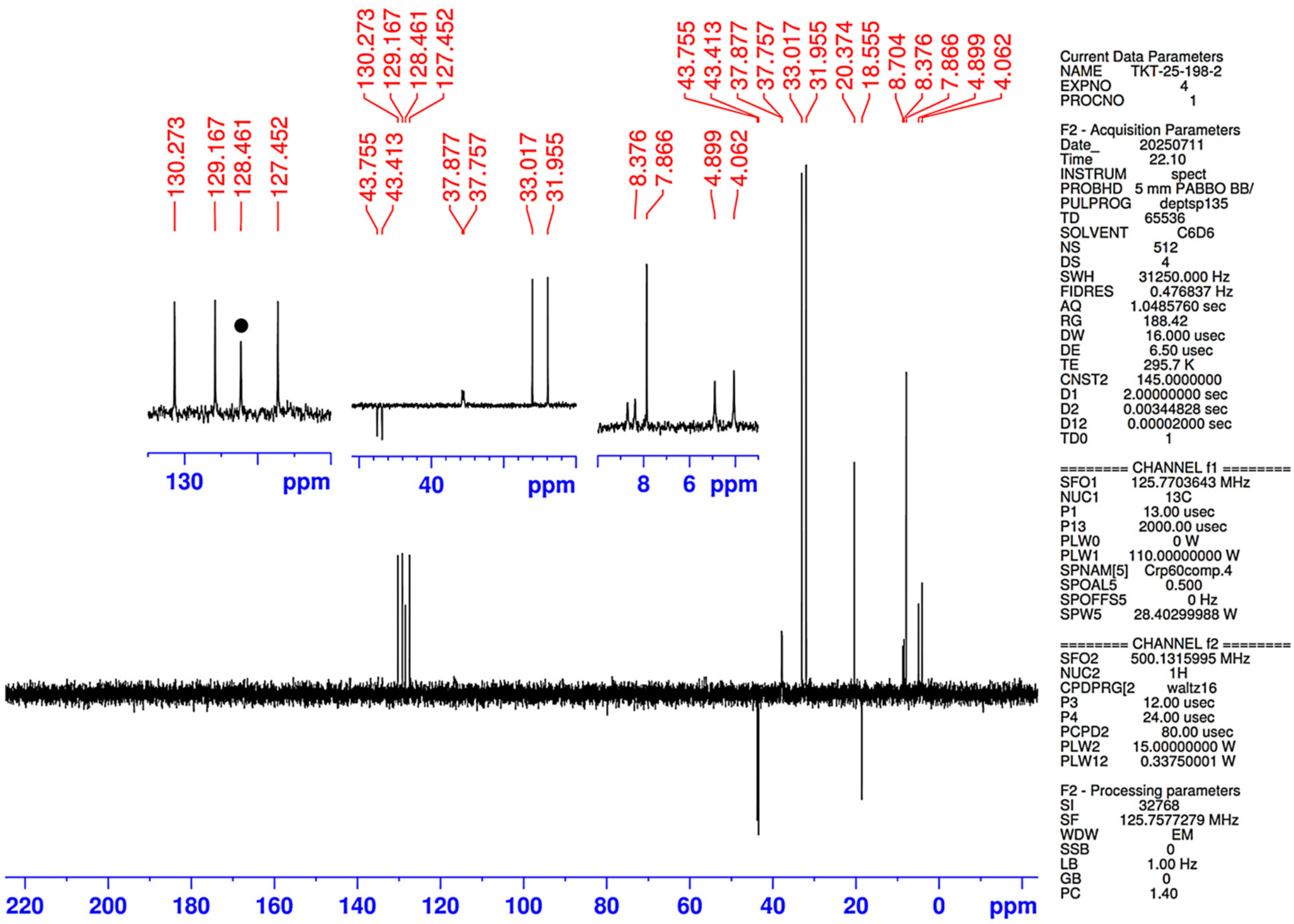

Figure S10. $^{13}C\{^{1}H\}$ DEPT 135 NMR spectrum of **6** in C_6D_6 at 296 K (● = C_6D_5H).

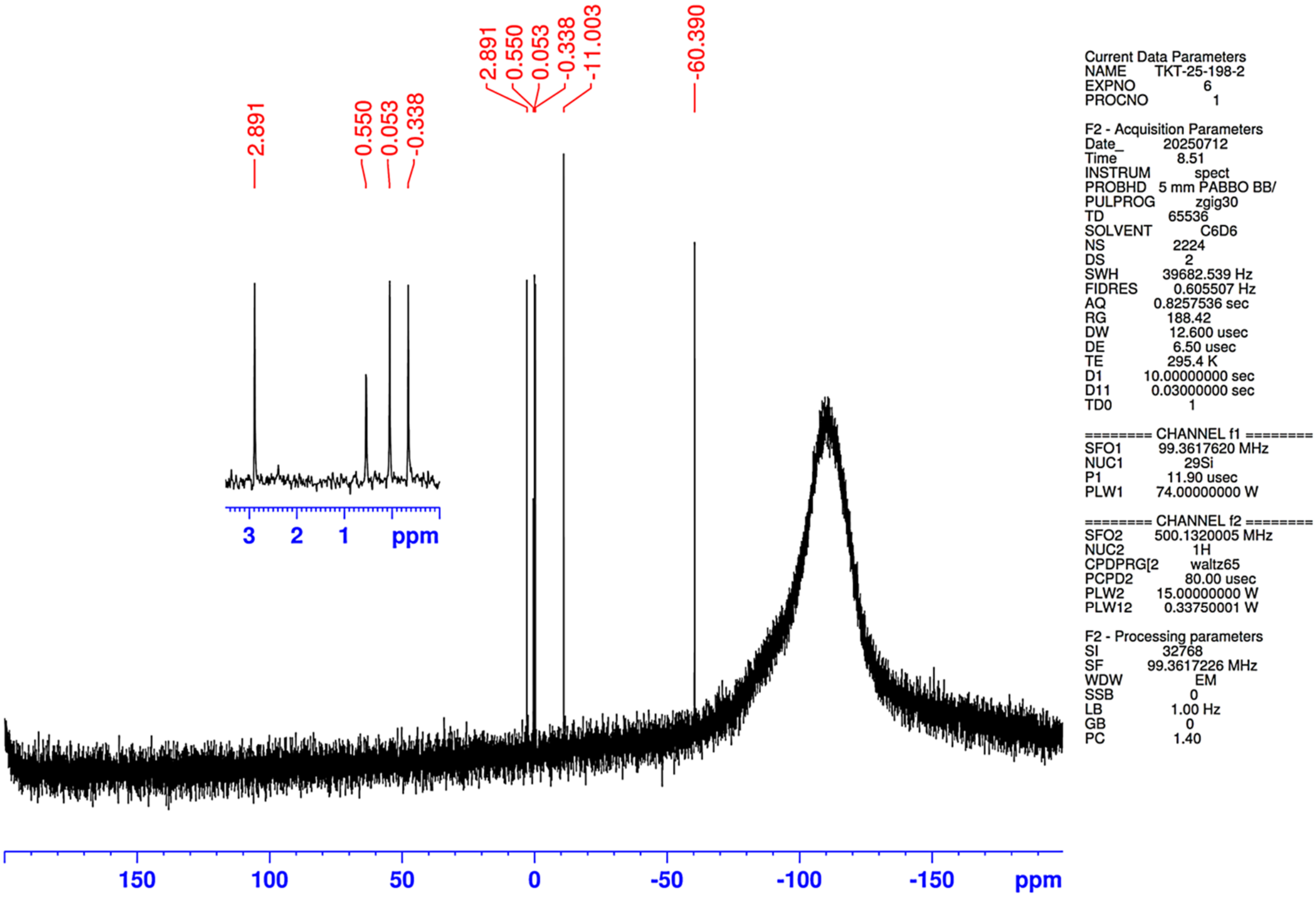

Figure S11. $^{29}Si\{^1H\}$ NMR spectrum of **6** in C_6D_6 at 295 K.

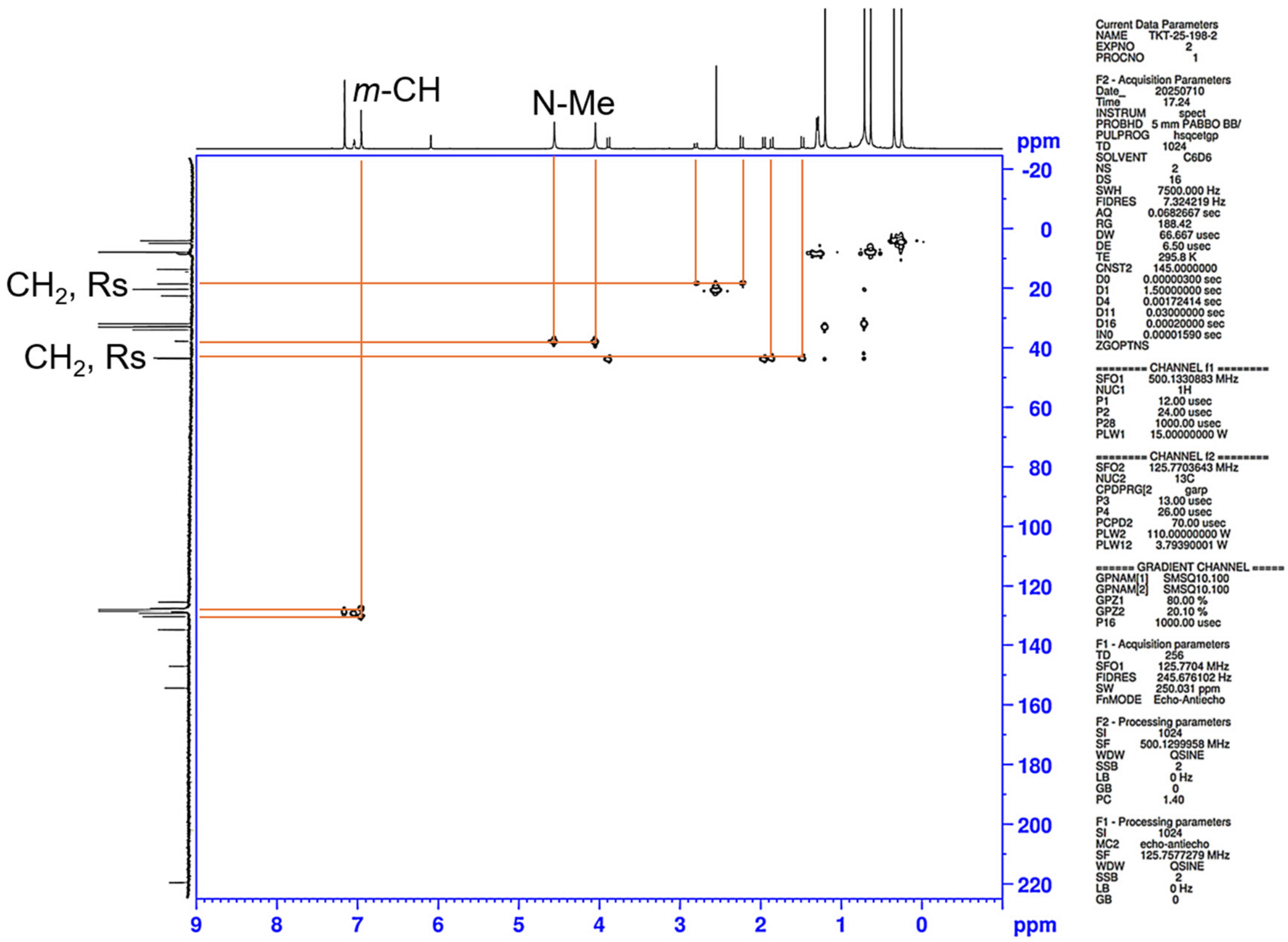

Figure S12. ^1H-^{13}C HSQC NMR spectrum of **6** in C_6D_6 at 296 K. Geminal protons, N-Me, and *m*-CH were confirmed by cross-peaks.

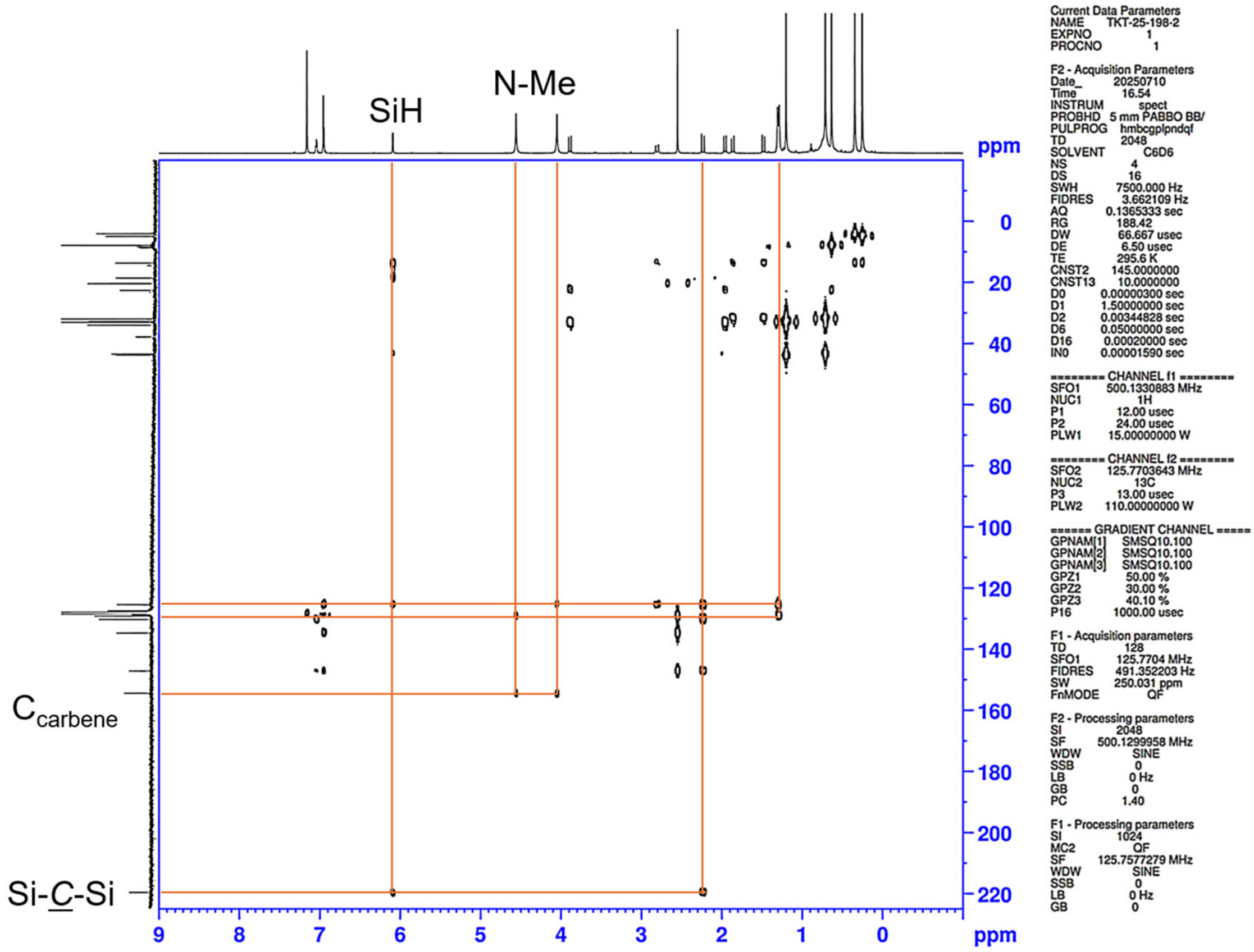

Figure S13. ^{1}H-^{13}C HMBC NMR spectrum of **6** in C_6D_6 at 296 K. Cross-peaks indicate an IMe_4−RsSi−C−SiH−Rs linkage.

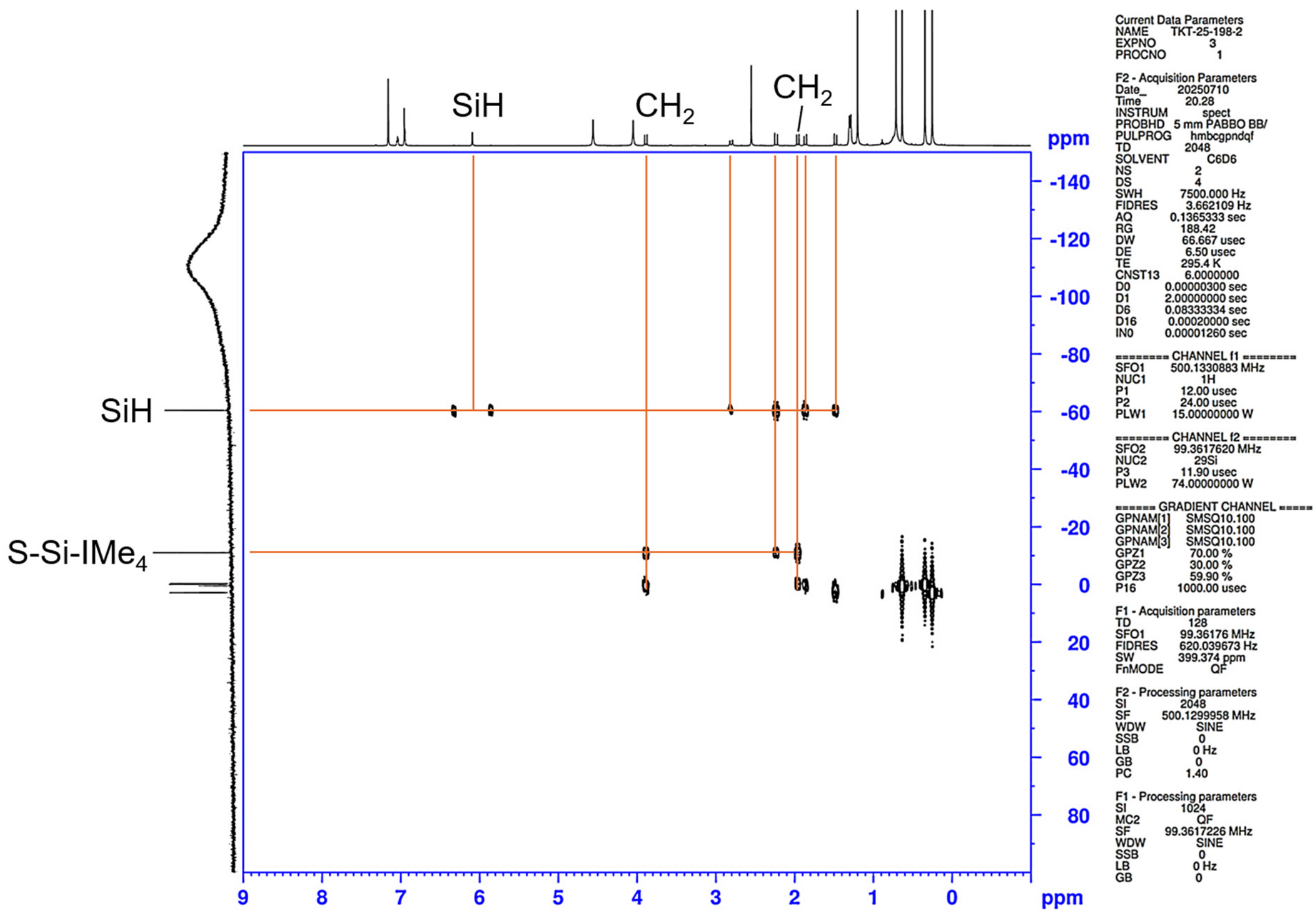

Figure S14. ^{1}H-^{29}Si HMBC NMR spectrum of **6** in C_6D_6 at 295 K. Cross-peaks indicate an Rs−Si−C−SiH−Rs linkage.

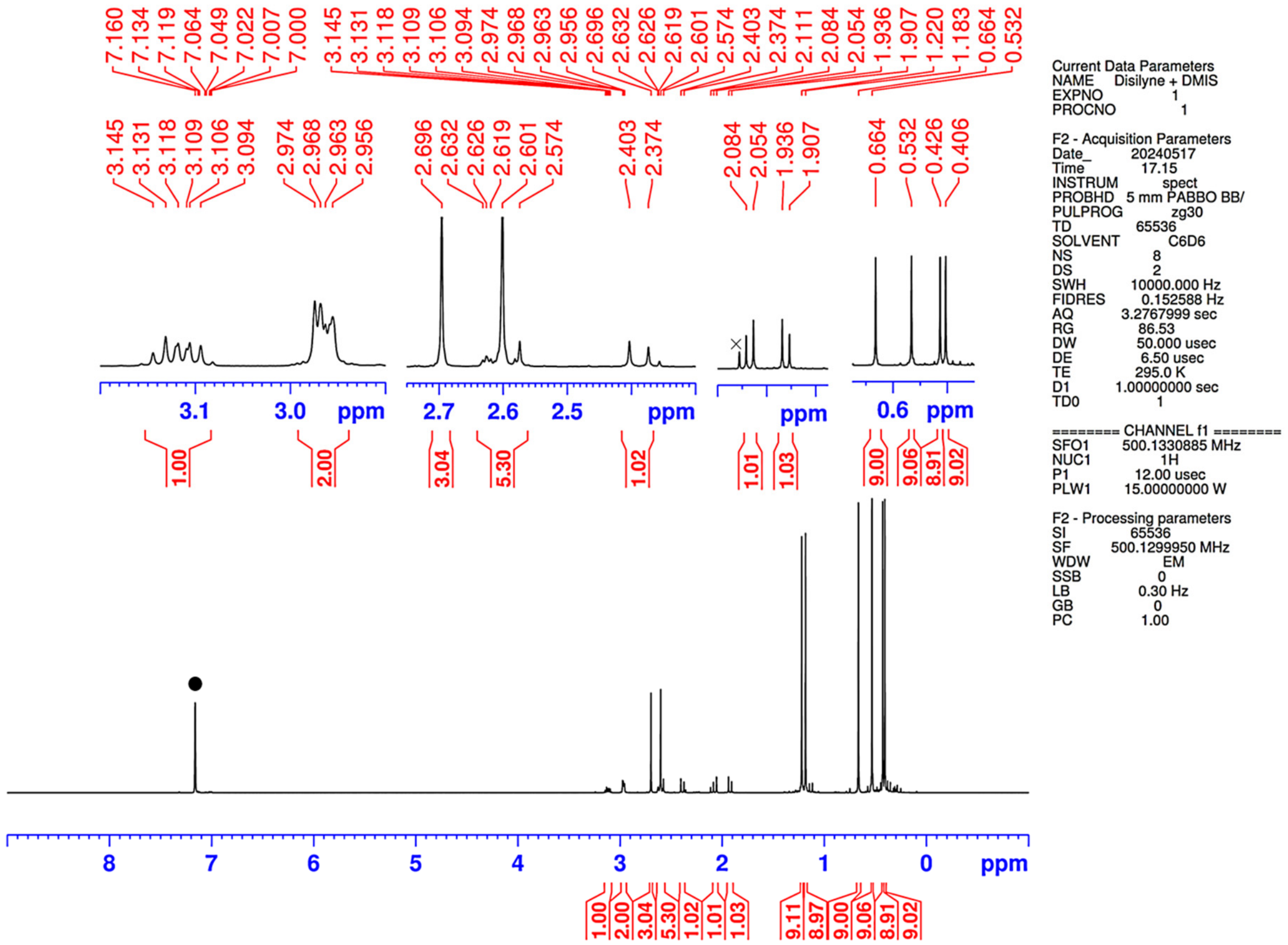

Figure S15. ^{1}H NMR spectrum of **9b** in C_6D_6 at 295 K (● = C_6D_5H, × = toluene).

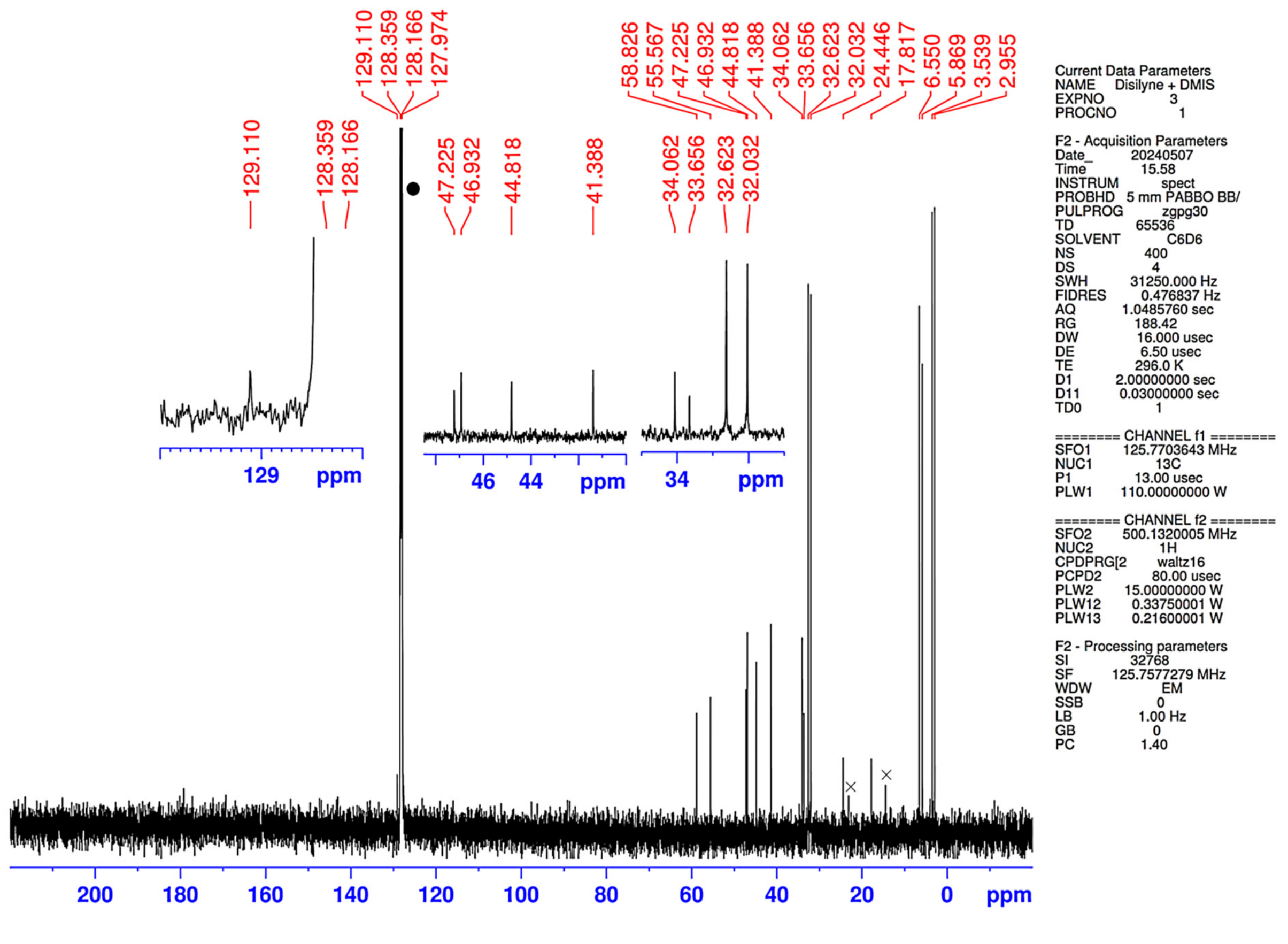

Figure S16. $^{13}C\{^{1}H\}$ CPD NMR spectrum of **9b** in C_6D_6 at 296 K (● = C_6D_6, × = hexane).

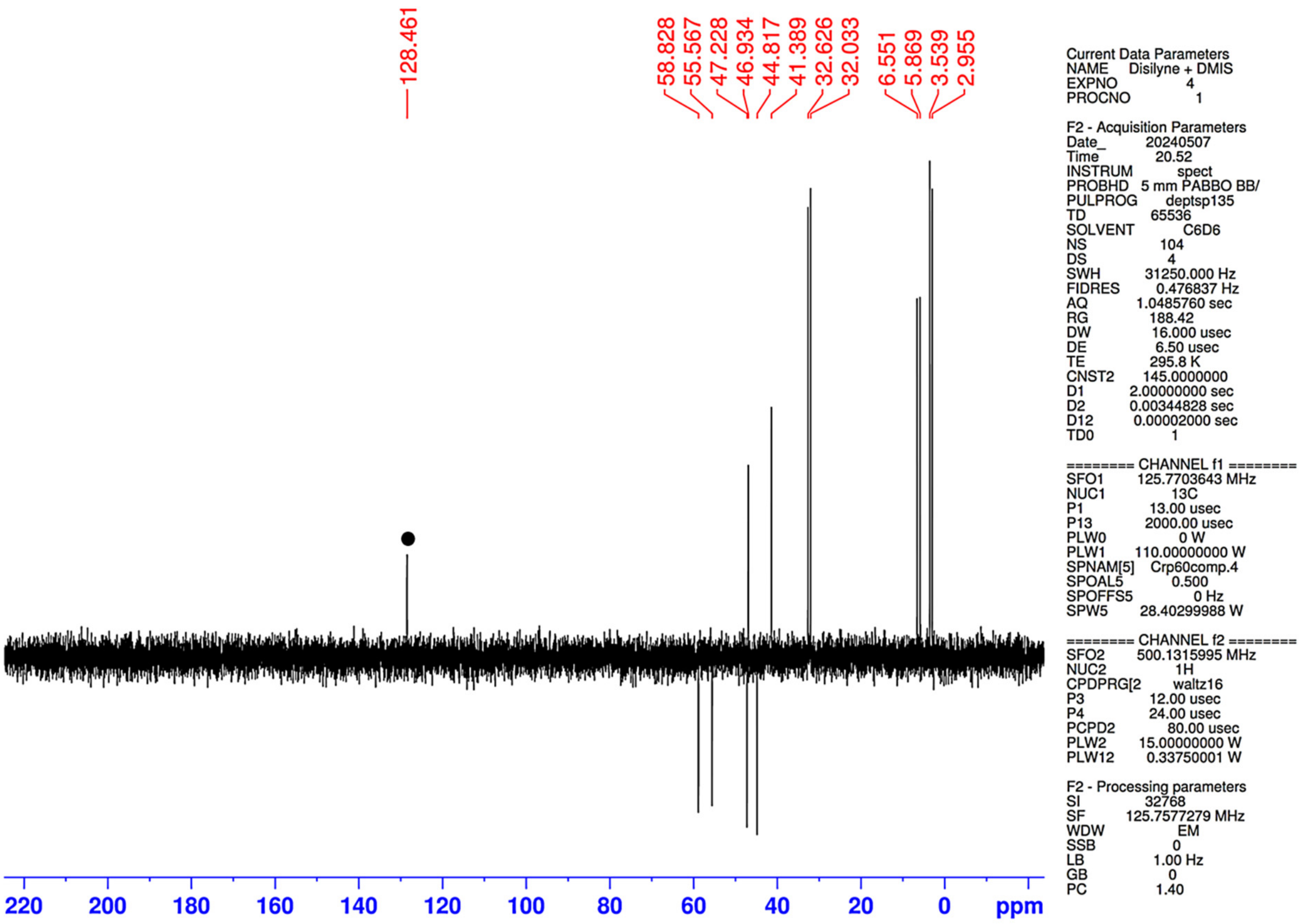

Figure S17. $^{13}C\{^{1}H\}$ DEPT 135 NMR spectrum of **9b** in C_6D_6 at 296 K (● = C_6D_5H).

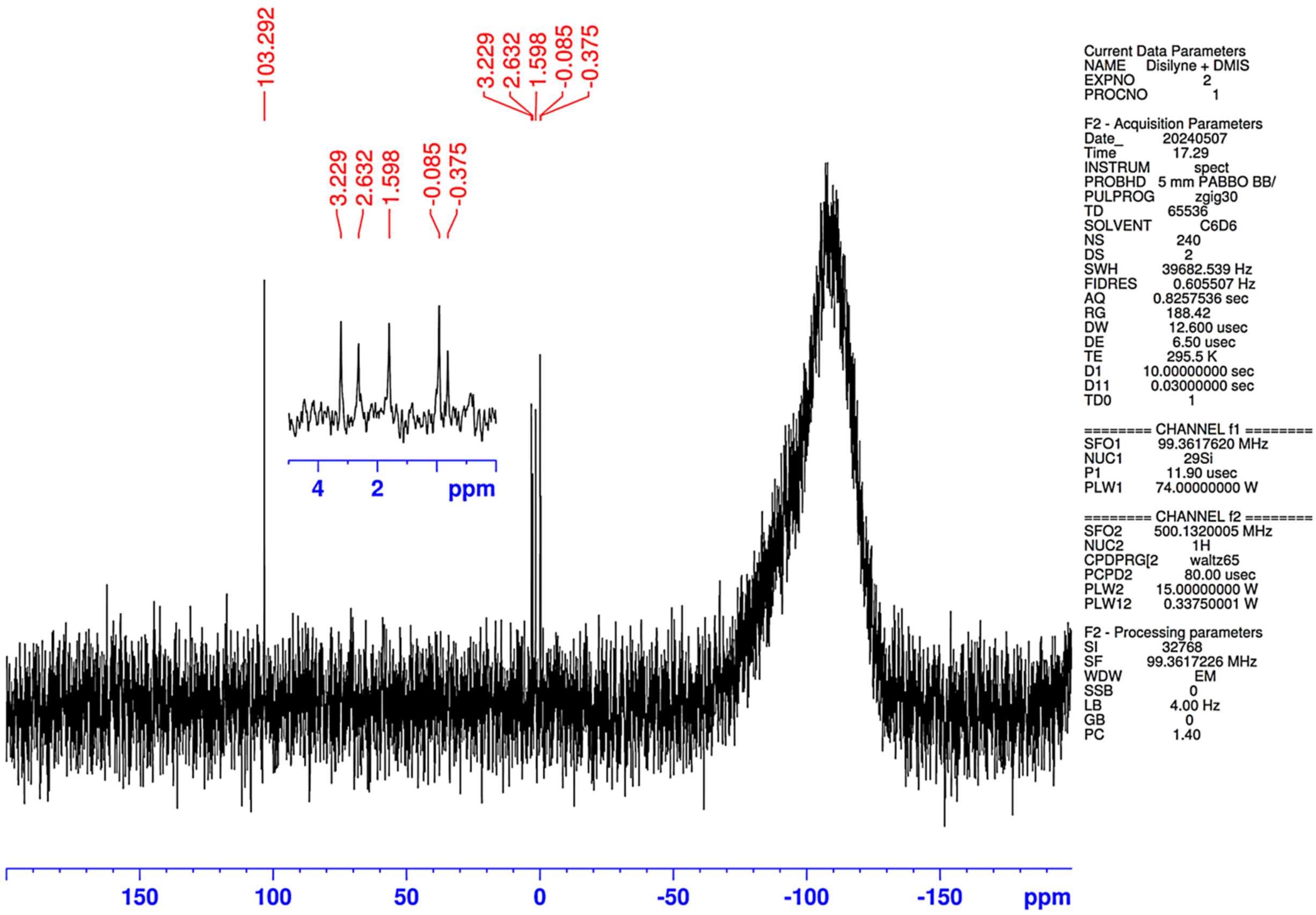

Figure S18. $^{29}Si\{^{1}H\}$ NMR spectrum of **9b** in C_6D_6 at 296 K.

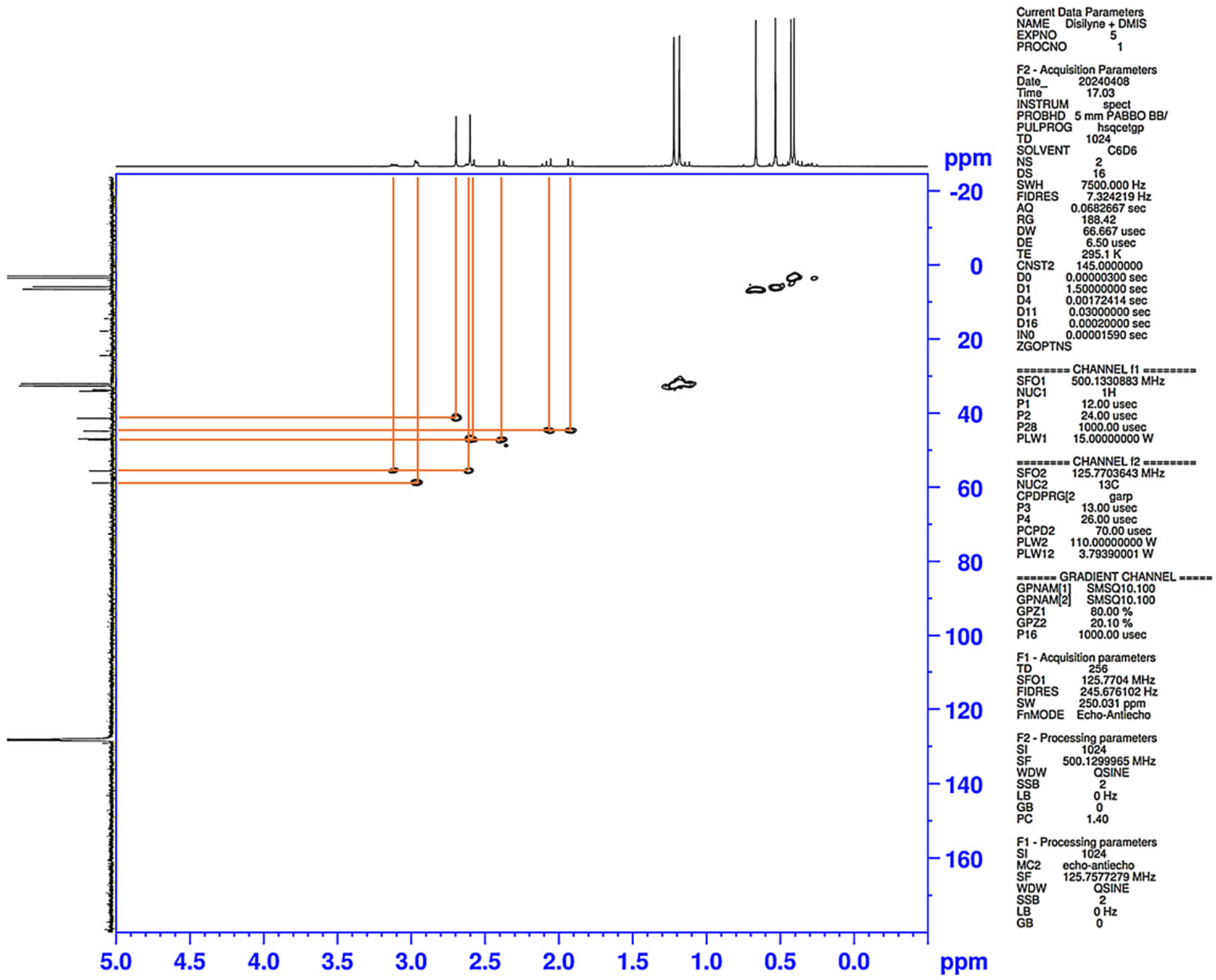

Figure S19. ^{1}H-^{13}C HSQC NMR spectrum of **9b** in C_6D_6 at 295 K. Geminal protons were confirmed by cross-peaks.

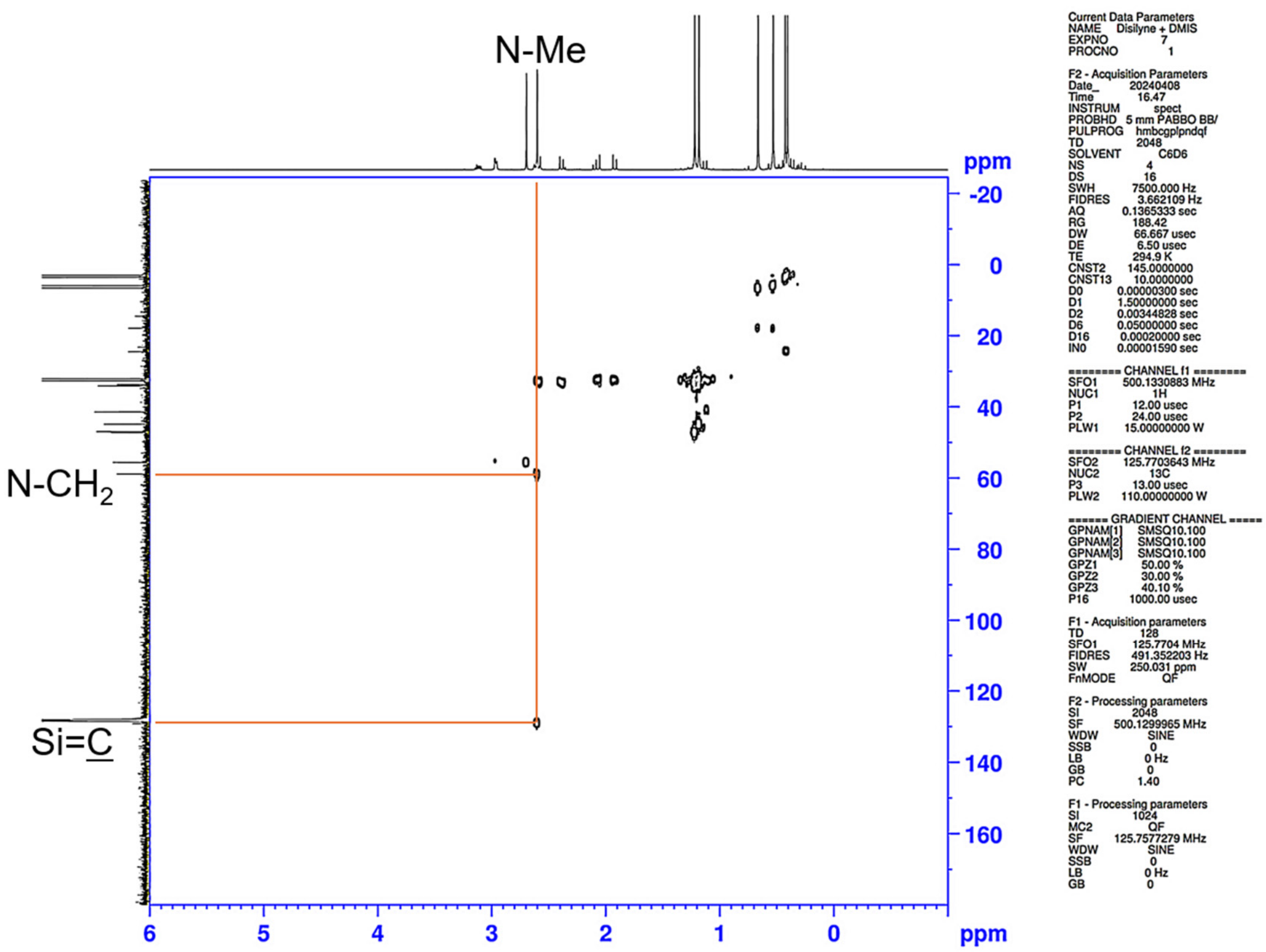

Figure S20. ^{1}H-^{13}C HMBC NMR spectrum of **9b** in C_6D_6 at 295 K. Cross-peaks indicate a $CH_2MeN{-}C{=}Si$ linkage.

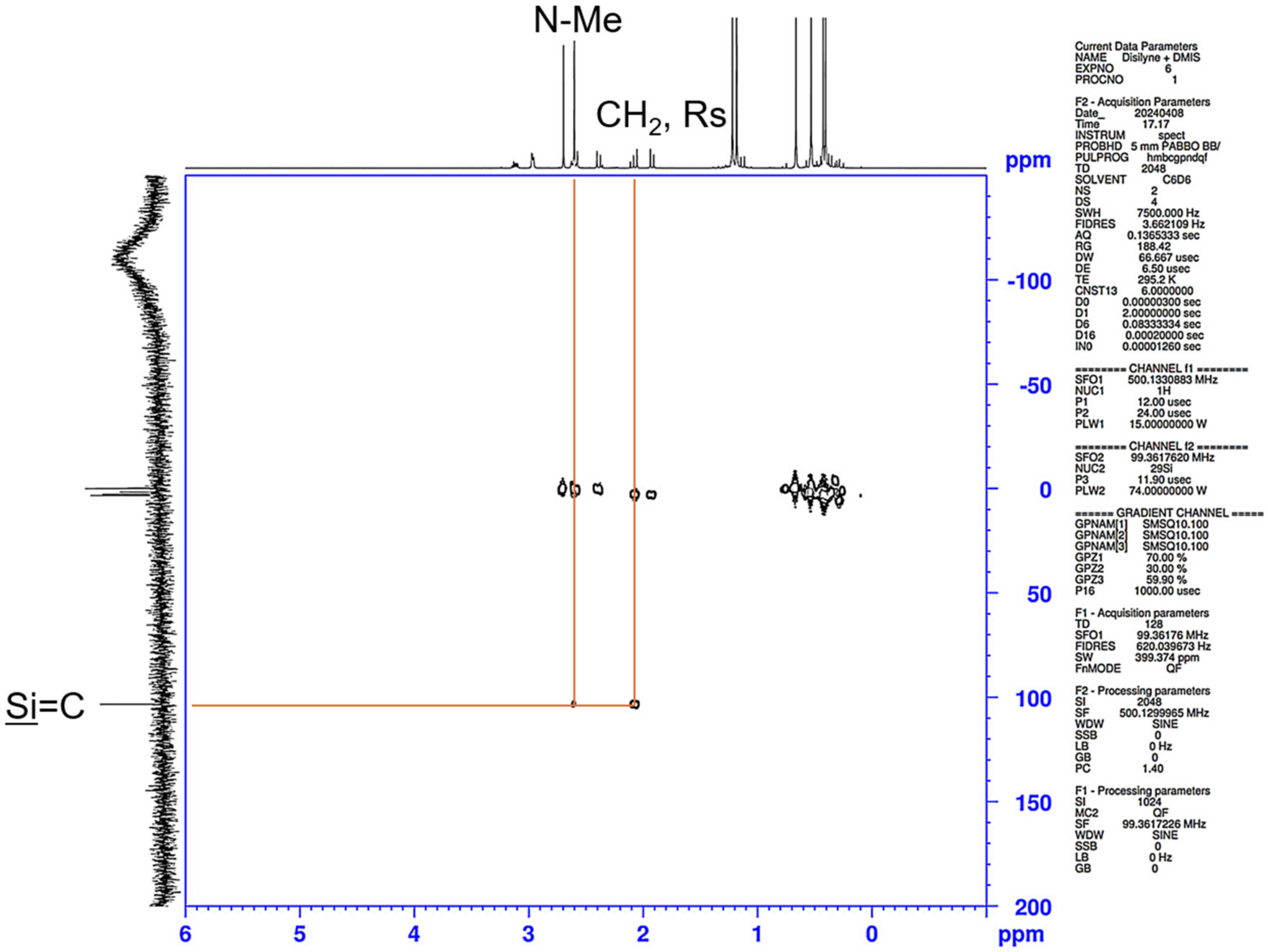

Figure S21. ^{1}H-^{29}Si HMBC NMR spectrum of **9b** in C_6D_6 at 295 K. Cross-peaks indicate a MeN−C=Si−Rs linkage.

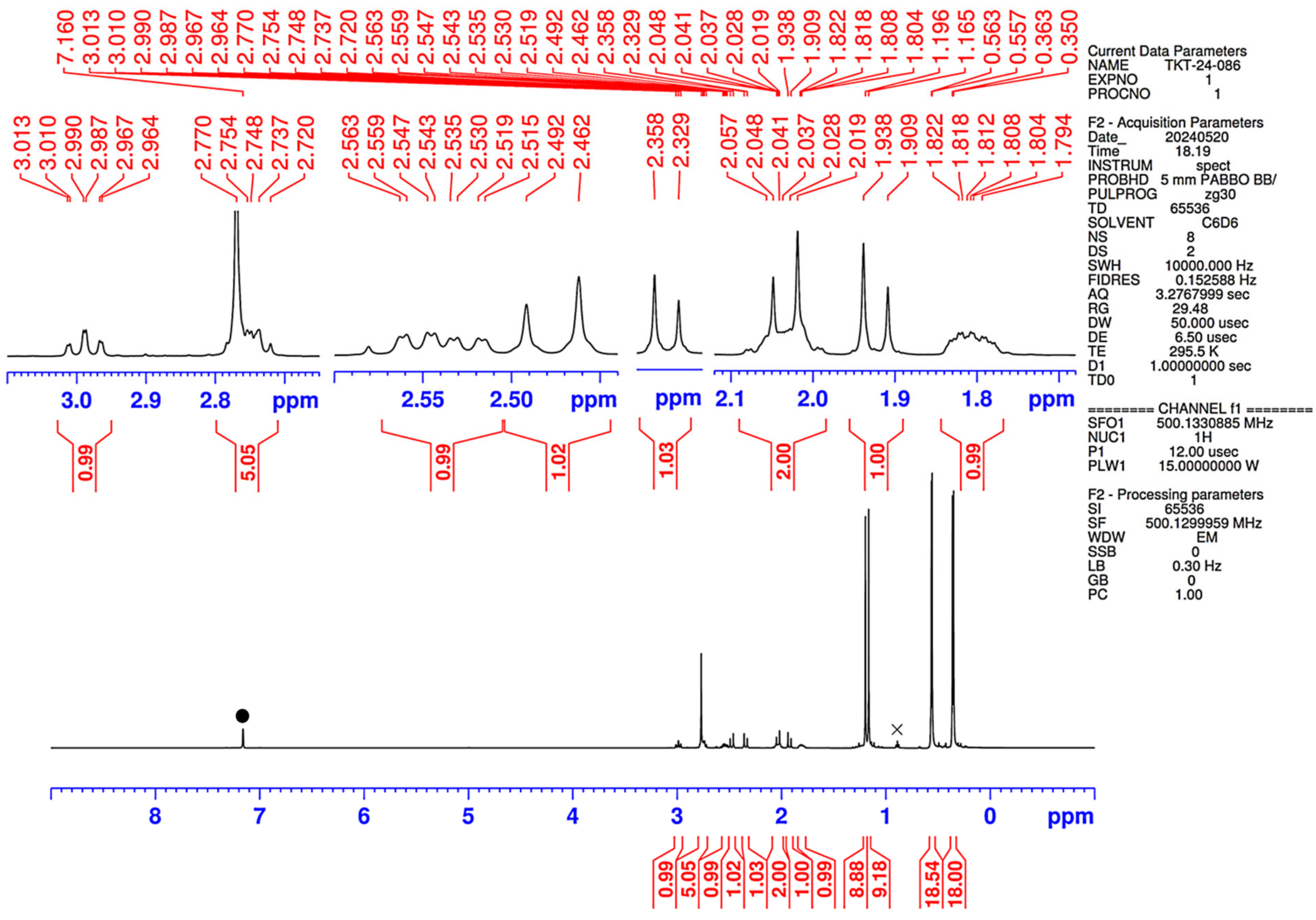

Figure S22. ^{1}H NMR spectrum of **9c** in C_6D_6 at 296 K (● = C_6D_5H, × = hexane).

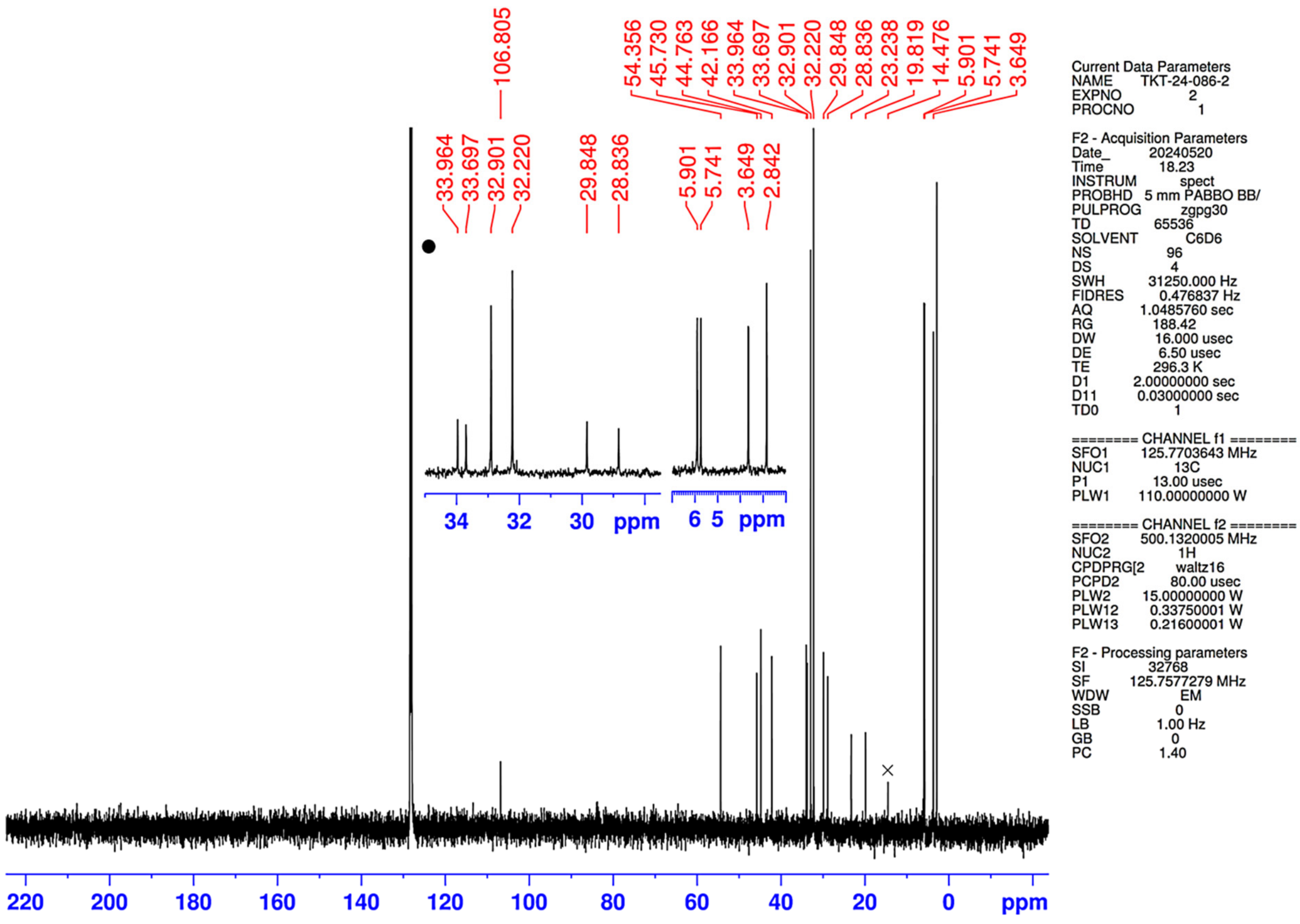

Figure S23. $^{13}C\{^1H\}$ CPD NMR spectrum of **9c** in C_6D_6 at 296 K (● = C_6D_6, × = hexane).

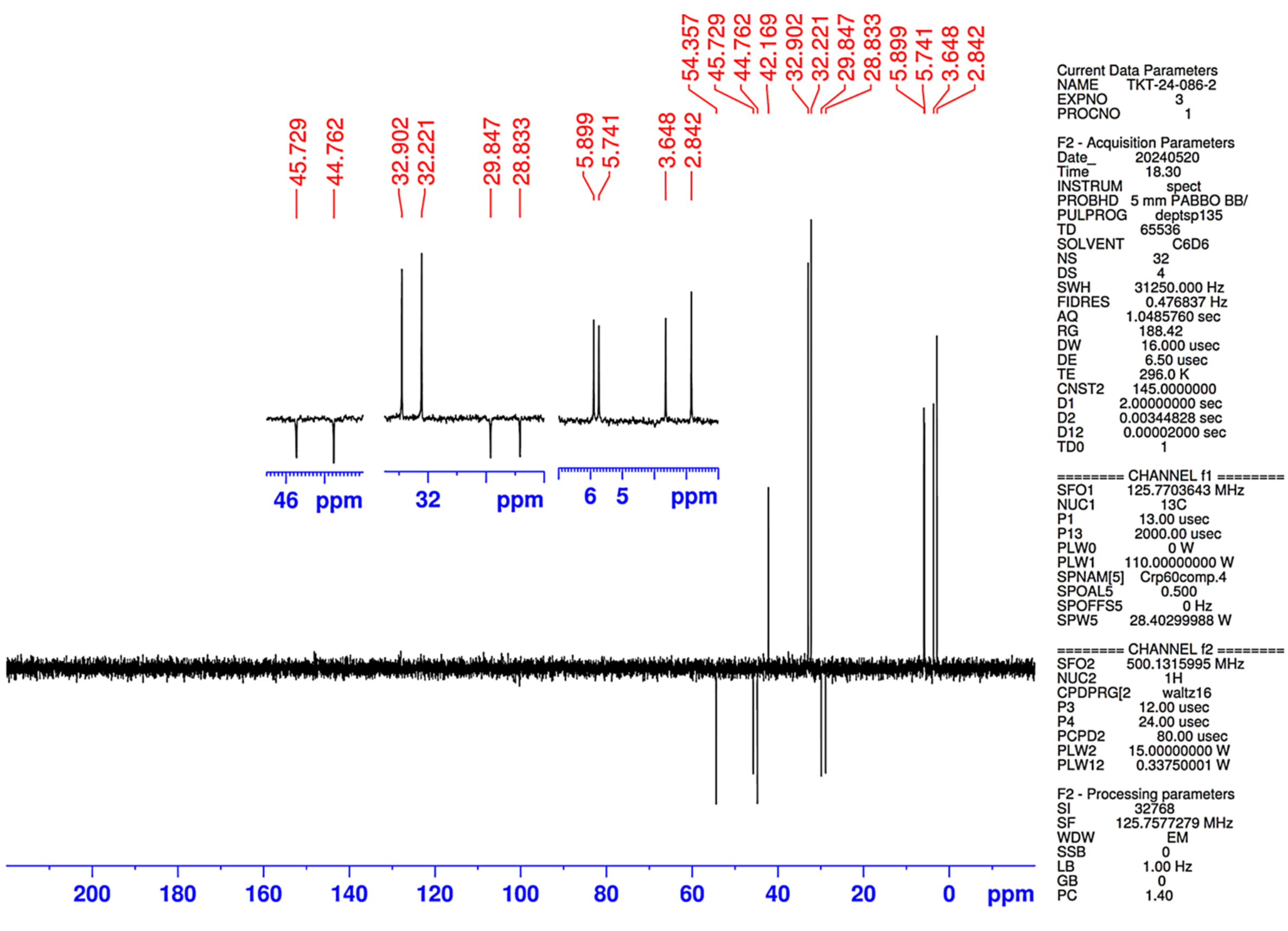

Figure S24. $^{13}C\{^1H\}$ DEPT 135 NMR spectrum of **9c** in C_6D_6 at 296 K.

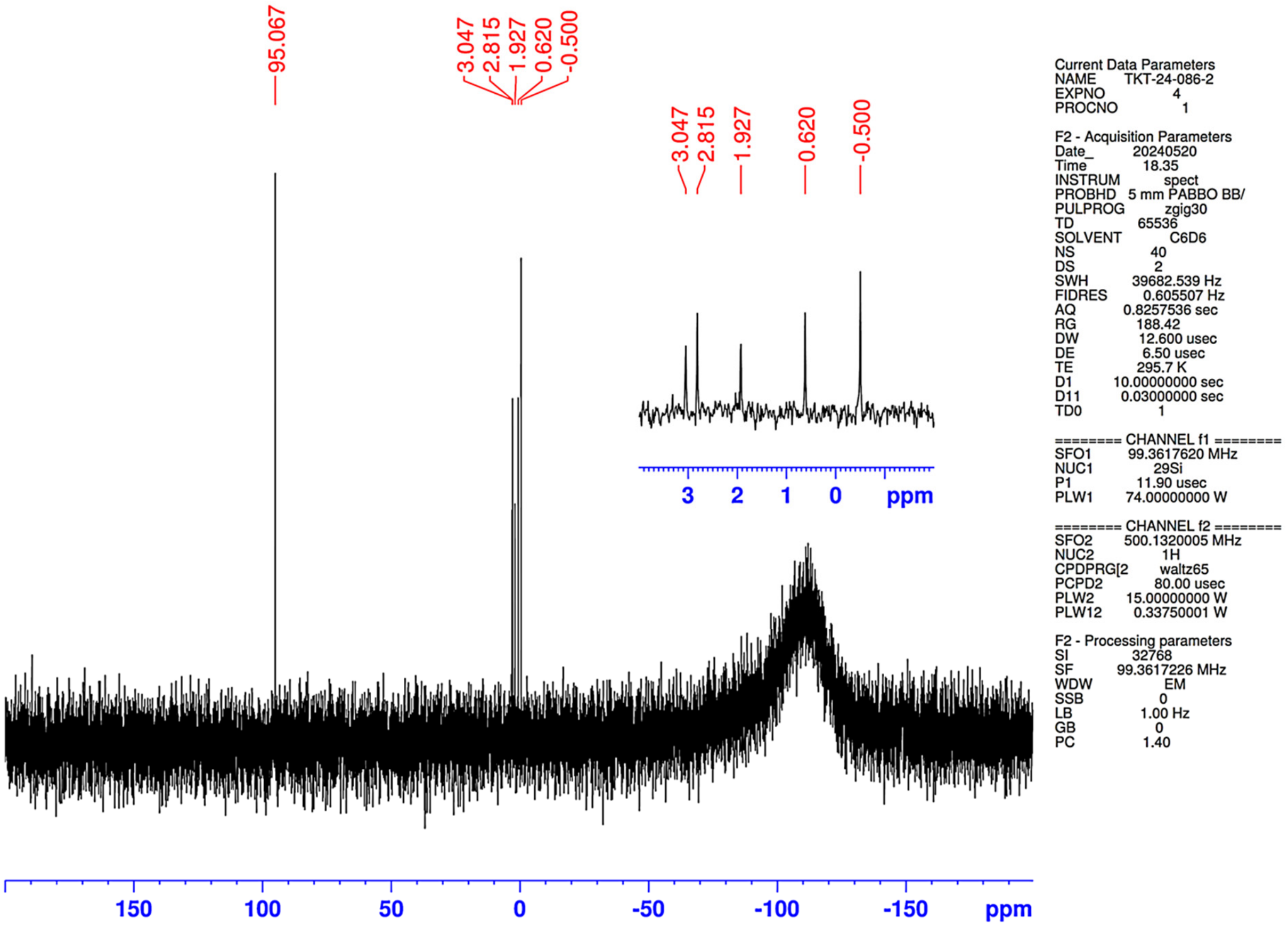

Figure S25. $^{29}Si\{^1H\}$ NMR spectrum of **9c** in C_6D_6 at 296 K.

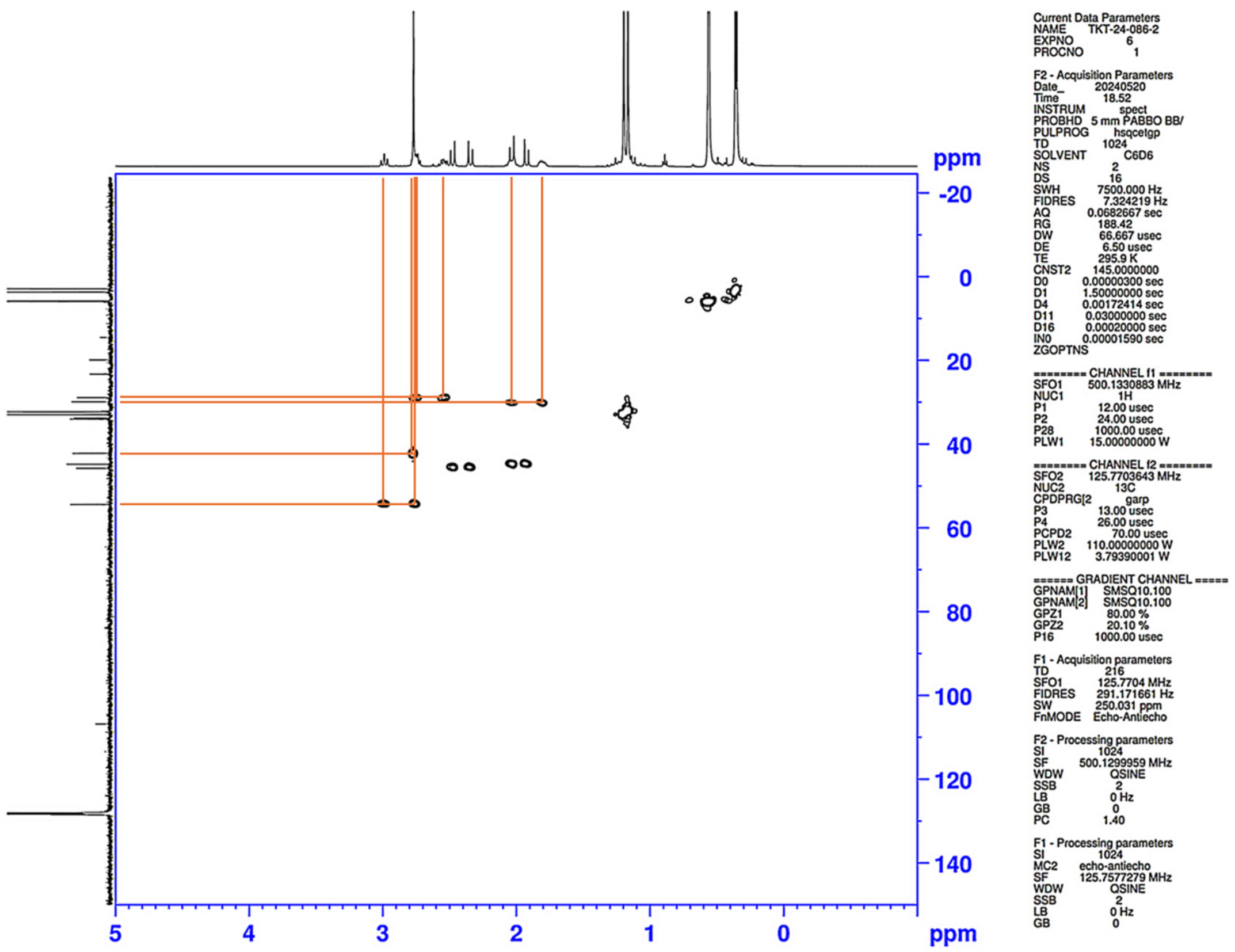

Figure S26. ^1H-^{13}C HSQC NMR spectrum of **9c** in C_6D_6 at 296 K. Geminal protons were confirmed by cross-peaks.

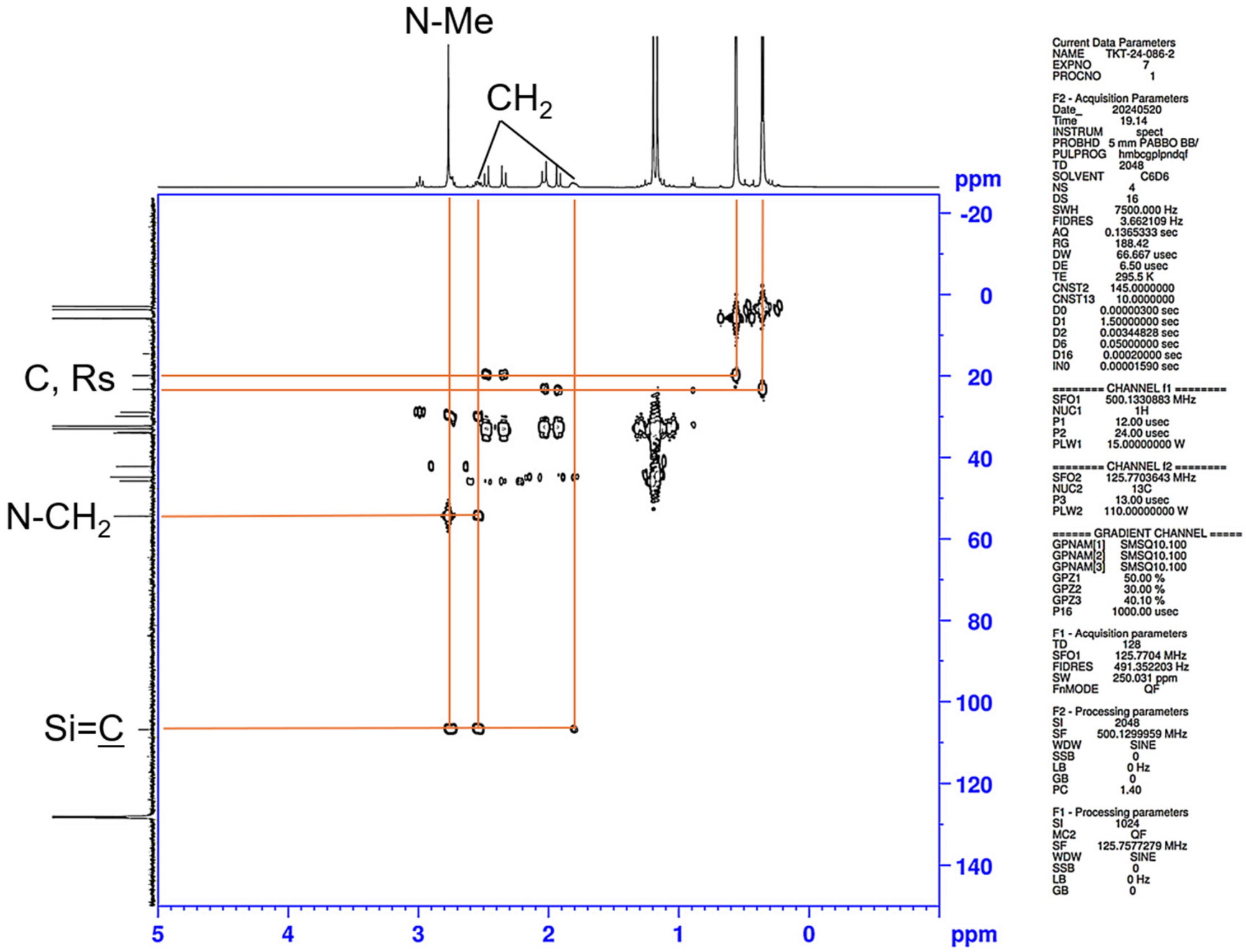

Figure S27. ^{1}H-^{13}C HMBC NMR spectrum of **9c** in C_6D_6 at 296 K. Cross-peaks indicate a CH_2−C−Si−NMe linkage and Rs groups.

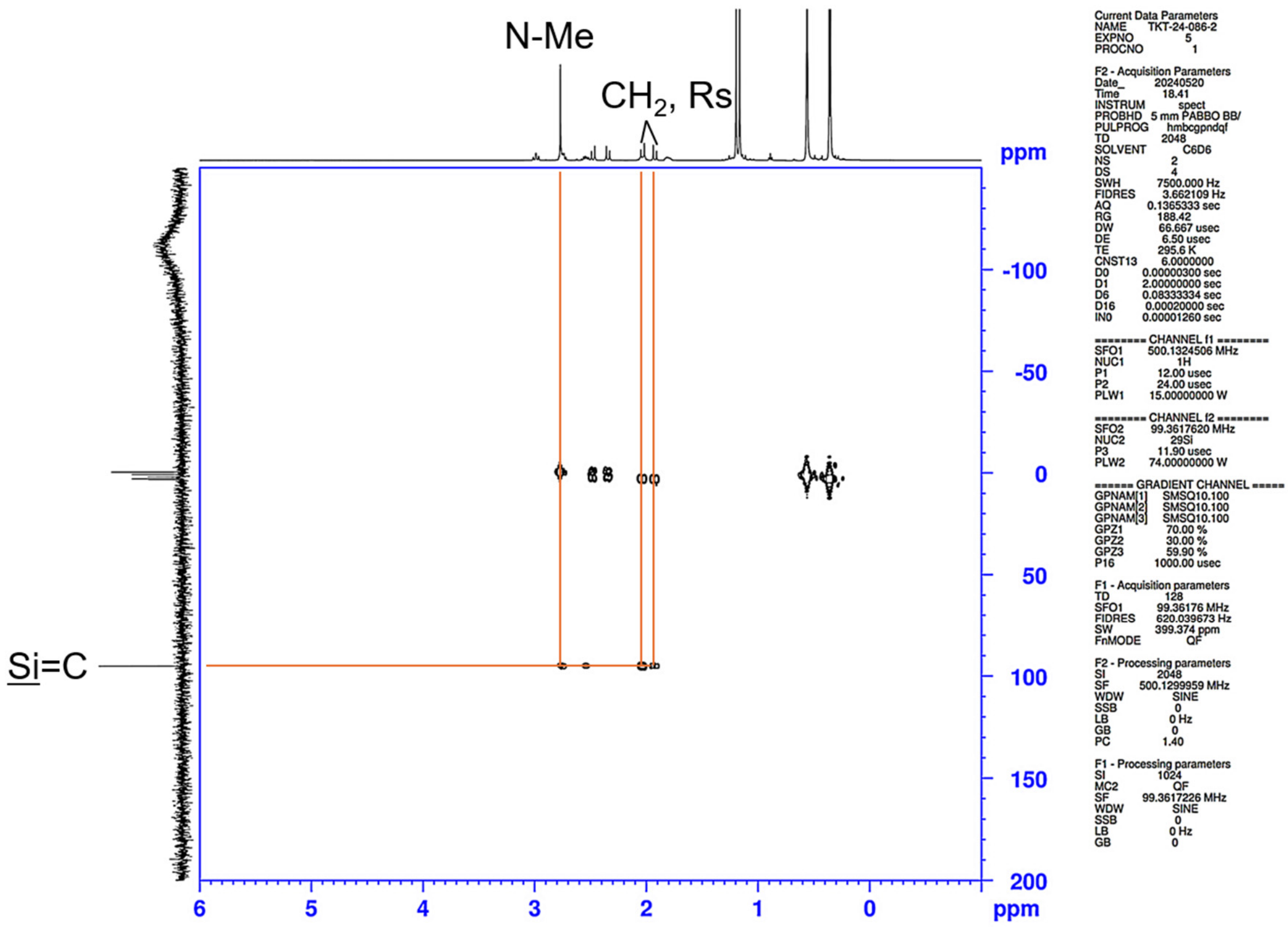

Figure S28. ^{1}H-^{29}Si HMBC NMR spectrum of **9c** in C_6D_6 at 296 K. Cross-peaks indicate a MeN−Si−C=Si−Rs linkage.

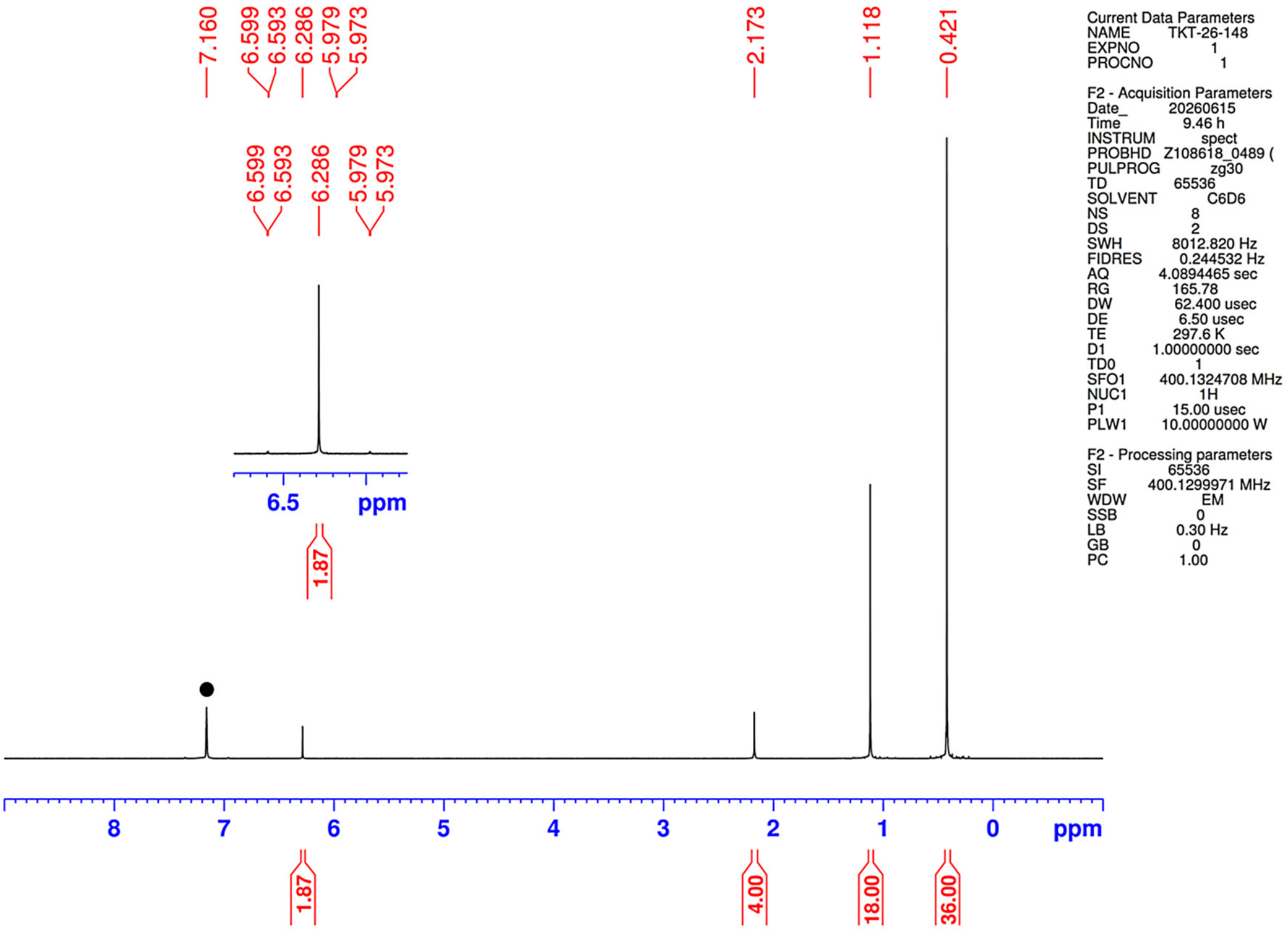

Figure S29. ^{1}H NMR spectrum of **12** in C_6D_6 at 298 K (● = C_6D_5H).

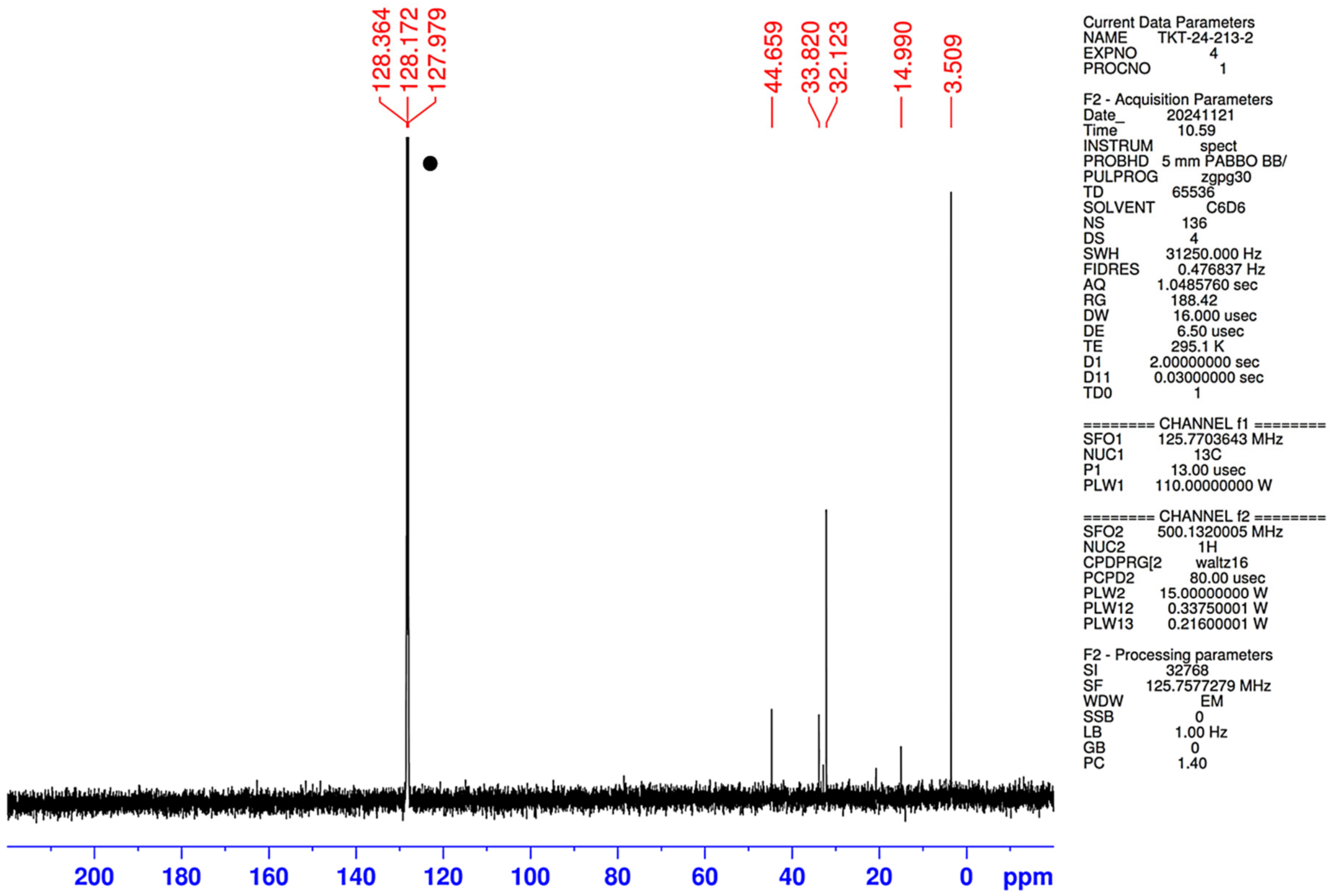

Figure S30. $^{13}C\{^{1}H\}$ CPD NMR spectrum of **12** in C_6D_6 at 295 K (● = C_6D_6).

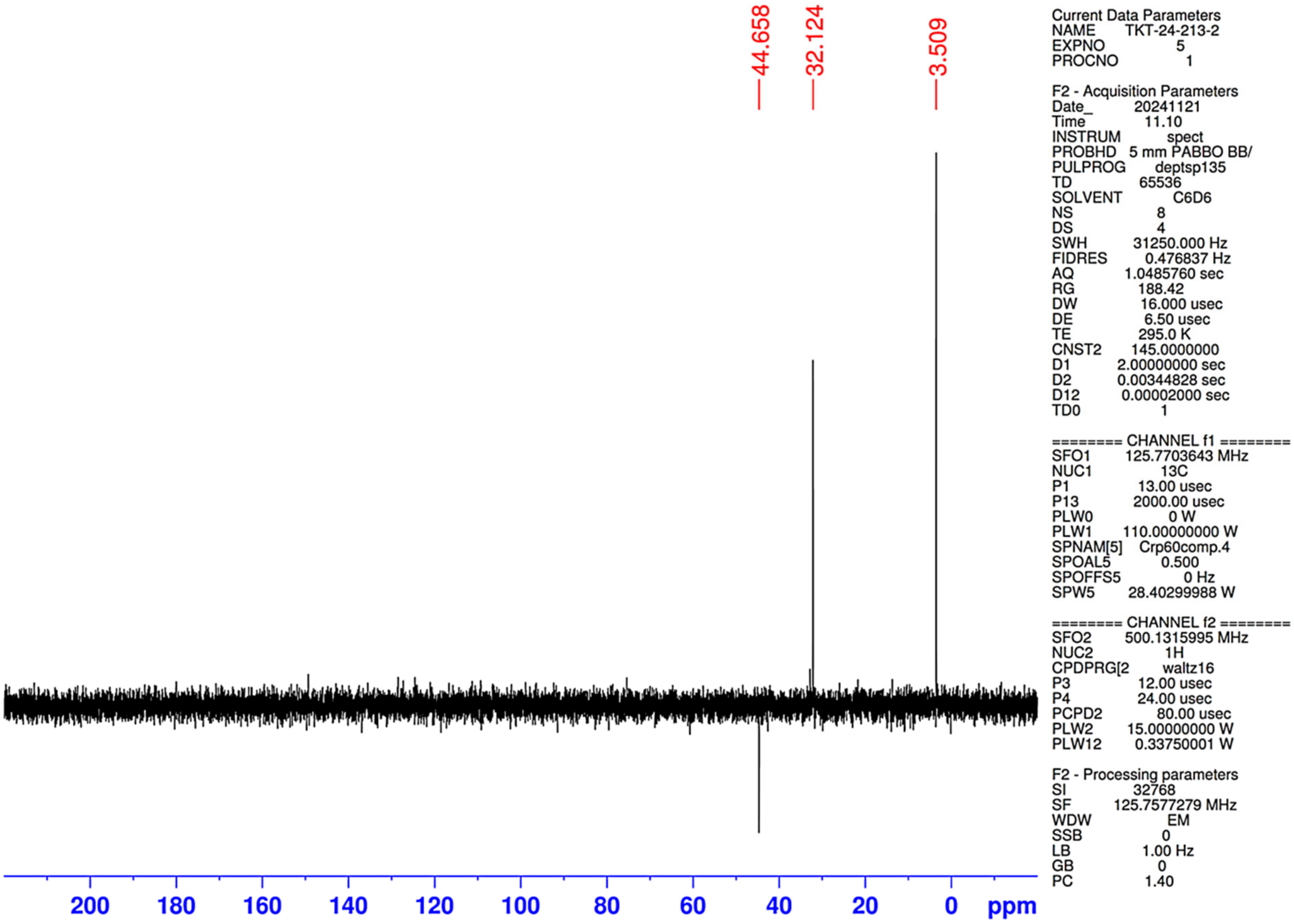

Figure S31. $^{13}C\{^1H\}$ DEPT 135 NMR spectrum of **12** in C_6D_6 at 295 K.

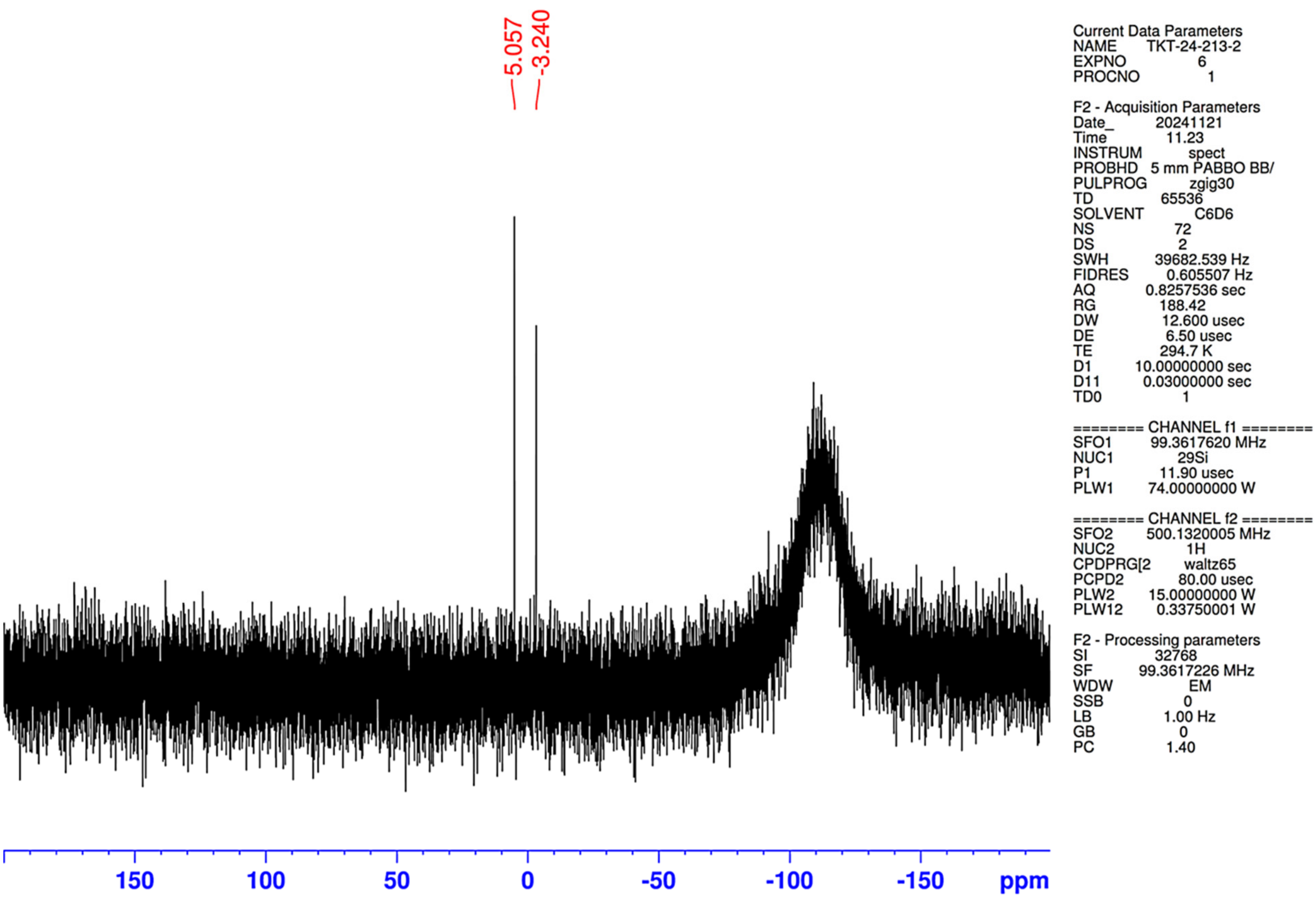

Figure S32. $^{29}Si\{^1H\}$ NMR spectrum of **12** in C_6D_6 at 295 K.

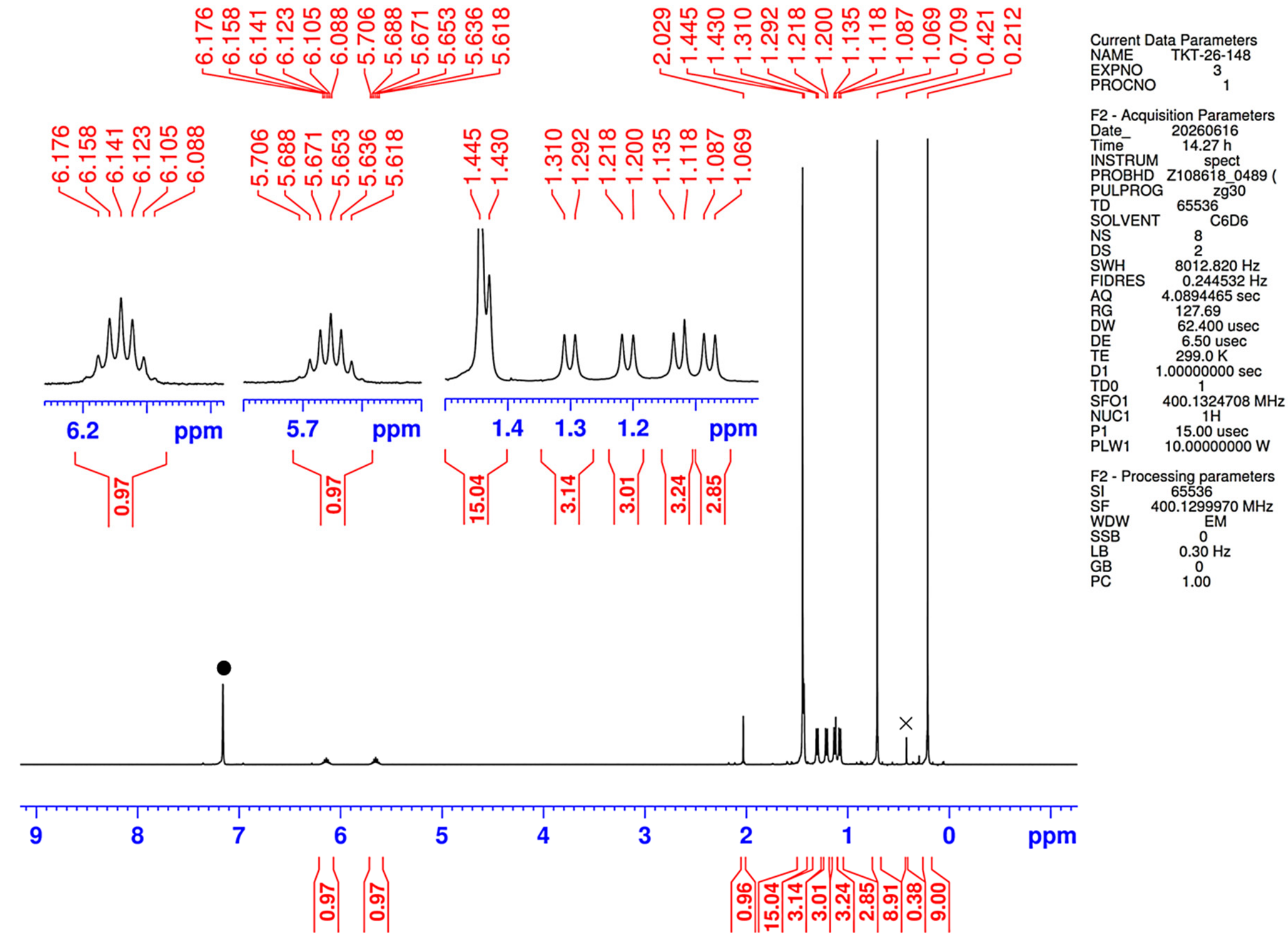

Figure S33. ^{1}H NMR spectrum of **13** in C_6D_6 at 299 K (● = C_6D_5H, × = **12**).

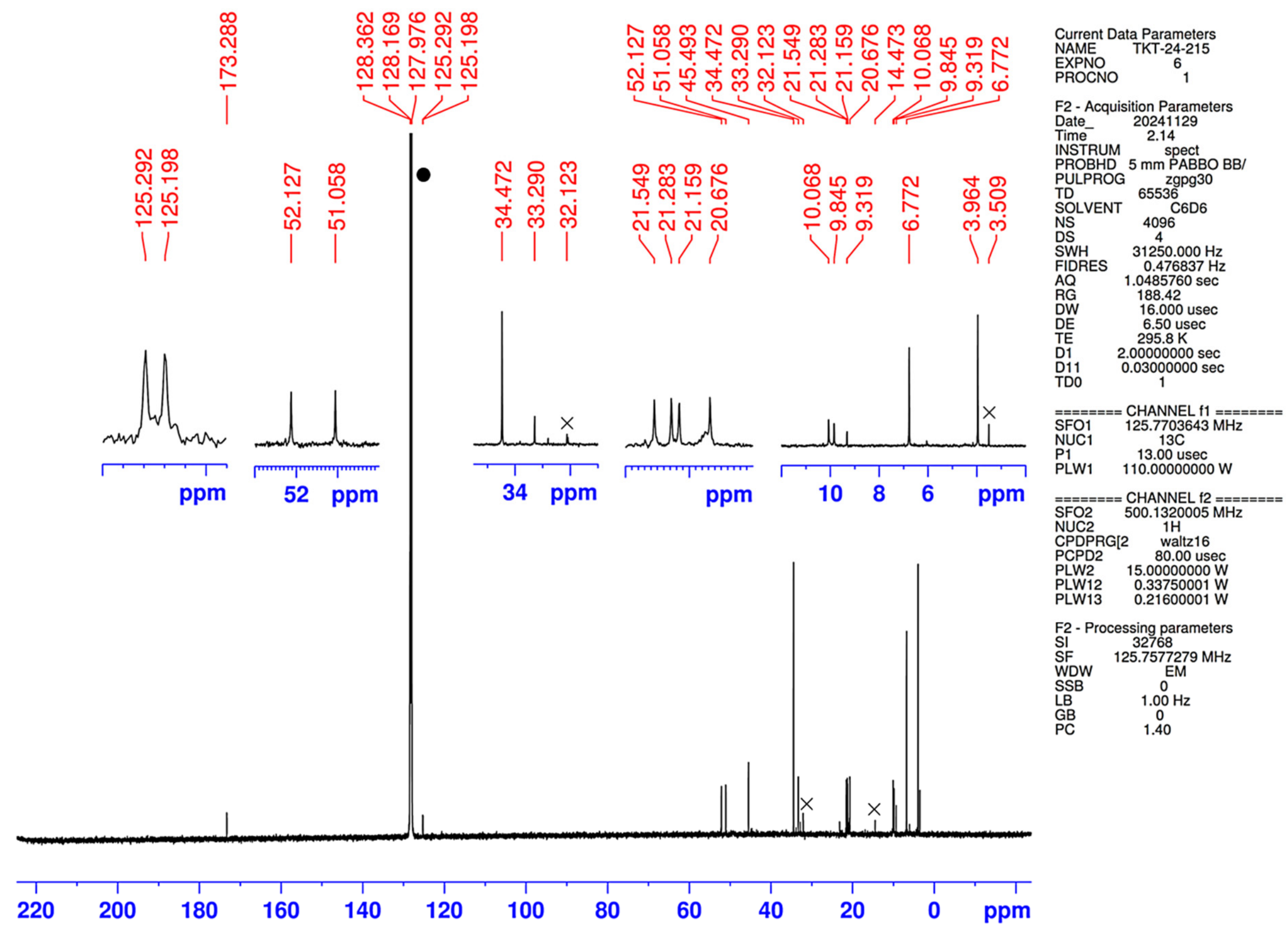

Figure S34. $^{13}C\{^{1}H\}$ CPD NMR spectrum of **13** in C_6D_6 at 296 K (● = C_6D_6, × = **12**).

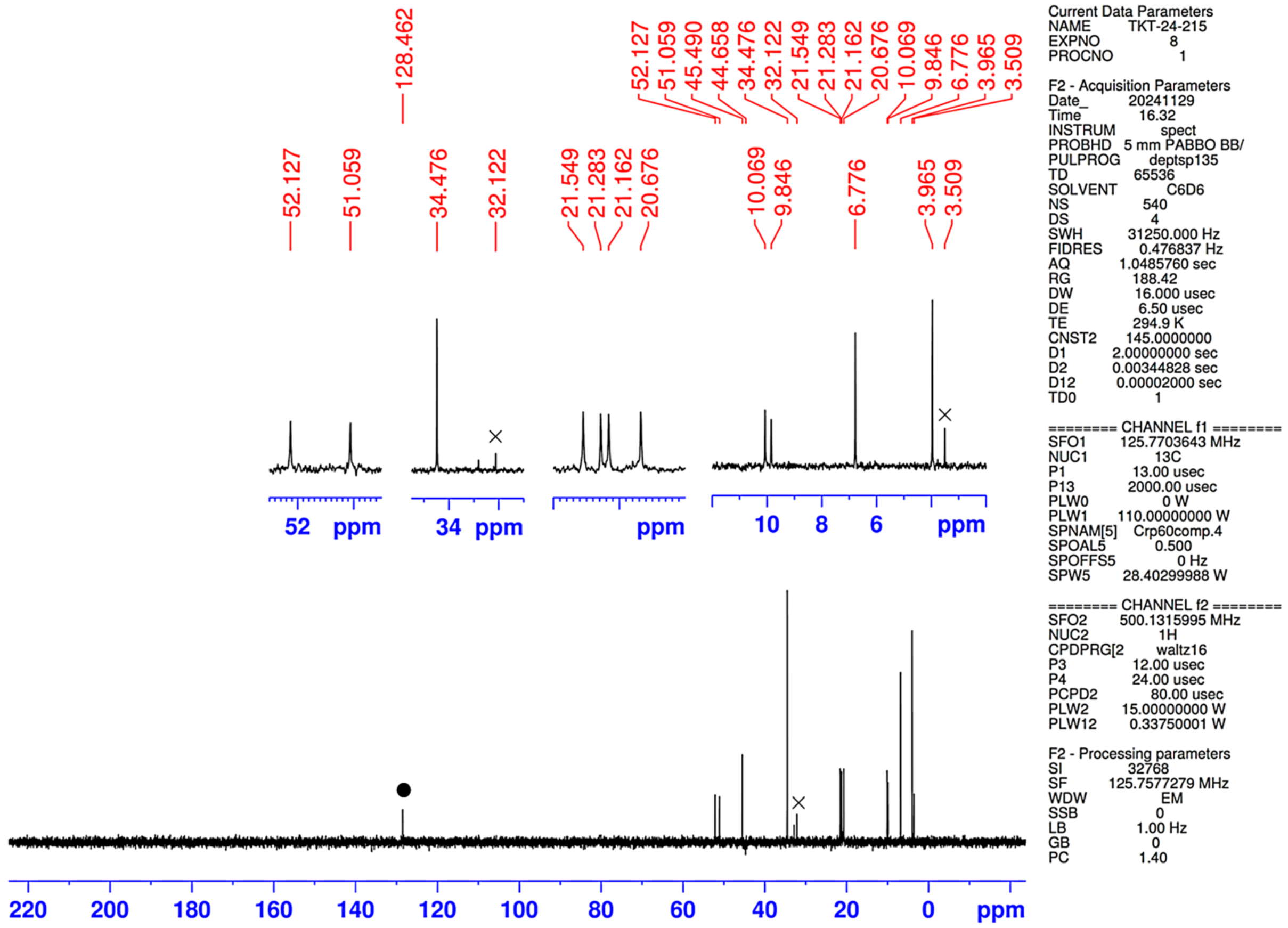

Figure S35. $^{13}C\{^1H\}$ DEPT 135 NMR spectrum of **13** in C_6D_6 at 295 K (● = C_6D_5H, × = **12**).

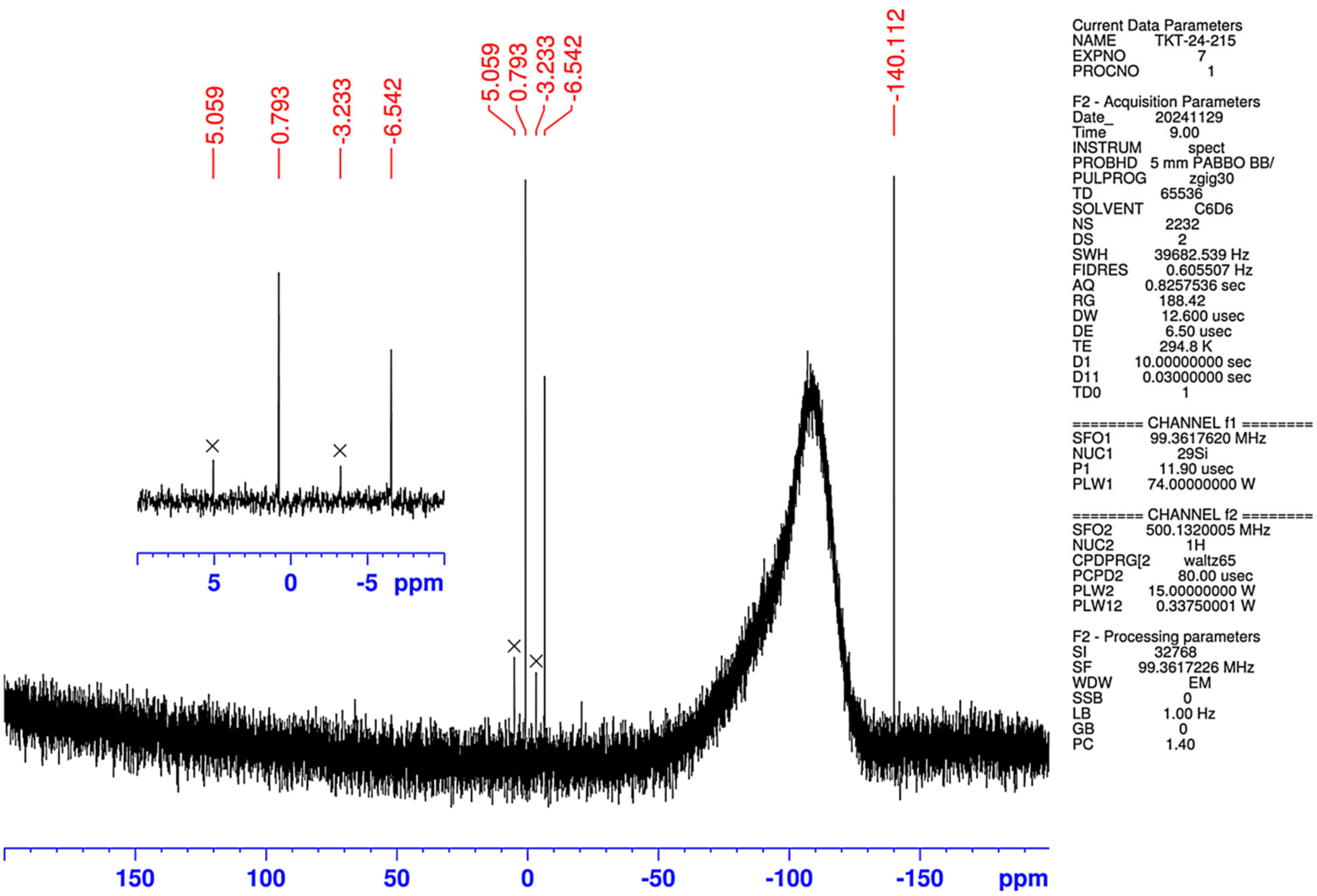

Figure S36. $^{29}Si\{^1H\}$ NMR spectrum of **13** in C_6D_6 at 295 K (× = **12**).

3. X-ray Structural Analysis

The single crystals suitable for data X-ray diffraction study were obtained by recrystallization using following conditions; from THF:Et_2O (1:1) solution at –25 °C for **3**, from THF:Et_2O (1:1) solution at –25 °C for **6**,.from toluene at –27 °C for **9b**, from Et_2O at –25 °C for **9c**, from hexane:THF (1:1) solution at –25 °C for **12** and from hexane:toluene (1:1) solution at –25 °C for **13**. A single crystal coated by Paratone® N oil for data collection was mounted on the glass fiber and then transferred to the cold nitrogen gas stream of the diffractometer. X-ray diffraction data were collected on a Bruker AXS APEXII diffractometer with a CCD detector or a Rigaku Synergy DW diffractometer with a hybrid pixel array detector. An empirical absorption correction based on the multiple measurements of equivalent reflections was applied using the program SADABS[57] or based on the CrysAlisPro 1.171.42.98a (Rigaku Oxford Diffraction, 2023) empirical absorption correction using spherical harmonics, implemented in SCALE3 ABSPACK scaling algorithm. The structures were solved by direct methods and refined by full-matrix least squares against F^2 using all data (SHELXL-2018 and 2019).[58] The molecular structures were analyzed by Yadokari-XG software.[59,60]

Crystal data of **3** (100 K): $C_{31}H_{70}N_2SSi_6(C_4H_{10}O)$; Fw 745.61; monoclinic; $P2_1/n$, a = 9.0970(4) Å, b = 19.0960(7) Å, c = 26.1396(12) Å, β = 93.027(4)°, V = 4534.5(3) Å^3, Z = 4, $R1$ = 0.0657 ($I > 2\sigma(I)$), $wR2$ = 0.1586 (all data), GOF = 1.047.

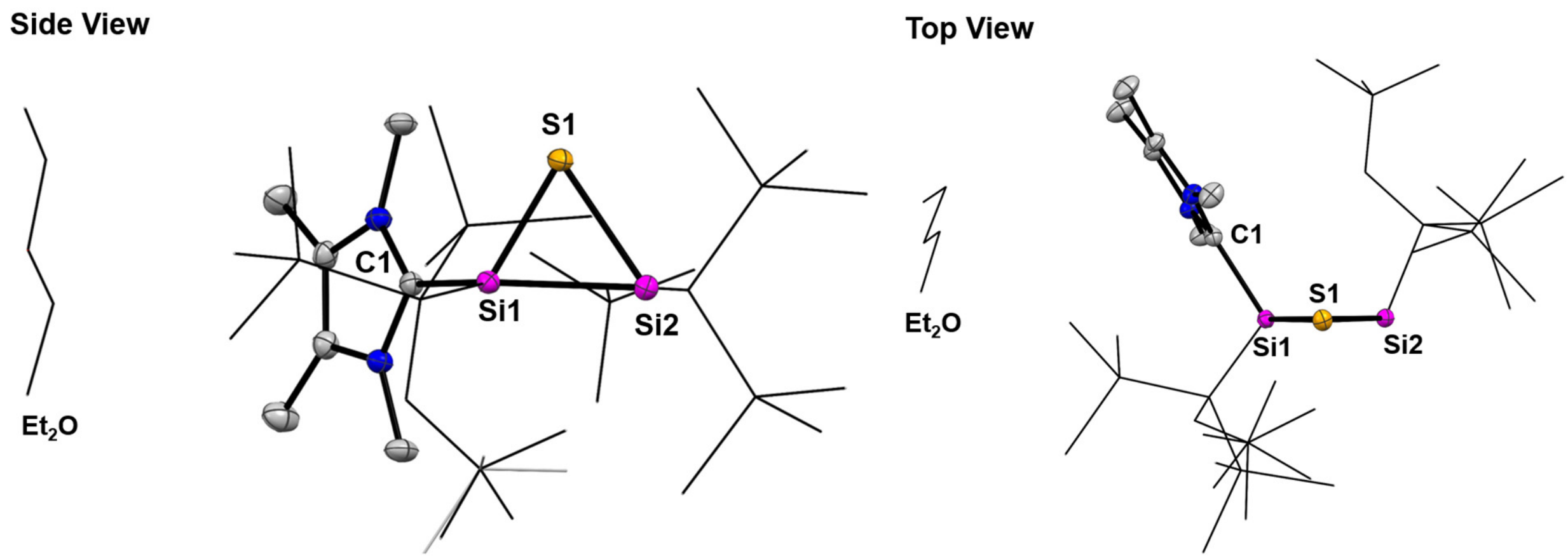

Figure S37. Molecular structure of **3** with thermal ellipsoids set at 50% probability. H atoms are omitted for clarity.

Crystal data of **6** (100 K): $C_{40}H_{79}N_3SSi_6$; Fw 802.66; monoclinic; $P2_1/n$, a = 9.7432(1) Å, b = 32.0626(3) Å, c = 14.9154(2) Å, β = 90.066(1)°, V = 4659.45(9) Å^3, Z = 4, $R1$ = 0.0323 ($I > 2\sigma(I)$), $wR2$ = 0.0877 (all data), GOF = 1.034.

Crystal data of **9b** (100 K): $C_{29}H_{68}N_2SSi_6$; Fw 645.45; monoclinic; $P2_1/n$, a = 9.1468(1) Å, b = 26.1899(2) Å, c = 15.7100(1) Å, β = 90.569(11)°, V = 2763.20(6) Å^3, Z = 4, $R1$ = 0.0315 ($I > 2\sigma(I)$), $wR2$ = 0.0827 (all data), GOF = 1.152.

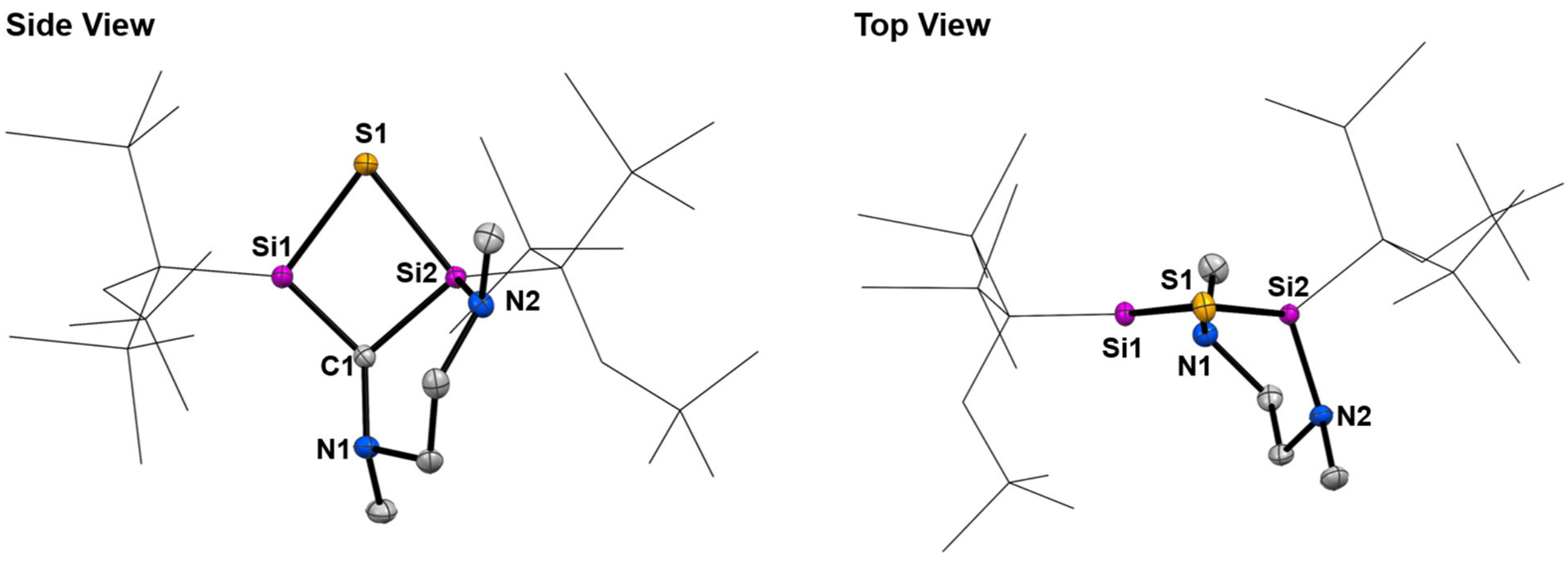

Figure S38. Molecular structure of **9b** with thermal ellipsoids set at 50% probability. H atoms are omitted for clarity.

Crystal data of **9c** (100 K): $C_{29}H_{67}N_1SSi_6$; Fw 630.44; monoclinic; $P2_1/n$, a = 16.5187(8) Å, b = 9.2982(5) Å, c = 24.270(3) Å, β = 92.308(9)°, V = 3724.7(5) Å^3, Z = 4, $R1$ = 0.0321 ($I > 2\sigma(I)$), $wR2$ = 0.0844 (all data), GOF = 1.043.

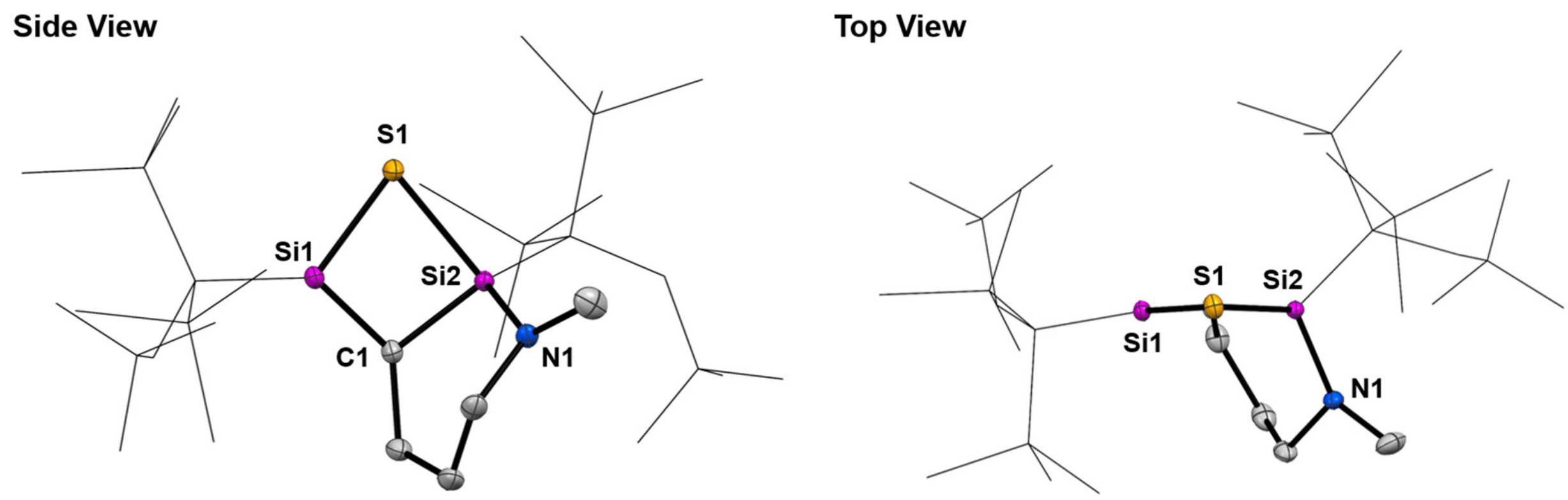

Figure S39. Molecular structure of **9c** with thermal ellipsoids set at 50% probability. H atoms are omitted for clarity.

Crystal data of **12** (100 K): $C_{24}H_{60}S_2Si_6$; Fw 581.38; triclinic; P-1, a = 8.9724(2) Å, b = 8.8958(2) Å, c = 12.1262(3) Å, α = 82.003(2)°, β = 80.245(2)°, γ = 67.341(2)°, V = 867.57(4) Å^3, Z = 1, $R1$ = 0.0365 ($I > 2\sigma(I)$), $wR2$ = 0.0996 (all data), GOF = 1.071. Selected bond length (Å) and bond angle (°): Si1−S1 2.1518(4), Si1−S1' 2.1526(5), Si1−S1−Si1' 83.17(2), S1−Si1−S1' 96.83(2).

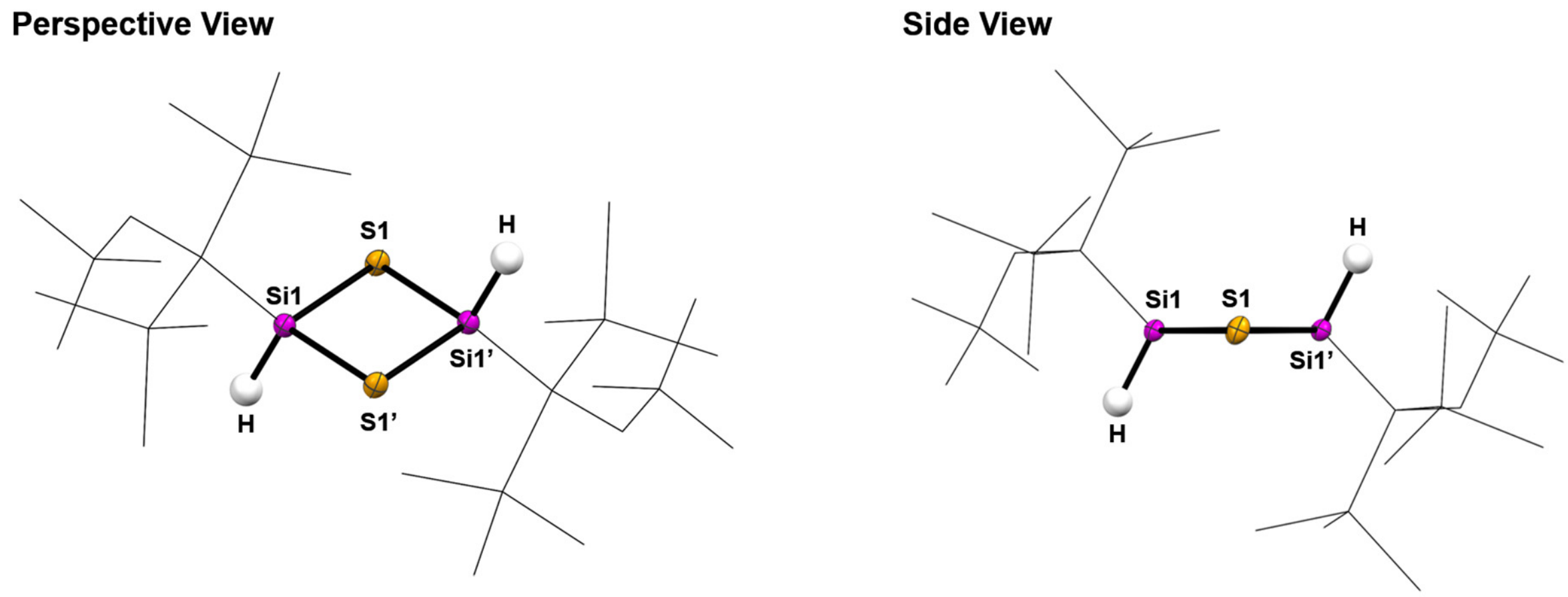

Figure S40. Molecular structure of **12** with thermal ellipsoids set at 50% probability. H atoms except for Si-H moiety are omitted for clarity.

Crystal data of **13** (300 K): $C_{23}H_{48}N_2Si_3$; Fw 436.90; monoclinic; $P2_1/c$, a = 16.0639(17) Å, b = 21.2673(9) Å, c = 17.0161(14) Å, β = 103.662(11)°, V = 5648.8(8) Å^3, Z = 8, $R1$ = 0.0734 ($I > 2\sigma(I)$), $wR2$ = 0.2025 (all data), GOF = 0.979. Note: X-ray diffraction data for **13** were collected at 300 K in air, and the single crystal decayed during the measurement. Consequently, only an incomplete data set with a low completeness of 72.8% (θ = 25.242°) was obtained. One A-level alert (PLAT029_ALERT_3_A) and one B-level alert (PLAT911_ALERT_3_B), both arising from the limited data set, were generated by the checkCIF system, and this issue was addressed in the validation response form (VRF) included in the CIF file. Although the connectivity of the molecule and overall structure of **13** determined by X-ray diffraction are consistent with the spectroscopic data, we do not discuss the metrical parameters of **13** because of the incomplete data set.

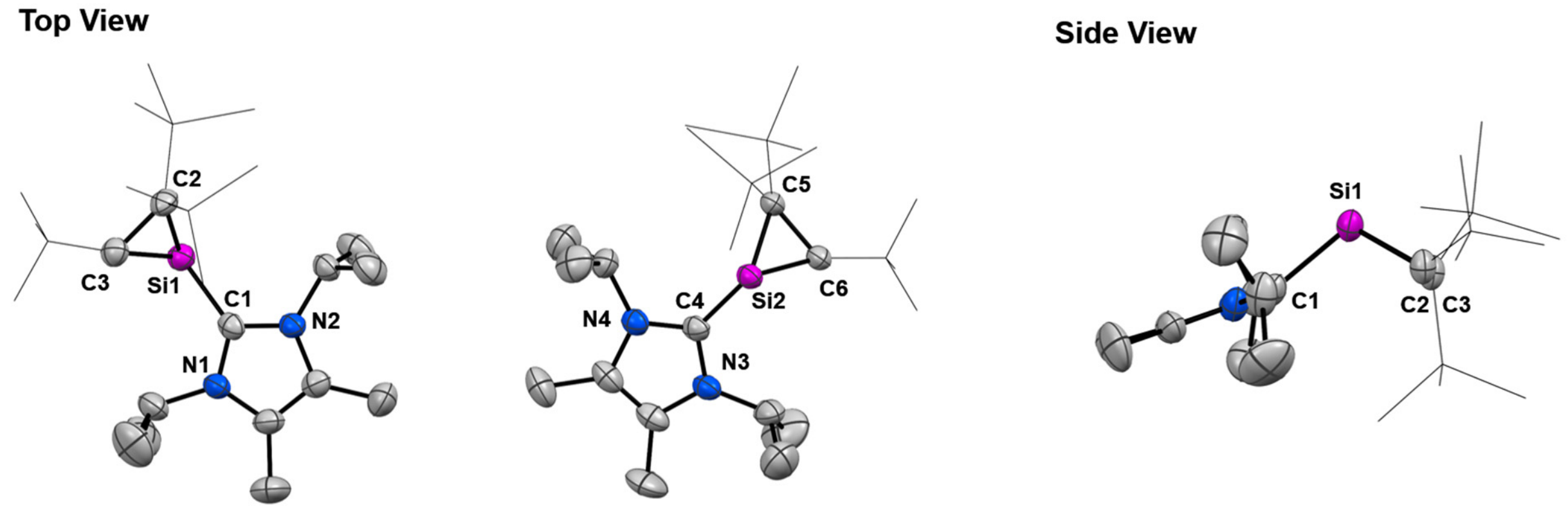

Figure S41. Molecular structure of **13** with thermal ellipsoids set at 50% probability. Two crystallographically independent molecules exist in the unit cell. Hydrogen atoms are omitted for clarity.

4. UV-vis Absorption Spectra

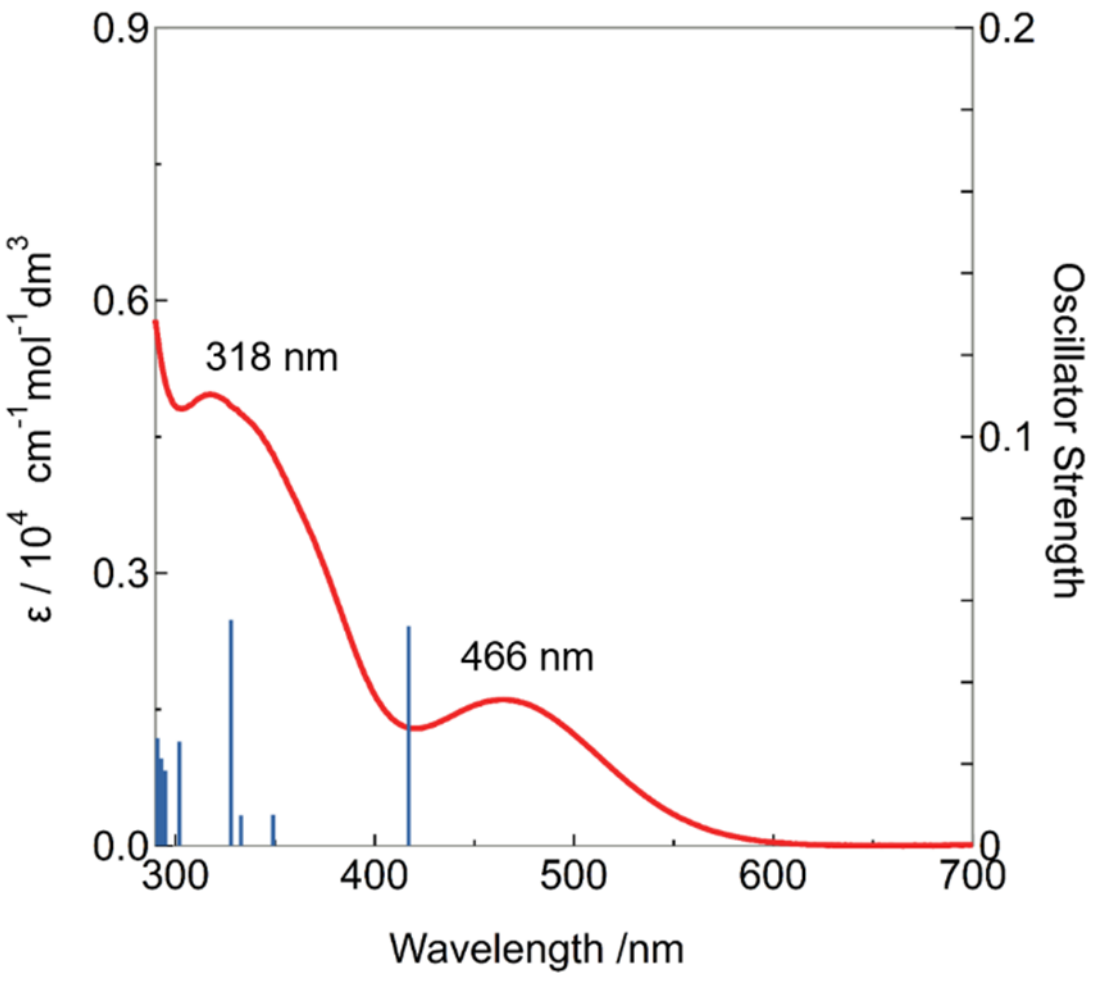

Figure S42. UV-vis absorption spectrum of **3** in benzene at rt. Superimposed vertical bars indicate band position and oscillator strength of $\mathbf{3}_{opt}$ calculated at the TD-M06-2X/6-311+G(2df,p)//B3PW91-D3/6-31G(d) level of theory. The calculated transitions and oscillator strength values are summarized in Table S1.

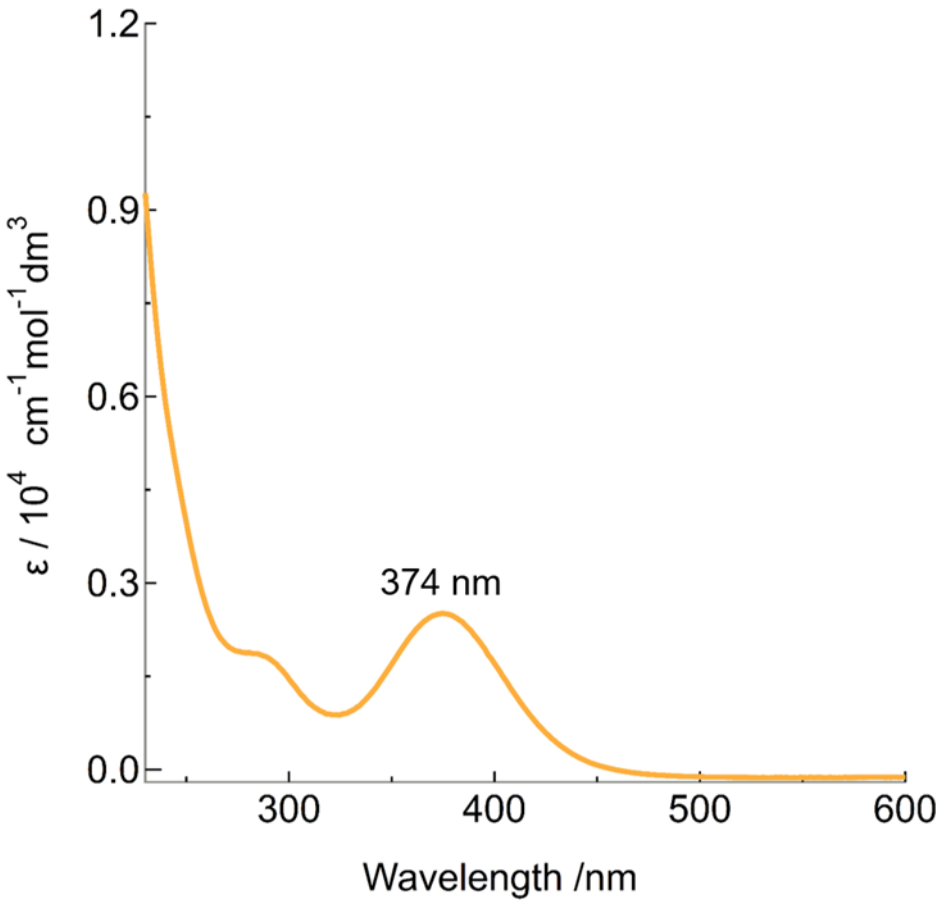

Figure S43. UV-vis absorption spectrum of **9b** in hexane at rt.

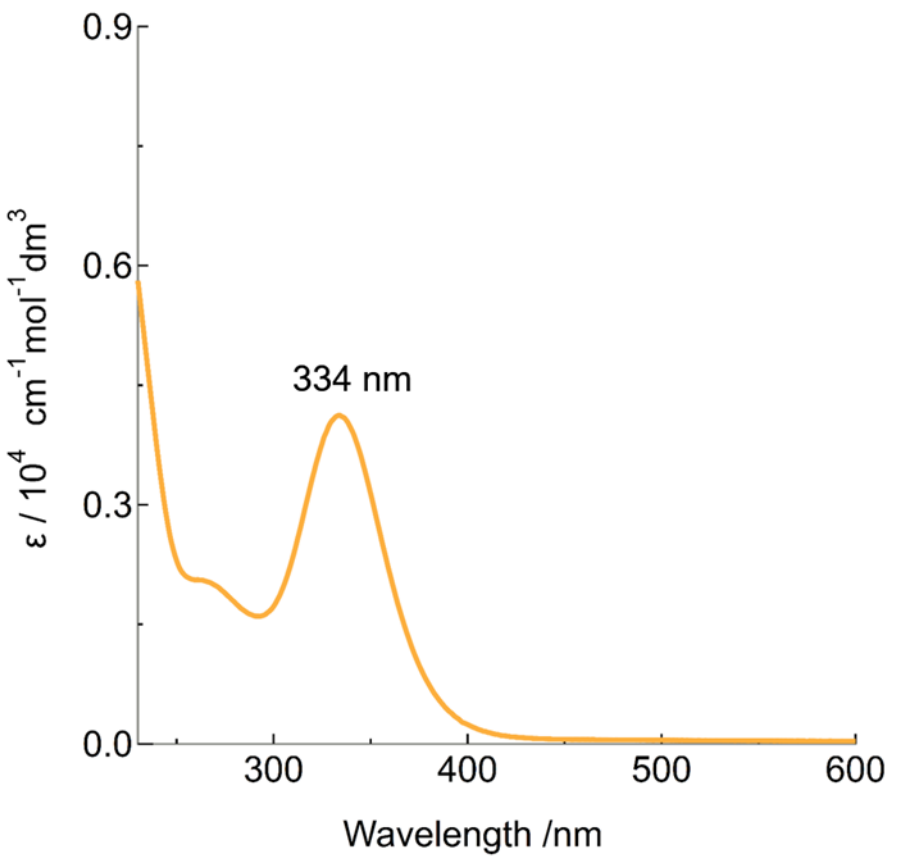

Figure S44. UV-vis absorption spectrum of **9c** in hexane at rt.

5. Theoretical Studies

All theoretical calculations were performed using Gaussian 09.[61] Geometry optimization and frequency analysis for all structures were performed at the B3PW91-D3/6-31G(d) level of theory. Initial transition states were calculated by using ColabReaction program with default settings,[62] and re-optimized at the B3PW91-D3/6-31G(d) level of theory. Imaginary frequencies were not encountered in any equilibrium structures, and one imaginary frequency was observed in the transition states. Connection of each TS is checked by IRC[63] jobs in the Gaussian 09 program. Gibbs free energies were estimated at 298.15 K and 1 atm pressure. Excitation energies and oscillator strengths of $\mathbf{3}_{\mathbf{opt}}$ were calculated at the M06-2X/6-311+G(2df,p) level of theory and are in good agreement with the experimental UV-Vis spectra. Mayer bond order (MBO) was calculated using the Multifunctional Wavefunction Analyzer (Multiwfn) 3.7 program.[64] Natural population analysis (NPA) and natural resonance theory (NRT) analysis was conducted by the NBO 7.0 program.[65]

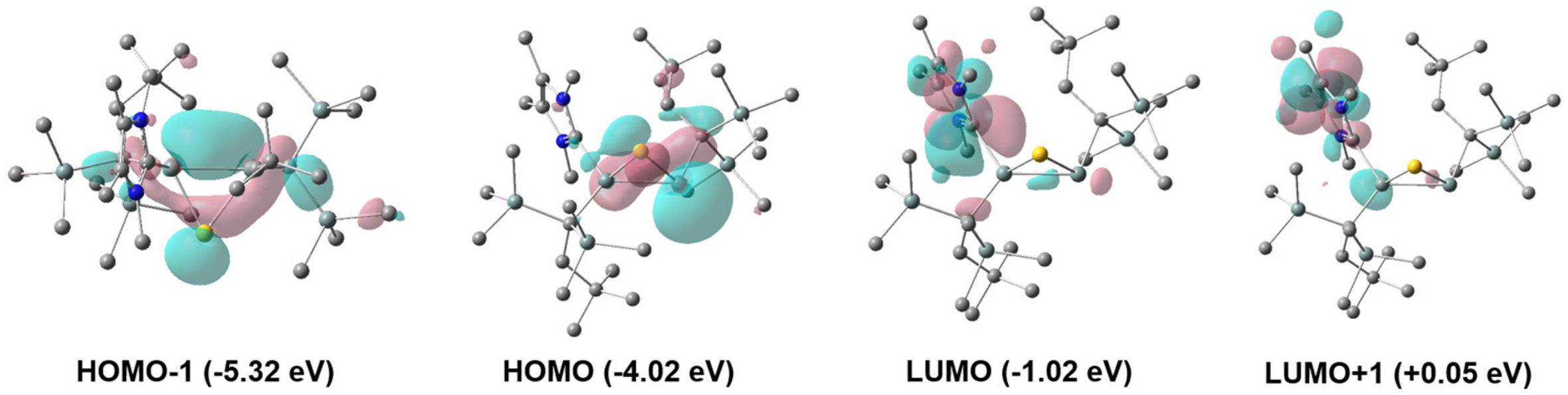

Figure S45. Kohn-Sham orbitals of $\mathbf{3}_{\mathbf{opt}}$ calculated at M06-2X/6-311+G(2df,p) level of theory (isosurface value: 0.04).

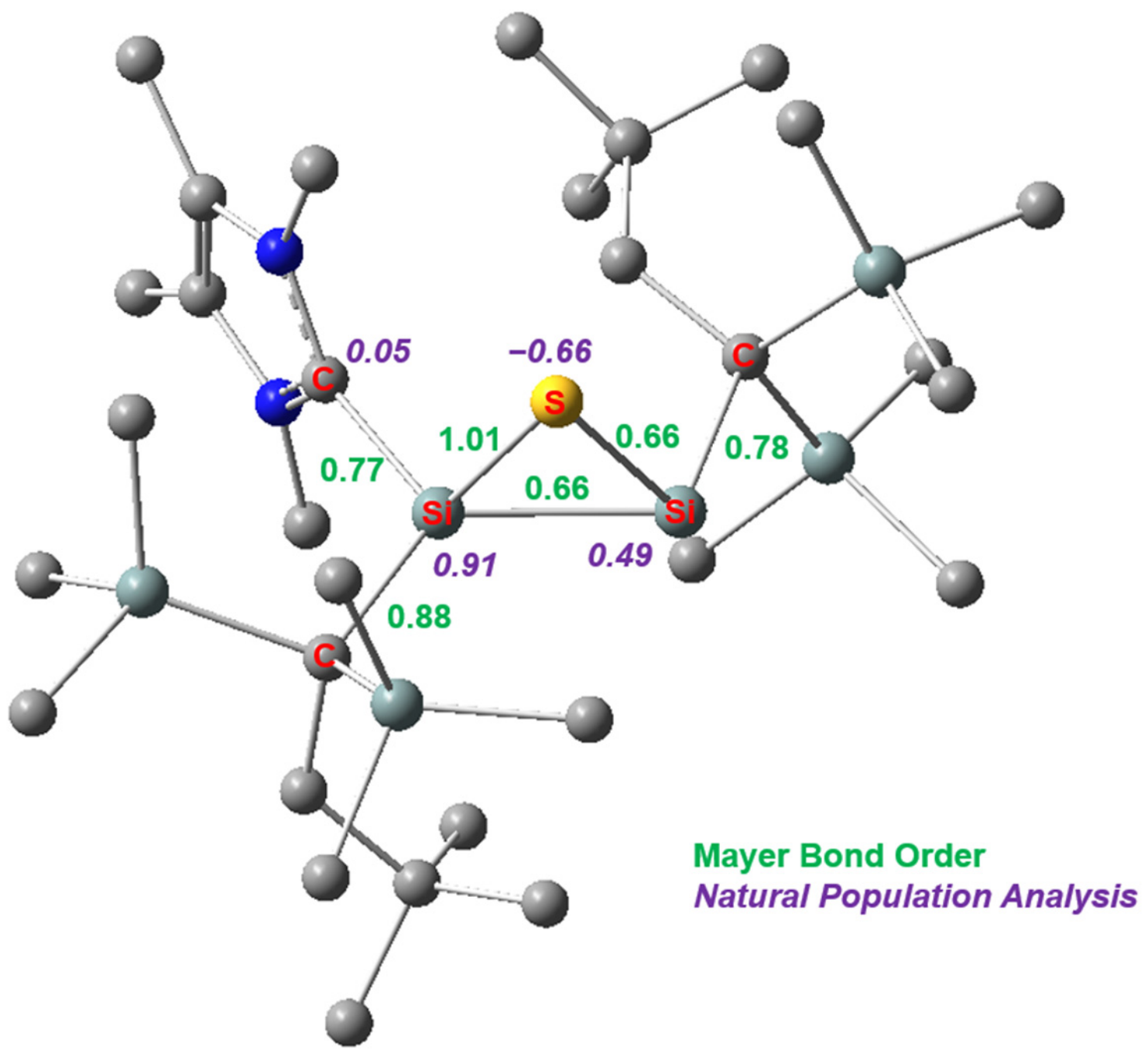

Figure S46. Mayer bond order and natural population analysis of the calculated $\mathbf{3}_{\mathbf{opt}}$.

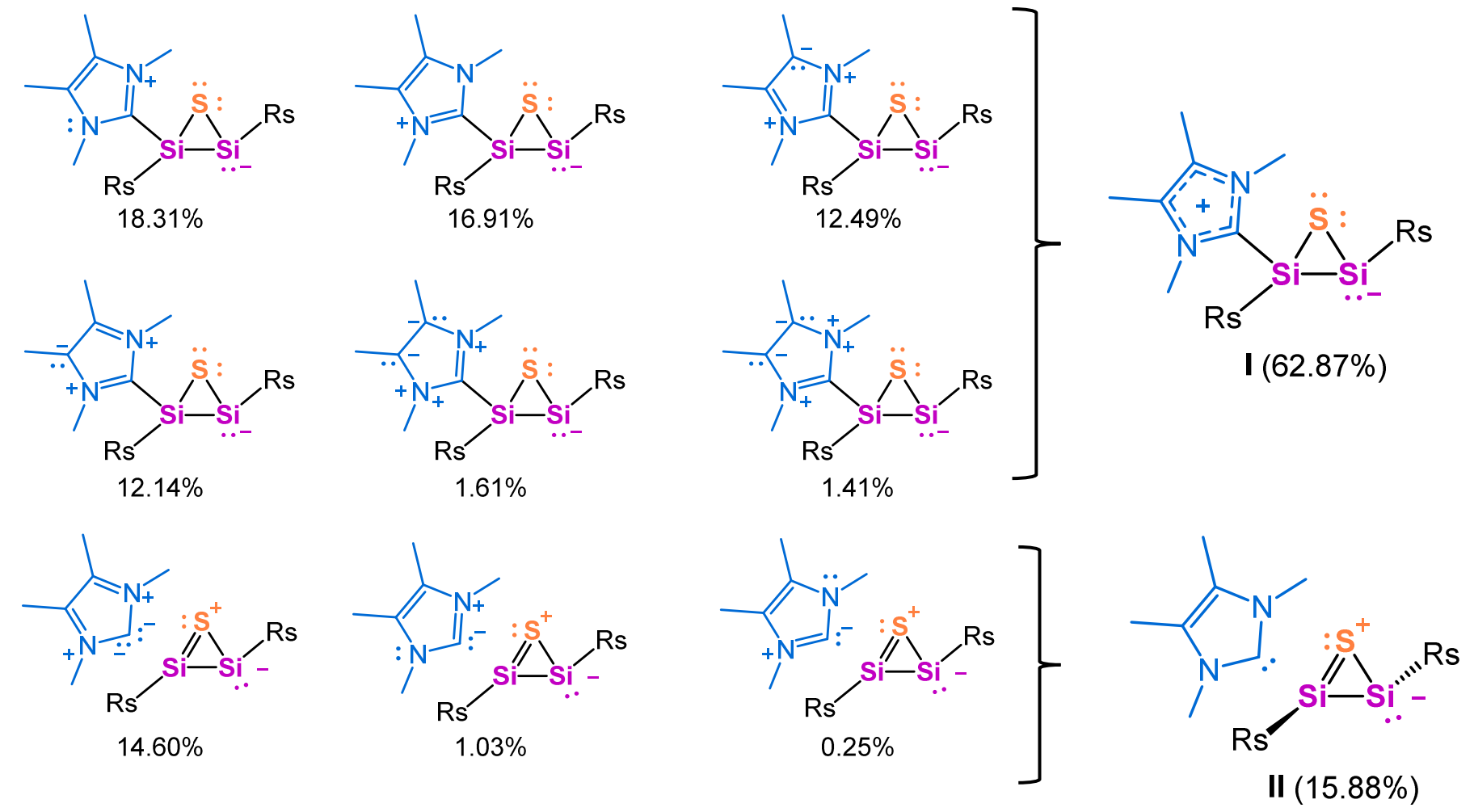

Figure S47. Major canonical structures of calculated $\mathbf{3_{opt}}$ obtained by NRT analysis.

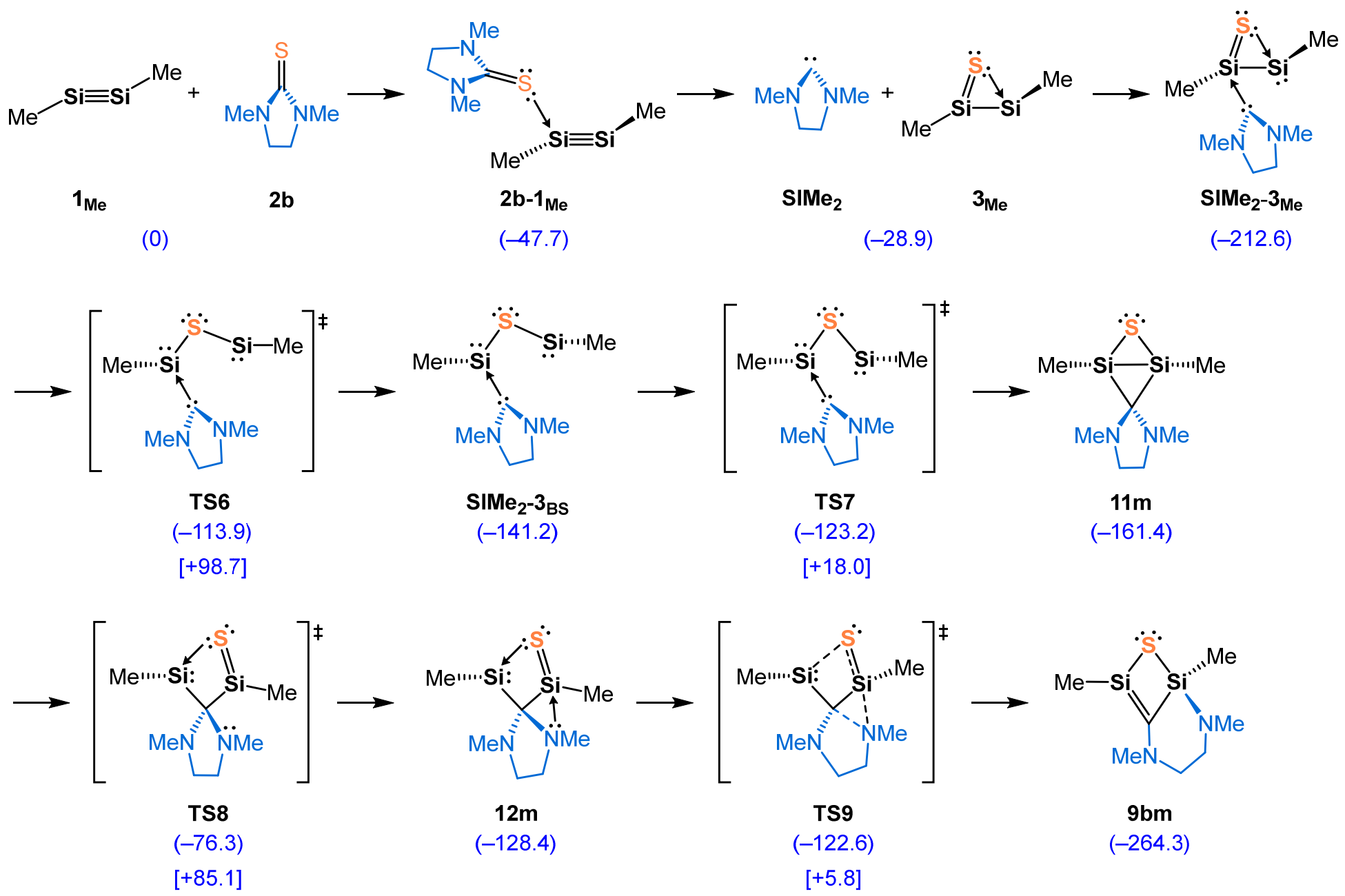

Figure S48. Gibbs energy diagrams of a plausible formation mechanism of cyclic silene **9bm** calculated at the B3PW91-D3/6-31G(d) level of theory. The overall sequence is energetically downhill. Simultaneous sulfur and NHC transfer from **2b** to $\mathbf{1_{Me}}$ affords NHC-coordinated silathioacyl silylene $\mathbf{SIMe_2\text{-}3_{Me}}$ via cyclic thiourea-coordinated disilyne $\mathbf{2b\text{-}1_{Me}}$. Subsequent Si–Si bond cleavage and S–Si bond rotation give NHC-coordinated 1,3-bis(silylene) intermediate $\mathbf{SIMe_2\text{-}3_{BS}}$, which isomerizes to disilathiabicyclo[1.1.0]butane **11m** and then to silanethione-coordinated silylene **12m**. Finally, a 1,2-nitrogen migration of **12m** furnishes cyclic silene **9bm**. Values in parentheses and brackets indicate relative Gibbs energies and Gibbs activation energies, respectively, in kJ mol^{-1}.

Table S1. Transition energy, wavelength, and oscillator strength of the electronic transition of $\mathbf{3_{opt}}$ calculated at the M06-2X/6-311+G(2df,p) level of theory.

```
Excited State   1:    Singlet-A      2.9757 eV  416.66 nm
f=0.0537  <S**2>=0.000
     185 -> 186      -0.16520
     185 -> 187       0.64173
     185 -> 189       0.18860
This state for optimization and/or second-order correction.
Total Energy, E(TD-HF/TD-KS) = -3467.91801022
Copying the excited state density for this state as the 1-
particle RhoCI density.

Excited State   2:    Singlet-A      3.5474 eV  349.50 nm
f=0.0076  <S**2>=0.000
     185 -> 188       0.16788
     185 -> 189      -0.10137
     185 -> 193       0.17153
     185 -> 195      -0.12087
     185 -> 196       0.26489
     185 -> 197      -0.12264
     185 -> 199       0.11211
     185 -> 201      -0.16686
     185 -> 203       0.24179
     185 -> 204       0.22658
     185 -> 205       0.25069
     185 -> 206       0.18027
     185 -> 207       0.12310

Excited State   3:    Singlet-A      3.7178 eV  333.49 nm
f=0.0074  <S**2>=0.000
     185 -> 186       0.62836
     185 -> 187       0.15423
     185 -> 188       0.16331

Excited State   4:    Singlet-A      3.7840 eV  327.65 nm
f=0.0553  <S**2>=0.000
     184 -> 187       0.12984
     185 -> 186       0.23073
     185 -> 188      -0.30318
     185 -> 194       0.19279
     185 -> 196       0.34415
     185 -> 198      -0.12626
     185 -> 204       0.14223
     185 -> 205      -0.13689
     185 -> 206      -0.14467
     185 -> 216      -0.13037

Excited State   5:    Singlet-A      4.0981 eV  302.54 nm
f=0.0255  <S**2>=0.000
     184 -> 186      -0.10762
     184 -> 187       0.39119
     184 -> 189       0.11679
     185 -> 192      -0.13220
     185 -> 198       0.13441
     185 -> 205      -0.15784
     185 -> 207      -0.14625
     185 -> 212      -0.23796
     185 -> 213      -0.12589
     185 -> 215       0.10086
     185 -> 216       0.27890

Excited State   6:    Singlet-A      4.1395 eV  299.51 nm
f=0.0015  <S**2>=0.000
     184 -> 187       0.19116
     185 -> 188       0.54994
     185 -> 196       0.11398
     185 -> 203      -0.10647
     185 -> 205      -0.13165
     185 -> 206      -0.12947
     185 -> 212       0.11962
     185 -> 216      -0.13311

Excited State   7:    Singlet-A      4.2097 eV  294.52 nm
f=0.0184  <S**2>=0.000
     184 -> 187      -0.29696
     185 -> 187       0.14415
     185 -> 188       0.13951
     185 -> 189      -0.34032
     185 -> 190       0.35081
     185 -> 196       0.10686
     185 -> 205      -0.14616
     185 -> 207      -0.10930

Excited State   8:    Singlet-A      4.2307 eV  293.06 nm
f=0.0213  <S**2>=0.000
     184 -> 187       0.14058
     185 -> 188      -0.10253
     185 -> 189       0.22400
     185 -> 190       0.50087
     185 -> 195       0.23959
     185 -> 216      -0.11164

Excited State   9:    Singlet-A      4.2544 eV  291.43 nm
f=0.0263  <S**2>=0.000
     184 -> 186       0.10886
     184 -> 187      -0.29025
     185 -> 189       0.50233
     185 -> 192      -0.10728
     185 -> 196       0.21583
     185 -> 205      -0.11661

Excited State  10:    Singlet-A      4.4506 eV  278.58 nm
f=0.0050  <S**2>=0.000
     185 -> 190      -0.22214
     185 -> 191       0.33608
     185 -> 192       0.17081
     185 -> 193       0.31126
     185 -> 195       0.32286
     185 -> 196       0.11550
     185 -> 201       0.17210

Excited State  11:    Singlet-A      4.5575 eV  272.04 nm
f=0.0018  <S**2>=0.000
     185 -> 191      -0.27848
     185 -> 192       0.25701
     185 -> 194       0.38353
     185 -> 195       0.13653
     185 -> 198       0.12101
     185 -> 199      -0.13627
     185 -> 202      -0.13629
     185 -> 216       0.16807

Excited State  12:    Singlet-A      4.5751 eV  271.00 nm
f=0.0017  <S**2>=0.000
     185 -> 191       0.35344
     185 -> 192      -0.32974
     185 -> 193      -0.18908
     185 -> 194       0.42759

Excited State  13:    Singlet-A      4.6038 eV  269.31 nm
f=0.0007  <S**2>=0.000
     185 -> 190       0.14445
     185 -> 191       0.35032
     185 -> 192       0.43144
     185 -> 193      -0.22570
     185 -> 195      -0.19215
     185 -> 216       0.11240

Excited State  14:    Singlet-A      4.6308 eV  267.74 nm
f=0.0011  <S**2>=0.000
     185 -> 190       0.13702
     185 -> 191       0.10669
     185 -> 193       0.48096
```

```
     185 -> 194       0.22196
     185 -> 195      -0.31841
     185 -> 199      -0.12553
     185 -> 204      -0.14224

 Excited State  15:   Singlet-A      4.6995 eV  263.83 nm
 f=0.0059  <S**2>=0.000
     184 -> 196      -0.12924
     185 -> 192       0.17714
     185 -> 193       0.10490
     185 -> 194       0.12464
     185 -> 196      -0.35290
     185 -> 198      -0.19701
     185 -> 199       0.16926
     185 -> 201      -0.11637
     185 -> 204       0.37678
     185 -> 207      -0.12023

 Excited State  16:   Singlet-A      4.8376 eV  256.29 nm
 f=0.0522  <S**2>=0.000
     183 -> 187      -0.10031
     184 -> 196       0.29166
     184 -> 197      -0.10336
     184 -> 204       0.17269
     185 -> 197      -0.18914
     185 -> 199       0.12381
     185 -> 200       0.21343
     185 -> 203      -0.19763
     185 -> 204       0.12835
     185 -> 223       0.14440

 Excited State  17:   Singlet-A      4.8968 eV  253.20 nm
 f=0.0677  <S**2>=0.000
     183 -> 187      -0.14141
     184 -> 188      -0.13279
     184 -> 194       0.10998
     184 -> 196       0.31304
     184 -> 204       0.14989
     185 -> 195       0.13826
     185 -> 197       0.10646
     185 -> 200      -0.26769
     185 -> 201      -0.13682
     185 -> 202       0.13526
     185 -> 203       0.15731
     185 -> 209       0.10271

 Excited State  18:   Singlet-A      4.9251 eV  251.74 nm
 f=0.0110  <S**2>=0.000
     185 -> 197       0.38276
     185 -> 198       0.26426
     185 -> 199       0.40660
     185 -> 202      -0.18084

 Excited State  19:   Singlet-A      4.9433 eV  250.81 nm
 f=0.0026  <S**2>=0.000
     184 -> 186       0.47390
     184 -> 187       0.12871
     185 -> 197       0.21821
     185 -> 199      -0.13040
     185 -> 200       0.26807
     185 -> 201       0.14821

 Excited State  20:   Singlet-A      4.9597 eV  249.98 nm
 f=0.0077  <S**2>=0.000
     184 -> 186       0.45244
     184 -> 187       0.11555
     185 -> 197      -0.27325
     185 -> 198       0.11089
     185 -> 199       0.14714
     185 -> 200      -0.25012
     185 -> 201      -0.13543
     185 -> 204      -0.12340
     185 -> 205       0.10991

 Excited State  21:   Singlet-A      4.9784 eV  249.04 nm
 f=0.0171  <S**2>=0.000
     185 -> 195      -0.30162
     185 -> 196      -0.11728
     185 -> 200      -0.17343
     185 -> 201       0.39672
     185 -> 202       0.13590
     185 -> 203       0.24534
     185 -> 204       0.11560
     185 -> 206       0.10016
     185 -> 218      -0.10680

 Excited State  22:   Singlet-A      5.0227 eV  246.85 nm
 f=0.0230  <S**2>=0.000
     184 -> 188      -0.25202
     184 -> 193      -0.11453
     184 -> 203      -0.12931
     184 -> 205      -0.16022
     184 -> 206      -0.11392
     185 -> 197      -0.19142
     185 -> 198       0.38521
     185 -> 200       0.18265
     185 -> 202       0.18287
     185 -> 216      -0.11072

 Excited State  23:   Singlet-A      5.0354 eV  246.22 nm
 f=0.0325  <S**2>=0.000
     183 -> 187      -0.18501
     184 -> 188       0.26054
     184 -> 193       0.10007
     184 -> 194      -0.11269
     184 -> 203       0.12480
     184 -> 205       0.15321
     184 -> 206       0.12037
     185 -> 198       0.22098
     185 -> 200       0.17220
     185 -> 201      -0.18903
     185 -> 202       0.30993

 Excited State  24:   Singlet-A      5.0535 eV  245.34 nm
 f=0.0493  <S**2>=0.000
     182 -> 187      -0.11566
     183 -> 186      -0.13064
     183 -> 187       0.49454
     183 -> 189       0.14850
     184 -> 196       0.10104
     185 -> 199       0.20478
     185 -> 200       0.11629
     185 -> 202       0.26298
     185 -> 204      -0.11728

 Excited State  25:   Singlet-A      5.0719 eV  244.46 nm
 f=0.0426  <S**2>=0.000
     183 -> 187       0.25855
     184 -> 196       0.11692
     185 -> 198       0.22466
     185 -> 199      -0.30905
     185 -> 200      -0.18128
     185 -> 202      -0.14502
     185 -> 203      -0.12976
     185 -> 204       0.33244

 Excited State  26:   Singlet-A      5.1100 eV  242.63 nm
 f=0.0064  <S**2>=0.000
     185 -> 196      -0.18187
     185 -> 200       0.12813
     185 -> 201      -0.20598
     185 -> 202      -0.28954
     185 -> 203       0.40432
     185 -> 205      -0.21400
     185 -> 206      -0.12578
     185 -> 216      -0.13366

 Excited State  27:   Singlet-A      5.1677 eV  239.92 nm
 f=0.0113  <S**2>=0.000
```

```
    184 -> 188       0.13606
    185 -> 197      -0.20325
    185 -> 200       0.14136
    185 -> 201       0.21378
    185 -> 202      -0.16767
    185 -> 205       0.15797
    185 -> 207      -0.14497
    185 -> 209       0.17514
    185 -> 218       0.12957
    185 -> 219      -0.14230
    185 -> 221       0.10257
    185 -> 223      -0.17380
    185 -> 224       0.13226
    185 -> 226       0.16349
    185 -> 227      -0.10830
    185 -> 229      -0.11527

Excited State  28:   Singlet-A      5.2613 eV  235.65 nm
f=0.0042  <S**2>=0.000
    185 -> 202      -0.11082
    185 -> 203      -0.16164
    185 -> 205      -0.30579
    185 -> 206       0.39897
    185 -> 207       0.13158
    185 -> 217       0.12631
    185 -> 219       0.10041
    185 -> 223      -0.16109
    185 -> 226       0.14542

Excited State  29:   Singlet-A      5.3426 eV  232.07 nm
f=0.0195  <S**2>=0.000
    184 -> 205      -0.10975
    184 -> 212      -0.16385
    184 -> 216       0.20144
    185 -> 205      -0.10023
    185 -> 207       0.28767
    185 -> 208      -0.16384
    185 -> 210       0.12886
    185 -> 212      -0.23309
    185 -> 214       0.11256
    185 -> 218       0.13184

Excited State  30:   Singlet-A      5.3814 eV  230.40 nm
f=0.0082  <S**2>=0.000
    184 -> 188       0.33733
    184 -> 189       0.12136
    184 -> 192       0.12413
    184 -> 198      -0.11825
    184 -> 201       0.10655
    184 -> 206      -0.11207
    184 -> 212       0.23338
    184 -> 216      -0.26453
    185 -> 207       0.18238
    185 -> 208      -0.11664

Excited State  31:   Singlet-A      5.4126 eV  229.06 nm
f=0.0147  <S**2>=0.000
    182 -> 187      -0.10175
    184 -> 188       0.39786
    184 -> 190       0.10142
    184 -> 193      -0.10789
    184 -> 205      -0.23934
    184 -> 207      -0.15411
    184 -> 213      -0.10079
    184 -> 216       0.11457
    185 -> 206       0.13477
    185 -> 208       0.20424

Excited State  32:   Singlet-A      5.4543 eV  227.31 nm
f=0.0057  <S**2>=0.000
    185 -> 203      -0.10278
    185 -> 207       0.15566
    185 -> 208       0.46683
    185 -> 210       0.16962
    185 -> 211       0.22903
    185 -> 213       0.16595
    185 -> 221       0.10300

Excited State  33:   Singlet-A      5.4637 eV  226.92 nm
f=0.0066  <S**2>=0.000
    184 -> 189      -0.12389
    184 -> 190       0.12012
    185 -> 201       0.10646
    185 -> 206      -0.17355
    185 -> 207       0.19585
    185 -> 208       0.17592
    185 -> 209       0.40516
    185 -> 211      -0.22534
    185 -> 214      -0.11088
    185 -> 221      -0.11688
    185 -> 225      -0.10271

Excited State  34:   Singlet-A      5.4716 eV  226.59 nm
f=0.0103  <S**2>=0.000
    184 -> 187      -0.16948
    184 -> 189       0.57986
    184 -> 190      -0.19615
    185 -> 208       0.10446

Excited State  35:   Singlet-A      5.4925 eV  225.74 nm
f=0.0014  <S**2>=0.000
    184 -> 190       0.14512
    185 -> 208      -0.18787
    185 -> 210       0.54915
    185 -> 211       0.12186
    185 -> 212       0.11393
    185 -> 213      -0.14852

Excited State  36:   Singlet-A      5.5110 eV  224.98 nm
f=0.0040  <S**2>=0.000
    184 -> 189       0.15014
    184 -> 190       0.26213
    185 -> 207       0.17834
    185 -> 208      -0.15257
    185 -> 209       0.20328
    185 -> 210      -0.22158
    185 -> 211       0.40462

Excited State  37:  Singlet-A      5.5212 eV  224.56 nm
f=0.0021  <S**2>=0.000
    182 -> 187       0.14038
    184 -> 189       0.12476
    184 -> 190       0.48226
    184 -> 195       0.12732
    185 -> 208       0.17269
    185 -> 211      -0.18947
    185 -> 212      -0.16004
    185 -> 214       0.10617

Excited State  38:   Singlet-A      5.5365 eV  223.94 nm
f=0.0039  <S**2>=0.000
    182 -> 187       0.13590
    185 -> 205      -0.13368
    185 -> 206      -0.16197
    185 -> 209      -0.17431
    185 -> 212       0.18675
    185 -> 213       0.34109
    185 -> 214      -0.22532
    185 -> 216       0.25952
    185 -> 224       0.12043
    185 -> 229      -0.11404

Excited State  39:   Singlet-A      5.5555 eV  223.17 nm
f=0.0105  <S**2>=0.000
    185 -> 205       0.10009
    185 -> 206      -0.22920
    185 -> 207       0.17381
    185 -> 208       0.11079
    185 -> 209      -0.25099
    185 -> 211       0.16022
```

185 -> 213 -0.25624
185 -> 214 -0.13118
185 -> 215 0.28109
185 -> 223 -0.12668

Excited State 40: Singlet-A 5.5677 eV 222.68 nm
f=0.0211 <S**2>=0.000
180 -> 187 0.11023
181 -> 187 0.13458
182 -> 186 -0.10902
182 -> 187 0.44913
182 -> 189 0.13764
183 -> 187 0.11288
184 -> 190 -0.11273
185 -> 207 0.11221
185 -> 213 -0.18557
185 -> 214 0.12364
